# A targeted bioinformatics approach identifies highly variable cell surface proteins that are unique to Glomeromycotina

**DOI:** 10.1007/s00572-021-01066-x

**Published:** 2022-01-15

**Authors:** Carolyn J. Schultz, Yue Wu, Ute Baumann

**Affiliations:** School of Agriculture, Food, and Wine, Waite Research Institute, University of Adelaide, Adelaide, SA Australia

**Keywords:** Arbuscular mycorrhizal fungi, Proline- and glycine-rich, Glycosylphosphatidylinositol (GPI)-anchored proteins, Tandem repeats, Intrinsically disordered proteins (IDPs), Glomeromycotina

## Abstract

Diversity in arbuscular mycorrhizal fungi (AMF) contributes to biodiversity and resilience in natural environments and healthy agricultural systems. Functional complementarity exists among species of AMF in symbiosis with their plant hosts, but the molecular basis of this is not known. We hypothesise this is in part due to the difficulties that current sequence assembly methodologies have assembling sequences for intrinsically disordered proteins (IDPs) due to their low sequence complexity. IDPs are potential candidates for functional complementarity because they often exist as extended (non-globular) proteins providing additional amino acids for molecular interactions. *Rhizophagus irregularis* arabinogalactan-protein-like proteins (AGLs) are small secreted IDPs with no known orthologues in AMF or other fungi. We developed a targeted bioinformatics approach to identify highly variable AGLs/IDPs in RNA-sequence datasets. The approach includes a modified multiple *k*-mer assembly approach (Oases) to identify candidate sequences, followed by targeted sequence capture and assembly (mirabait-mira). All AMF species analysed, including the ancestral family Paraglomeraceae, have small families of proteins rich in disorder promoting amino acids such as proline and glycine, or glycine and asparagine. Glycine- and asparagine-rich proteins also were found in *Geosiphon pyriformis* (an obligate symbiont of a cyanobacterium), from the same subphylum (Glomeromycotina) as AMF. The sequence diversity of AGLs likely translates to functional diversity, based on predicted physical properties of tandem repeats (elastic, amyloid, or interchangeable) and their broad pI ranges. We envisage that AGLs/IDPs could contribute to functional complementarity in AMF through processes such as self-recognition, retention of nutrients, soil stability, and water movement.

**Supplementary information:**

The online version contains supplementary material available at 10.1007/s00572-021-01066-x.

## Introduction

Arbuscular mycorrhizal fungi (AMF) are widespread symbionts that colonise 70–80% of living vascular plants providing nutrients (especially phosphorus) in exchange for plant-derived carbon. AMF comprise an ancient, diverse phylum of fungi whose taxonomy still is actively debated (Glomeromycotina (Spatafora et al. 2016) vs. Glomeromycota (Tedersoo et al. 2018)). New roles continue to emerge for AMF such as hyphal highways to transport motile bacterial to phosphorus-rich patches in soil (Jiang et al. 2021). Many studies have revealed strong links among fungal diversity, plant diversity, and plant health (Jansa et al. 2008; Davison et al. 2021); however, the obligate symbiotic nature of AMF has hampered research. In natural environments and well-managed agricultural systems, plants are colonised by multiple AMF species at the same time, suggesting that different benefits can be provided by different species (Koide 2000; Jansa et al. 2008; Higo et al. 2020). A recent global study of soils collected from natural ecosystems showed that different AMF species, especially in the family Glomeraceae, have defined ecological niches within both pH and temperature gradients (Davison et al. 2021). In contrast, other AMF families were specialised, with Acaulosporaceae preferring low pH and low temperature conditions, whereas Gigasporaceae preferred high precipitation conditions.

Species from the family Glomeraceae (*Rhizophagus* and *Glomus*) dominate many ecological studies (e.g., Higo et al. 2020). One species, *Rhizophagus irregularis*, has been the focus of many laboratory-based studies as it is amenable to root organ cultures (St-Arnaud et al. 1996) and is a successful coloniser and phosphorus provider compared to other species in pot and root organ culture (Thonar et al. 2011). The reasons for the success of *R. irregularis* are unknown, but we speculate that a small family of secreted proteins called AGLs (for arabinogalactan-protein (AGP)-like (Schultz and Harrison 2008)) are a contributing factor. When three AGLs cDNAs were identified in expressed sequenced tag (EST) libraries derived from *R. irregularis* colonised roots of *Medicago truncatula*, it was initially assumed that they represented plant AGP genes. However, the absence of AGLs from the *M. truncatula* genome, failure to be amplified from *M. truncatula* genomic DNA, but successful amplification from *R. irregularis* DNA confirmed the fungal origin of *RiAGLs* (Schultz and Harrison 2008). This finding was substantiated when the first AMF genome was published in 2013 (Tisserant et al. 2013).

AGLs are predicted to be secreted proteins based on the presence of both an N-terminal endoplasmic reticulum (ER) signal sequence and a C-terminal glycosylphosphatidyl inositol (GPI)-anchor signal for attachment to the outer leaflet of the fungal plasma membrane (Schultz and Harrison 2008; Yeats et al. 2018). Many GPI-anchored proteins are released from the plasma membrane as soluble proteins by phospholipases (Müller, 2018), thus they are versatile cell surface proteins that can potentially exist as membrane-bound and/or soluble forms. Additionally, in some Ascomycetes, e.g., *Saccharomyces cerevisiae* and *Candida albicans*, a subset of proteins with GPI-anchor signals are covalently linked to fungal walls through a modified lipid anchor to β-1,6-glucan (Klis et al. 2010; Yoko-o et al. 2018; Urbar-Ulloa et al. 2019; Essen et al. 2020). As such, RiAGLs could contribute to the dynamic nature of the fungal wall by responding to environmental and biotic stresses, or in cell identity (Balestrini and Bonfante 2014; Patel and Free 2019). To our knowledge, apart from RiAGLs, no other GPI-anchored proteins have been described in AMF, although they are well represented in other fungal species (Eisenhaber et al. 2004; Urbar-Ulloa et al. 2019).

If AGLs contribute to the establishment and/or maintenance of plant-fungal symbioses, then orthologues of RiAGLs should exist in other AMF species. Orthologues of AGLs sequences were not found in other eukaryotic genomes including the seven fungal genomes that were available at the time of their discovery (Schultz and Harrison 2008). AGLs are distinct from plant AGPs because they contain a high proportion of glycine (G), in addition to proline (P) and alanine (A) (over 50% of these three amino acids). Two of the three AGL proteins contain short tandem repeats rich in P and G, features commonly found in structural proteins such as elastin and spiders’ silk (Rauscher et al. 2006; Oktaviani et al. 2018; Gaar et al. 2020). The P to G ratio in the tandem repeats of RiAGL1 and RiAGL3 (Schultz and Harrison 2008; Creasey et al. 2012) suggests they have the potential to reversibly change from elastic to amyloid (fibrillar/plaque-like) properties based on conditions (Rauscher et al. 2006).

AGLs are an example of intrinsically disordered proteins (IDPs), with low amino acid complexity and few hydrophobic residues, and are expected to exist as extended (non-globular) proteins providing additional amino acids for molecular interactions (Schlessinger et al. 2011; Szalkowski and Anisimova 2011; Uversky 2011; Forman-Kay and Mittag 2013). IDPs have a diverse range of roles as binding partners in signalling pathways and drivers and controllers of the formation of proteinaceous membrane-less organelles (Kulkarni and Uversky 2018; Pritišanac et al. 2020). There is evidence that IDPs were involved in the evolution of multicellularity and cell type specification (Light et al. 2013; Kulkarni and Uversky 2018; Niklas et al. 2018).

A key feature of IDPs is their structural flexibility and plasticity (Light et al. 2013; Kulkarni and Uversky 2018). To explore this, recombinant RiAGL1 and RiAGL3 and synthetic peptides derived from the PG-rich tandem repeats were evaluated. These experiments revealed that *R. irregularis* AGLs can form polyproline II (PPII) helices in vitro, sharing a key functional property of elastin and spiders’ silk (Creasey et al. 2012). Furthermore, the synthetic peptides ((APADGK)_5_ and (APKDG)_6_) based on the tandem repeats of RiAGL1 and RiAGL3, respectively, can self-assemble in vitro forming aggregates of different sizes, and both AGLs have faster assembly rates in low salt conditions compared to high salt conditions (Creasey et al. 2012).

The specific aims of this study were to (1) identify AGL genes in *R. irregularis* and *R. clarus* using a degenerate PCR approach with primers designed based on conserved ER/GPI signals (Schultz and Harrison 2008), (2) develop methods to identify AGLs from short-read RNA-sequence (RNAseq) datasets, and (3) characterise the diversity of AGLs in AMF. Aim 1 was completed (Wu 2013) before the first AMF genome was published (Tisserant et al. 2013) and was based on the hypothesis that plant EST libraries made from field grown plants would include AGL sequences from a range of AMF suitable for degenerate primer design*.* We present this Sanger sequencing data first as it revealed an unexpected level of diversity between the two *Rhizophagus* species. Aim 2 required the development of a targeted and flexible bioinformatics approach to overcome the difficulty of assembling sequences with tandem repeats from short-read sequence datasets (Johnson et al. 2017a). This research provides the critical first steps to finding and characterising highly variable cell surface proteins that are unique to Glomeromycotina.

## Materials and methods

### Identifying expressed sequence tags (ESTs) for designing degenerate primers

**Fig. 1:**
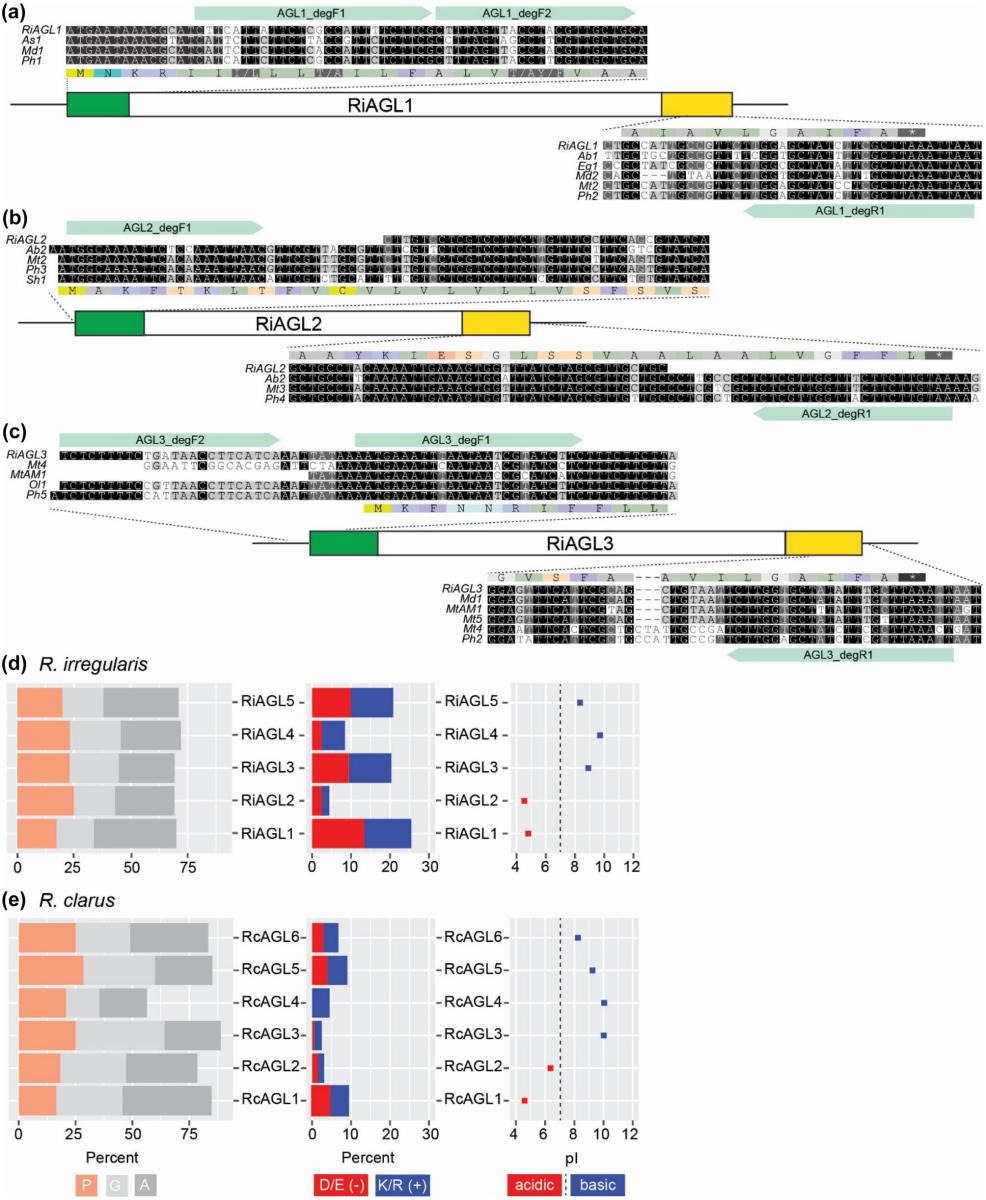
Cloning of AGLs from *R. clarus* and comparison to AGLs from *R. irregularis.* (**a**–**c)** Degenerate primers were used to amplify AGL gene sequences from *R. clarus* based on fungal AGL sequences found in plant root expressed sequence tag (EST) libraries. Schematic representation of proteins encoded by *R. irregularis RiAGL1* (**a**), *RiAGL2* (**b**), and *RiAGL3* (**c**). Boxes representing coding regions are approximately to scale. Sequence encoding the endoplasmic reticulum (ER) signal sequences (green boxes) and glycosylphosphatidyl inositol (GPI)-anchor signal sequences (yellow boxes) are highly conserved. Pale green arrows denote the regions corresponding to the degenerate primers (Table S2). See Table S1 for EST accession numbers and species abbreviations. (**d)** Amino acid biases and isoelectric points (pI) of mature AGLs proteins from *R. irregularis* and (**e)** *R. clarus* after cleavage of ER and GPI signal sequences

For aim 1, completed (Wu 2013) before the first AMF genome was published (Tisserant et al. 2013) (see “Introduction”), DNA sequences encoding the ER and GPI signal sequences of *RiAGL1*, *RiAGL2*, and *RiAGL3* (NCBI: EU931681, EU931682, and BI452321, respectively (Schultz and Harrison 2008)) were used as query sequences for tBLASTn (NCBI databases (nucleotide and ESTs), word size = 2, no filter, organism = land plants, performed in April 2013) (Table S1). The 5’ and 3’ sequences were aligned separately, and degenerate primers (Table S2) were designed to amplify AGLs from *R. irregularis* and *R. clarus* DNA (Fig. 1)*.*

### Genomic DNA extraction and PCR

Genomic DNA was extracted from approximately 100 mg of fungal spores and hyphae sampled from root organ cultures growing on *Daucus carota* clone DC2 hairy roots (Bécard and Piché 1992) using a Plant DNeasy Mini Kit (Qiagen). The fungal species were *R. irregularis*, DAOM 181,602 (St-Arnaud et al. 1996) = DAOM 197,198 (Stockinger et al. 2009), and *R. clarus*, Nicolson & Schenck (MUCL 46,238).

PCR reactions (100 μL) were performed with BioLine MyTaq Hot Start DNA polymerase using the supplied buffer. Degenerate primer concentrations were 2–4 × higher than usual (400–800 nM) due to degeneracy, with 1 ng genomic DNA. Cycling conditions: 95 °C, 3 min, then 32 cycles, 95 °C, 15 s; 45 °C, 15 s and 72 °C, 10 s, finishing with 72 °C for 10 min. In some cases, touchdown PCR was used (Korbie and Mattick 2008). Initial phase: annealing temperature from 62 to 45 °C (over 15 cycles) was followed by 35 cycles (annealing at 45 °C). PCR products either were sequenced directly or cloned. Sequencing (both strands) was performed by the Australian Genome Research Facility, Adelaide (AGRF). Sequences were analysed in Geneious 8.1.9 (http://www.geneious.com).

### BLAST searches of *R. irregularis* and *R. clarus* databases

To determine if AGL genes are correctly annotated in AMF genomes, BLASTp and BLASTn searches were performed at https://blast.ncbi.nlm.nih.gov/Blast.cgi as follows: BLASTn (NCBI whole-genome shotgun contigs database, word size = 7, no filter) and BLASTp (non-redundant proteins (nr) database, word size = 2, no filter), organism restricted to either *R. irregularis* or *R. clarus*. The query sequences were the partial *RiAGL* (Fig. S1a) and *RcAGL* (Fig. S1b) DNA and encoded protein sequences obtained by degenerate primer PCR.

### Characterisation of full-length AGL DNA and protein sequences

DNA sequences were translated, and encoded proteins analysed for signal sequences. ER signal sequences were predicted using http://www.cbs.dtu.dk/services/SignalP-4.1/, selecting “input sequences do not include TM regions” (Nielsen 2017). GPI-anchor signal sequences were predicted using fungal (http://mendel.imp.ac.at/gpi/fungi_server.html) and plant (http://mendel.imp.ac.at/gpi/fungi_server.html) predictors (Eisenhaber et al. 2003, 2004). Mature AGL proteins, after cleavage of signal sequences, were analysed using Biopython (ProtParam, ProteinAnalysis), for % amino acid (get_amino_acids_percent), molecular_weight, and isoelectric_point (Cock et al. 2009). Tandem repeats were identified using T-REKS (Jorda and Kajava 2009) (http://bioinfo.montp.cnrs.fr/?r=t-reks/) with “filter the overlapping repeats” (off), and percentage of similarity (Psim) = 0.7 and 1.0 for DNA and mature proteins, respectively. DNA alignments identified by T-REKS were imported into Geneious (as ClustalW alignments) for translation and highlighting. Codon usage was performed using http://www.bioinformatics.org/sms2/codon_usage.html.

### Phylogenetic analysis

Phylogenetic analyses were performed on a desktop computer using MEGA X (Kumar et al. 2018). Nucleotide sequences were aligned using Muscle with default settings, then the best model found for maximum likelihood. Percent nucleotide identity (%ID) between pairs of putative orthologues from *R. irregularis* and *R. clarus*, based on intron and exon boundaries and signal sequence cleavages, was performed in Geneious.

### Bioinformatic workflow to identify P- and G-rich proteins in AMF transcriptomes

Method 1 (Oases-mirabait-mira) involved a modified multiple *k-*mer assembly approach as used for plant hydroxyproline-rich glycoproteins (HRGPs) (Johnson et al. 2017a). For this workflow, see Fig. 5a. Short-read RNA datasets (sequence read archives (SRA)) were downloaded, trimmed, and error corrected using QUorUM (QUality Optimized Reads from the University of Maryland (Marçais et al. 2015)), then assembled using Oases (0.2.8, (Schulz et al. 2012)), one assembly for each of four different *k*-mers (*k* = 39, 49, 59, and 69, hereafter *k*39, *k*49, *k*59, *k*69). For analysis, we selected the eight transcriptomics datasets, derived from fungal spores, see Beaudet et al. (2018), as they covered a range of species within subphylum Glomeromycotina (species abbreviation, dataset (SRA), for *Funneliformis mosseae* (Fumos, SRR5279405), *Claroideoglomus claroideum* (Clcla, SRR5279407), *Diversispora versiformis* (Diver, SRR5279417), *Acaulospora morrowiae* (Acmor, SRR5279419), *Scutellospora calospora* (Sccal, SRR5279415), *Racocetra castanea* (Racas, SRR5279413), *Ambispora leptoticha* (Amlep, SRR5279409), and *Paraglomus brasilianume* (Pabra, SRR5279411). For each Oases assembly, the transcript.fa file was imported into Geneious, and a BLAST database created. Candidate AGL sequences were identified by BLASTn searches (ws7, e-1, gap cost (2 2)), using RiAGLs (or once identified, AGLs from a closely related species). The *k*49 assembly was analysed first (usually the “top” three loci, smallest e-values) with promising sequences, translated, and evaluated for signal sequences. If sequences were not full length (as assessed by absence of ER signal sequences or partial GPI signals) or were out of frame, the other three *k*-mer assemblies were queried to find the best sequence(s). BLASTn was performed using each new AGL sequence as a query sequence, up to a maximum of 10 sequences (only two species exceeded 10 putative AGLs). For each dataset, a list of AGL coding sequences (CDS) was assembled, with an additional 20 nucleotides each of 5’ and 3’ untranslated regions (UTRs), and used as “bait” sequences to identify matching short-reads from the raw (untrimmed) SRA using mirabait (mira 4.0.2) with default parameters (*k*-mer-length = 31 and *n* = 1, minimum number of *k*-mers needed) (Chevreux et al. 2004), run on a high performance computing facility at the University of Adelaide. Matching sequences were assembled using the paired reads option of mira using the plugin module in Geneious (mira de novo assembler, Version: 1.1.1 (Authors: Biomatters Ltd)) and used as a BLAST database. The Oases contig (CDS only) was used as query for BLASTn (as above). The best contig from Oases and mira was used as the final sequence for each species.

Where no AGLs were identified, datasets were assessed using FastQC (version 0.11.7) (Brown et al. 2017) and datasets ignored if either paired read scored orange or red (i.e., FastQC’s “traffic light” classification) for “per base sequence quality.” High-quality datasets (green) were analysed using method 2, to find one or two candidate proteins rich in either P, G, or both, in a non-exhaustive manner. Experience with other datasets suggested that once we had found one or two sequences, these were usually sufficient to find the remaining AGL sequences (data not shown). Method 2: Open reading frames (all 6 frames) were identified from Oases *k*49 and *k*59 assemblies using getORF (EMBOSS toolkit, http://emboss.sourceforge.net/). ORFs with PG% > 20% were identified using a modified Perl script (allowing for absence of an N-terminal methionine) (Schultz et al. 2002) (for this workflow, see Fig. 5a). ORFs encoding an ER signal peptide were manually inspected in Microsoft Word©, highlighting P and G residues to identify candidate AGLs. Transcripts encoding candidate proteins were used as query sequences in method 1.

### Comparison of G and P content in tandem repeats

Final mature AGL proteins from each species were analysed for the presence of tandem repeats (as above). The P and G percent of each repeat was calculated and graphed with other P- and/or G-rich proteins with known elastic or amyloid properties (Rauscher et al. 2006).

## Results

### Plant root EST libraries provide AGL sequences from unknown AMF species

Candidate AGL sequences from AMF species were identified from land plant EST databases prior to the release of the first AMF genome (Tisserant et al. 2013) (see aim 1, “Introduction”). Most ESTs were from eight libraries generated from root tissue of plants either grown in the field or inoculated growth media (Table S1). DNA sequence alignment suggested that putative orthologues of *RiAGLs* had been identified from different AMF species. For example, three ESTs aligned to the 5’ end of *RiAGL1*: EH047300 (As1, from a soil-grown root library from wild peanut, *Arachis stenosperma*), GO528602 (Md1, from a root library from apple, *Malus domestica*), and FN040184 (Ph1, from a root library of *Petunia hybrida* colonised by *Rhizophagus irregularis* isolate MUCL 43,204) and five ESTs aligned to the 3’ end of *RiAGL1*: GR364405 (Ab1, from a root library of bearded oat, *Avena barbata*), EL692929 (Eg1, from a root library of date palm, *Elaeis guineensis*), GO564967 (Md2, another EST from the apple root library), AL388047 (Mt2, from a root library of *Medicago truncatula* colonised by *Rhizophagus irregularis* isolate LPA8), and FN045610 (Ph2, another EST from the petunia library) (Fig. 1a). The other EST libraries that contained AGL-like sequences included a library from soil-grown roots of *Oryza longistaminata* (Ol) and field-grown roots of sugarcane, *Saccharum* hybrid (Sh) (Fig. 1b, c). Alignments revealed high % identity in regions coding for the signal sequences (Fig. 1) but the DNA sequence that encodes the mature proteins (after cleavage of the ER and GPI signal sequences, hereafter “mature coding sequence(s)”) was less conserved (data not shown). The low sequence conservation of the mature coding sequences is consistent with other IDPs that undergo rapid evolution that conserves amino acid composition rather than linear arrangement (Brown et al. 2011; van der Lee et al. 2014).

Degenerate primers were designed based on conserved regions near the start and end of each cDNA (Fig. 1 and Table S2). Genomic DNA was isolated from spores and hyphae of two AMF species grown in root organ cultures, *R. irregularis* and *R. clarus*, and was used for PCR*.* Sequencing of PCR products revealed four *R. irregularis* sequences, including one new partial gene sequence, *RiAGL4*, and five partial *R. clarus* genes, *RcAGL1* to *RcAGL5*. All the gene sequences included a single intron, and the encoded partial protein sequences of *R. clarus* AGLs were very different from the *R. irregularis* AGLs (Fig. S1a–d, and below). These partial genomic sequences allowed us confidently to search the completed genomes of *R. irregularis* and *R. clarus*.

### Full-length AGL tandem repeat genes are difficult to find in annotated genomes

Based on our experience with plant AGPs (Johnson et al. 2017a), we expected it would be difficult to find full-length AGL genes from annotated genomes. Therefore, we performed BLASTp and BLASTn searches and compared the top hit from three different *R. irregularis* annotated genomes (Tisserant et al. 2013; Lin et al. 2014; Maeda et al. 2018), one *R. clarus* annotated genome (Kobayashi et al. 2018), and a *R. clarus* transcriptome (Sędzielewska Toro and Brachmann 2016). BLASTp identified only two or three (of four) AGL proteins from *R. irregularis*, depending on the genome and two (of five) AGL proteins from *R. clarus* with coverage ranging from 33 to 100% and %ID from 54.9 to 100% (Fig. S2 and Table S3). Even for correctly identified proteins, the annotation was sometimes wrong such that ER and/or GPI-anchor signal sequences were not present. BLASTn produced more promising results based on coverage (84–100%) and %ID (89.1–100%) but in many cases, the annotated protein was different.

### Tandem repeat AGL genes are co-located in the genome

The *R. irregularis* PacBio assembly (Maeda et al. 2018) gave the best BLASTn results, although one gene, *RiAGL2*, was not correctly annotated (Fig. S2). Manual annotation of the matching sequences found four full-length sequences corresponding to the sequences amplified by PCR and one new gene for a total of five *RiAGLs* genes (Table S4 and Fig. S1e, f). Four of these genes are co-located in a 10.7 kb region of Unitig 292 (bases 527,748 to 538,492), whereas *RiAGL2* is found on Unitig 40. The co-located genes include the two sequences that encode the previously described zwitterionic / tandem repeat proteins RiAGL1 and RiAGL3 (Schultz and Harrison 2008; Creasey et al. 2012) and two new proteins RiAGL4 and RiAGL5.

For *R. clarus*, only two full-length genes, from a total of six genes identified with manual annotation, were correctly annotated and identified by BLASTn and BLASTp, *RcAGL1* and *RcAGL5* (Fig. S2, Tables S3 and S5). One new gene, *RcAGL6*, was identified from the genome sequence (Fig. S1g, h). As with *R. irregularis*, two scaffolds were identified, scaffold BEXD01004084 contained 5 AGL genes in a 16.6 kb region (bases 30,068 to 46,636) and BEXD01000960.1 contained *RcAGL2* (Table S5).

### Molecular characteristics of AGLs from *R. irregularis* and *R. clarus*

The diagnostic feature of mature AGLs (after cleavage of signal sequences) in *R. irregularis* and *R. clarus* is that they are rich in proline, glycine, and alanine (PGA), mostly > 70% PGA for RiAGLs, and even higher for RcAGLs at > 80% PGA, with the exception of RcAGL4 which is 58% PGA (Fig. 1d, e). RiAGLs differ notably from the mycorrhizal protein glomalin (Wright et al. 1998; Holátko et al. 2021; Irving et al. 2021) in that they are smaller (< 200 amino acids) compared to 590 amino acids for glomalin; highly disordered proteins (100% disorder compared to 10% disorder for glomalin); and RiAGLs generally have no sites for the addition of N-linked glycans (Table 1). In both *Rhizophagus* species, there are AGLs containing both acidic and basic amino acids, although RiAGL1, RiAGL3, and RiAGL5 have more charged residues (> 20%) than RcAGL1 and RcAGL5, which have ≤ 10% charged residues. Both AMF species have AGLs with acidic (RiAGL1, RiAGL2, and RcAGL1, at pI = 4.8, 4.6, and 4.7, respectively) and basic pI values (RiAGL3, RiAGL4, RiAGL5, RcAGL3, RcAGL4, RcAGL5, and RcAGL6, at pI = 8.9, 9.7, 8.1, 10, 10, 9.1, and 8.3, respectively) with only one AGL being close to neutral (RcAGL2, pI 6.2).

**Table 1:** Comparison of mycorrhizal RiAGLs/IDPs to glomalin, the heatshock protein 60 that is one component of “glomalin-related soil proteins (GRSP).” Two alternative terms have recently been suggested to replace GRSP: citrate extractable soil proteins (CESP) (Holátko et al. 2021) and high-temperature citrate extract (HTCE) (Irving et al. 2021) with the latter term acknowledging the presence of non-protein components including fatty acids, humic acid, chitin, and minerals

**Characteristic**	**RiAGLs**^**a**^	**Glomalin**
Length (aa)	102 (RiAGL4) to 181 (RiAGL1)	590 (encoded; transit peptide removed, 554)^b^
Localisation	Extracellular: membrane (GPI) and/or secreted	Mitochondria, some secreted?^b^
Solubility	Soluble	Insoluble
*N*-linked glycans^c^	No sites (RiAGL1, RiAGL2, RiAGL3, RiAGL4) One site (RiAGL5)	44 sites^d^
*O*-linked glycans^c^	RiAGL1 (5), RiAGL2 (23), RiAGL3 (11), RiAGL4 (16), RiAGL5 (10)	66 sites^c^
Secondary structure^e^	RiAGL1: α-helix (2%), β-sheet (0%), coil (97%) RiAGL2 to RiAGL5: coil (100%)	α-helix (50%), β-sheet (11%), coil (38%)
Solvent accessability^e^	RiAGL1 to RiAGL5: exposed (100%)	Exposed (35%), medium (31%), buried (32%)
Disorder^e^	RiAGL1 to RiAGL5: 100%	10%

^a^Native AGL proteins have not been purified. Characteristics are based on predictions using the gene-encoded mature proteins (with ER and GPI signal sequences removed). AGLs from *Rhizophagus irregularis* are used as examples. Glomalin is based on NCBI, ABE02805.1 (Gadkar and Rillig 2006)

^b^Glomalin is predicted to be a mitochondrial protein, based on TargetP, https://services.healthtech.dtu.dk/service.php?TargetP-2.0, with cleavage between amino acids (aa) 36 and 37 (RFY-AT). Experimental data suggests that some glomalin is secreted to membranes or walls of mycorrhizal hyphae (reviewed in Irving et al. 2021)

^c^Number of predicted sites for addition of *N*-linked or *O*-linked glycans (after removal of signal/targeting-sequence), based on GlycoMine, https://glycomine.erc.monash.edu/Lab/GlycoMine/, selecting *N*-linked and *O*-linked as appropriate (Li et al. 2015). For RiAGLs, the number of *O-*linked glycosylation sites is in parentheses after the protein name. As noted by Irving et al. (2021), glomalin is thought to be a mitochondrial protein and therefore would not undergo *N-* or *O-*linked glycosylation. Glomalin is frequently reported as a glycoprotein (Irving et al. 2021). The one scientific report of release of an *N-*linked oligosaccharide from glomalin could potentially be from a different protein(s) as the protein fraction used appeared to have undergone minimal purification (Wright et al. 1998)

^d^Three potential *N*-linked glycosylation sites are reported at positions 115, 438, and 465 (Irving et al. 2021). These sites rank (highest to lowest score, GlycoMine) 9, 2, and 17 respectively out of the 44 sites

^e^Secondary structure, solvent accessibility, and disorder predictions are from http://raptorx.uchicago.edu/StructurePropertyPred/predict/ (Wang et al. 2016)

### Selected AGLs from *R. irregularis* and *R. clarus* are putative orthologues but have different protein repeats

**Fig. 2:**
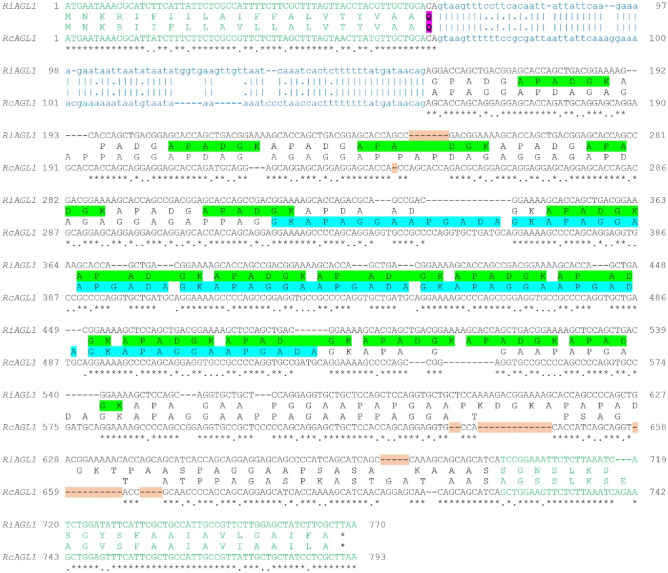
Pairwise alignment between putative orthologues *RiAGL1* and *RcAGL1.* Alignment of full-length PacBio gene sequences (Fig. S1e, g, respectively) was performed with EMBOSS Needle (DNA, https://www.ebi.ac.uk/Tools/psa/emboss_needle/). Alignments of the other pairs of putative orthologues are in Fig. S3. The translated protein sequences are superimposed, with the corresponding DNA sequence. Intron sequences in lower case blue text. Conserved nucleotides are indicated by * (coding) and | (intron) and semi-conserved bases (.). The sequences encoding the cleaved portions of the N-terminal ER and C-terminal GPI-anchor signals are in green text. The N-terminal Q (glutamine) residue of the predicted mature proteins is highlighted (pink). Gaps in the coding sequences that generate frameshifts (not multiples of three) are shaded light red. Repeat motifs are highlighted (APADGK of RiAGL1 (green); GKAPAGGAAPGADA of RcAGL1 (blue))

**Fig. 3:**
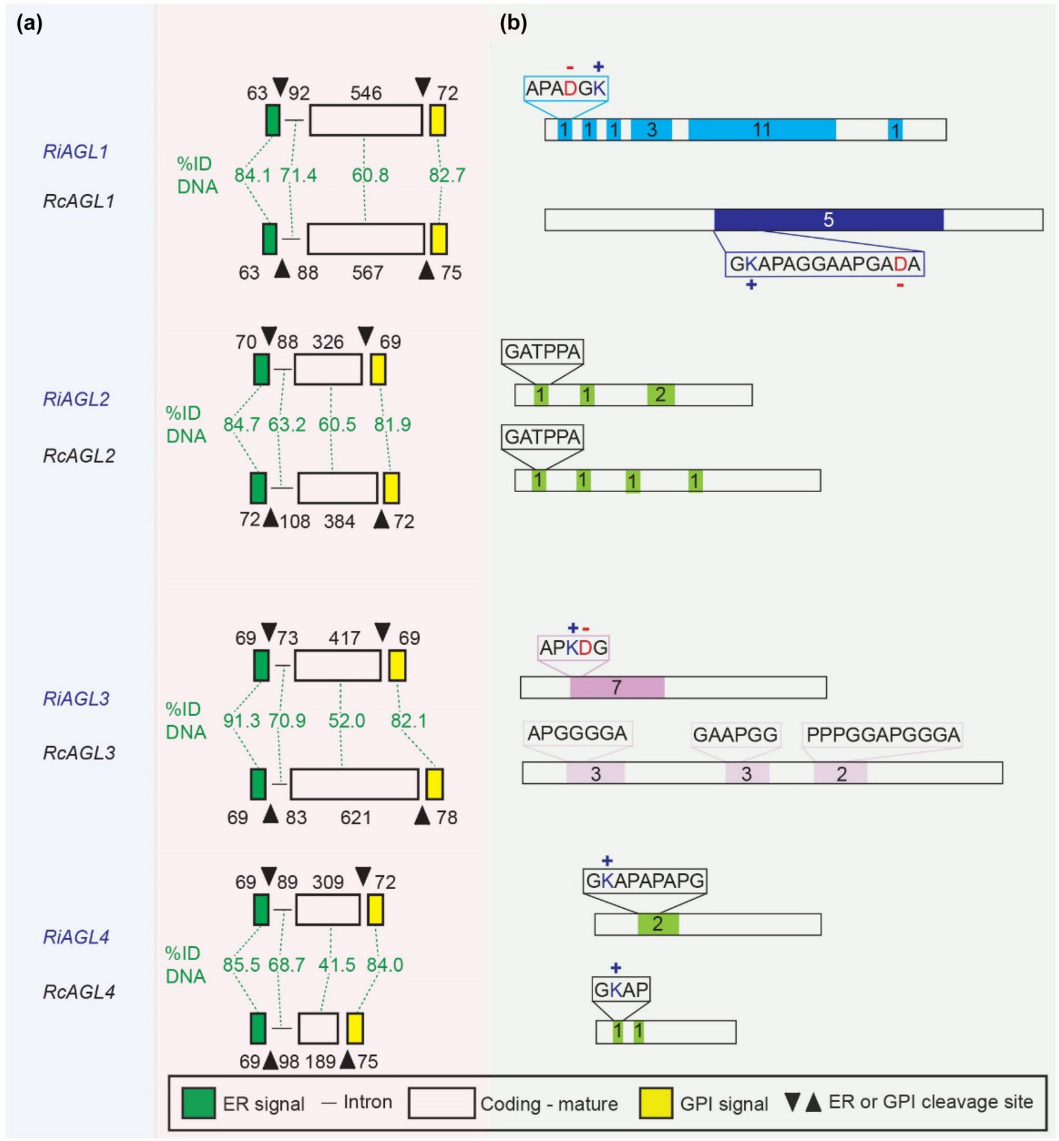
Comparison of % DNA identity by region and tandem repeat analysis of *AGL1* to *AGL4* from *R. irregularis* (Ri) and *R. clarus* (Rc). (**a)** Endoplasmic reticulum (ER) and glycosylphosphatidyl inositol (GPI)-anchor signal sequences have highest DNA identity (%) (82.7–91.3%), and the coding sequences of the mature protein are lower (41.5–60.8%). Intron sequences have intermediate identity (63.2–71.4%). DNA sequences used were the manually annotated PacBio sequences (Fig. S1e, g). Phylogenetic analysis of only the well conserved DNA coding sequences (at the 5’ and 3’ ends of the genes) suggests that the two *AGL1* sequences and the two *AGL3* sequences are orthologous (Fig. S3a, b). *RiAGL2* and *RcAGL2* genes were excluded from the phylogenetic analysis as they did not align well with the other AGLs (data not shown). However, it is likely that they are orthologues based on their relatively high % DNA identity (67.3%) compared to the other two pairs of orthologues, *AGL1* (65.7%) and *AGL3* (60.1%) (Fig. S4d), and the similarity of the predicted start of the mature proteins (after ER signal cleavage) including the absence of N-terminal glutamine as found in the other tandemly located AGLs in the two species. *RiAGL4* is compared to *RcAGL4* as they share the highest % ID for the full gene sequence compared to *RcAGL5 and RcAGL6* (Fig. S4d). (**b)** Zwitterionic repeats of RiAGL1 and RiAGL3 are absent (RcAGL2 to 4) or reduced (RcAGL1) in AGLs of *R. clarus*. The number of repeats is indicated in the coloured boxes

Pairwise DNA sequence alignments between *RiAGL1* and *RcAGL1* (Fig. 2), *RiAGL2* and *RcAGL2* (Fig. S3a), and *RiAGL3* and *RcAGL3* (Fig. S3b) suggest that these pairs of genes may be orthologous even though their mature coding sequences are very different. In all three pairwise alignments, there are gaps that are not multiples of three (potential codons), creating frameshifts that result in the different encoded proteins (Fig. 2 and Fig. S3). *Rhizophagus AGL* genes all have a single conserved intron positioned adjacent the cleavage site of the ER signal sequence. Despite conservation of gene structure, there is low similarity between putative orthologues in the mature coding region (41.5–60.8%), with higher sequence conservation in ER and GPI signal sequences (82.7–91.3%), with intron sequences (63.2–71.4%) more conserved than mature coding sequences (Fig. 3a). Protein identity in the mature proteins between RiAGL1 to 4 and RcAGL1 to 4 is even lower than DNA identity at 52.2%, 55.4%, 36.9%, and 29.8%, respectively. Another notable difference is that the zwitterionic repeats of RiAGL1 and RiAGL3 are either reduced and altered (RcAGL1) or absent (RcAGL3) from the *R. clarus* putative orthologues (Fig. 3b).

**Fig. 4:**
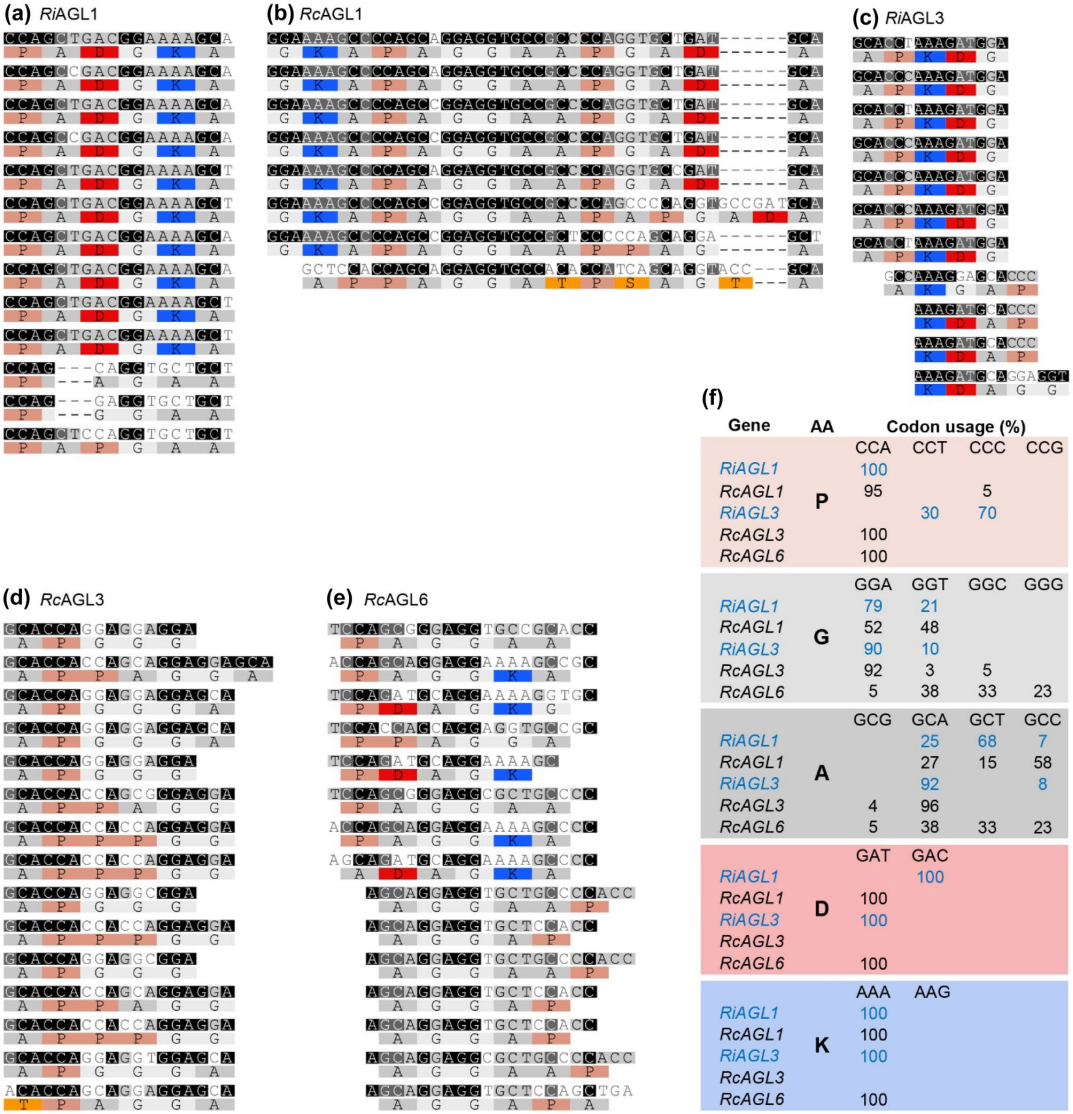
Codon usage within AGL repeats shows limited use of codons within the tandem repeats. DNA alignments of tandem repeats regions of *RiAGL1* (**a**), *RcAGL1* (**b**), *RiAGL3* (**c**), *RcAGL3* (**d**), and *RcAGL6* (**e**) were identified using (T-REKS, Psim = 0.7). The codon usages for P, G, A, D, and K in the repeats shown in (**a)** to (**e)** are summarised in (**f)**. The results for each T-REKS alignment are as follows: RiAGL1 (Psim:0.86; from 277 to 504; region length:228), RcAGL1 (Psim:0.888; from 235 to 576; region length:342), RiAGL3 (Psim:0.800; from 133 to 291; region length:159), RcAGL3 (Psim:0.84; from 313 to 573; region length:261), and RcAGL6 (Psim:0.75; from 111 to 413; region length:303). Psim = probability of similarity, the range indicates the region of CDS sequence aligned, and region length (in nucleotides (nt)). Different Psim setting for DNA (to allow for degeneracy) and protein (Psim = 1.0) resulted in some different repeat lengths and/or sequence compared to Fig. 3b

The tandem repeat finder, T-REKS (Jorda and Kajava 2009), was used to find repeats in the encoded proteins and DNA (Fig. 4). At the protein level, RcAGL1 has five tandem repeats of the 14 amino acid residue sequence GKAPAGGAAPGADA, with only a single basic residue (K), and a single acidic residue (D), compared to the shorter six amino acid repeats of RiAGL1 with one K and one D. RcAGL3 has three different tandem repeats, each containing only PGA residues. No tandem repeats were found in RiAGL2, RcAGL2, or RcAGL4.

A maximum likelihood (ML) tree (Fig. S4a) was generated from a multiple sequence alignment of the conserved exon sequences of *RiAGL1*, *RiAGL3*, *RiAGL4*, *RiAGL5*, *RcAGL1*, *RcAGL3*, *RcAGL4*, *RcAGL5*, and *RcAGL6* (Fig. S4b)*.* The conserved region is relatively short (164 nucleotides (nt)), comprising all of exon 1 sequence (65–71 nt, encoding the ER signal and a few additional amino acids), the first nine nt of exon 2, and the final 81–84 nt of exon 2 (encoding some of the mature C-terminus and all the GPI signal). In the excluded region, only 57 out of 790 (7.2%) aligned bases are 100% identical in all 9 sequences, making most of the mature coding sequence unsuitable for phylogenetic analysis due to the large number of gaps (Fig. S4c) (Dwivedi and Gadagkar 2009). *RiAGL2* and *RcAGL2* sequences were excluded from the alignment because of low sequence identity with the other AGLs (data not shown). Phylogenetic analysis supports orthology of *AGL1* and *AGL3* genes from *R. irregularis* and *R. clarus* based on strong bootstrap values (≥ 90). We suggest that *RiAGL2* and *RcAGL2* are likely orthologues based on their relatively high overall % DNA identity (67.3%) which is higher than the other two pairs of orthologues, AGL1 (65.7%) and AGL3 (60.1%) (Fig. S4d). This will require future validation when additional AGL2-like sequences become available.

### Analysis of codon bias is consistent with evolution of tandem repeats by localised duplication

Alignment of the DNA sequences encoding the tandem repeats within each AGL gene suggests that they evolved by tandem duplication of DNA repeats as shown for other tandem repeat proteins (Brown et al. 2011; van der Lee et al. 2014). The DNA-based alignments highlight restricted codon usage for most amino acids (Fig. 4). For example, the CCA codon for P is used 100% and 95% in the repeated regions of *RiAGL1* and *RcAGL1*, respectively. The GAC codon for D is used 100% in *RiAGL1* whereas the alternative codon GAT is used 100% in *RcAGL1* (Fig. 4f). K, in these selected tandem repeats, is always encoded by AAA.

### Pipeline for identifying P and G containing IDPs from short-read RNAseq datasets

**Fig. 5:**
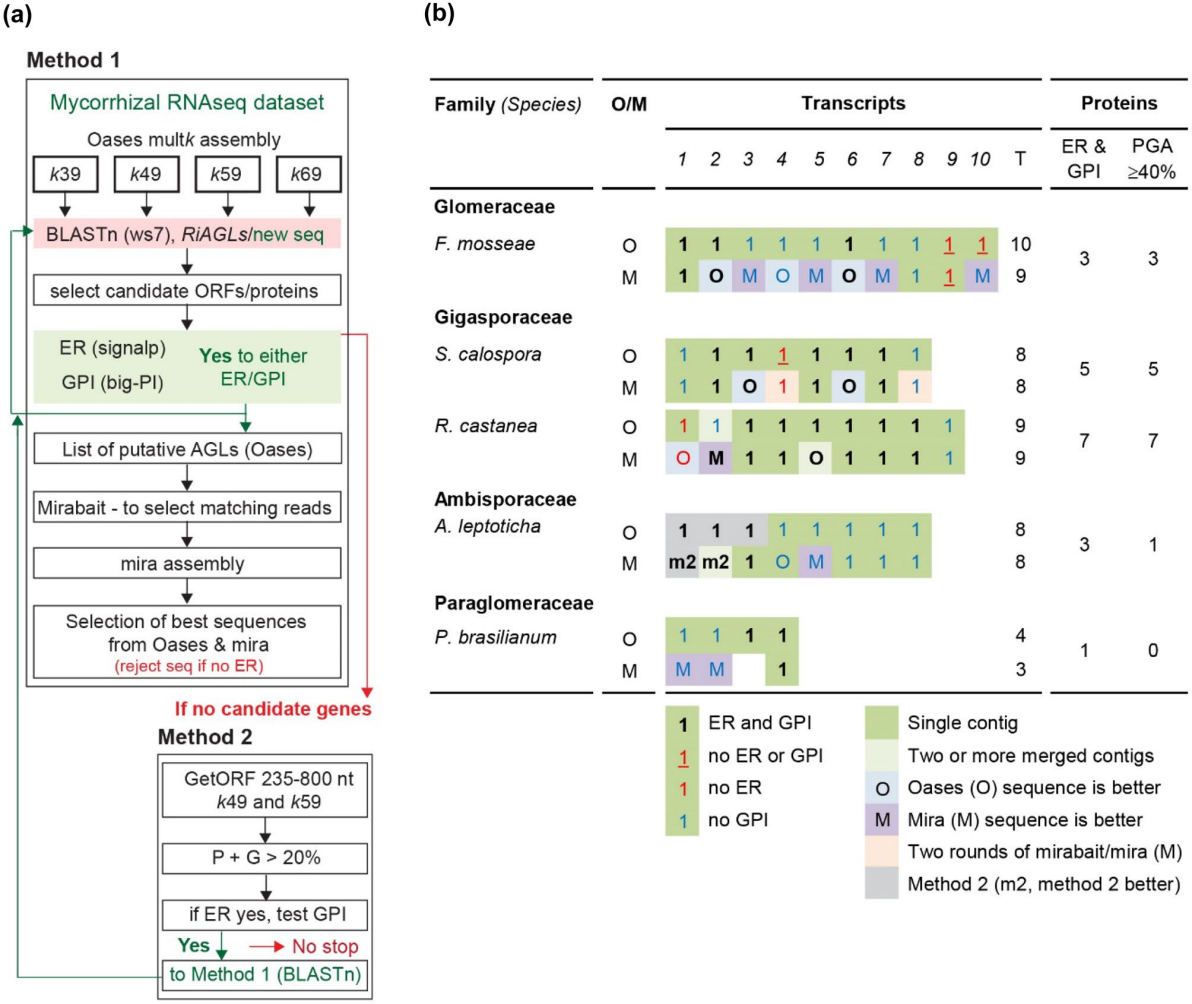
Pipeline for finding intrinsically disordered AGL proteins (**a**) and a summary of the outcomes for five species of arbuscular mycorrhizal fungi representing four taxonomic families (**b**). (**a)** Method 1, Oases-mirabait-mira, consists of three key steps. Step 1, Oases assembly with four different *k-*mers (*k*39, *k*49, *k*59, and *k*69) to find candidate AGLs. Candidate sequences, identified by BLASTn, are checked for the presence of endoplasmic reticulum (ER) and/or glycosylphosphatidyl inositol (GPI) signal sequences. Step 2, mirabait is used to extract matching sequences from the raw sequence read archive (SRA) datasets, and data assembled with mira. Matching contigs are identified with BLASTn and in the final step, step 3, the best sequences from Oases and mirabait/bait are retained for characterisation (Fig. S1i–r). Method 2, referred to as 20% PG, is used when no candidates are found with method 1, as a non-exhaustive way to identify candidate sequences for method 1. See “Materials and methods” for details. (**b)** Mirabait followed by mira improves identification and assembly of selected AGL sequences. For each AGL, identified by Oases (step 1) or mirabait/mira (step 2), boxes are colour coded/with different coloured text. Bold black text indicates that the encoded protein is predicted to have both ER and GPI-anchor signal sequences, plain red text (no ER signal), blue text (no GPI signal), and red underlined text (no ER or GPI signal). Coloured boxes are shaded green (to indicate full-length sequence, with a start and stop codon, in a single contig) and pale green (if full-length sequence comes from two or more contigs). For the final “best” sequences, characteristics were assessed for the mature proteins (after cleavage of ER and GPI signals) (see sequences (Fig. S1) and analyses (Table S6)). Aggregate number of sequences with the following characteristics is reported: presence of both ER and GPI signals and PGA ≥ 40%. Transcript numbers are arbitrary and do not imply orthology with *R. irregularis* sequences. Sequences with no ER signal after OASES and mirabait/mira were eliminated from further analysis as they are not secreted (e.g., Fumos_AGL9), unless they are partial sequences and have high sequence identity to an AGL with an ER signal sequence (e.g., Sccal_AGL4). A full-length version of Sccal_AGL4 was found by searching the NCBI_transcriptome shotgun assembly data (see Results) (Fig. S1)

Knowledge that AGLs in two *Rhizophagus* species have low sequence identity, except for ER and GPI signal sequences (Fig. 3), suggests that new approaches are needed to determine if all AMF families contain small families of PG-rich IDPs (see aim 2, “Introduction”). Therefore, we developed two bioinformatics approaches (Fig. 5a): method 1 (Oases-mirabait-mira) started with multiple *k*-mer assemblies as used to identify HRGPs from plants (Johnson et al. 2017a). Here we used Oases to assemble transcriptomes with four different *k-*mers (*k*39, *k*49, *k*59, and *k*69), then BLASTn to identify potential AGL contigs. Predicted proteins were evaluated for the presence of ER and/or GPI signal sequences, and sequences were retained if they had at least one signal sequence or were similar to a sequence that had one or more signal sequences. Sequences with no ER signal were ignored because they would not be secreted proteins. Retained sequences were used as bait sequences for mirabait (Chevreux et al. 2004) to identify “matching” reads in the original (untrimmed and unedited) datasets. Matching reads were assembled with mira, which takes into account read quality throughout the assembly and trimming process. Initially, we processed seven of eight datasets from Beaudet et al. (2018) as currently these are the only plant-free AMF data that include species from the ancestral families Ambisporaceae and Paraglomeraceae, giving in total six different taxonomic families, complementing existing data on Glomeraceae. Three datasets were eliminated due to poor “per base quality scores” (*C. claroideum*, *D. versiformis*, and *A. morrowiae*, data not shown). Three datasets gave useful results with method 1 (*F. mosseae*, *R. castanea*, and *S. calospora*, Fig. 5b).

**Fig. 6:**
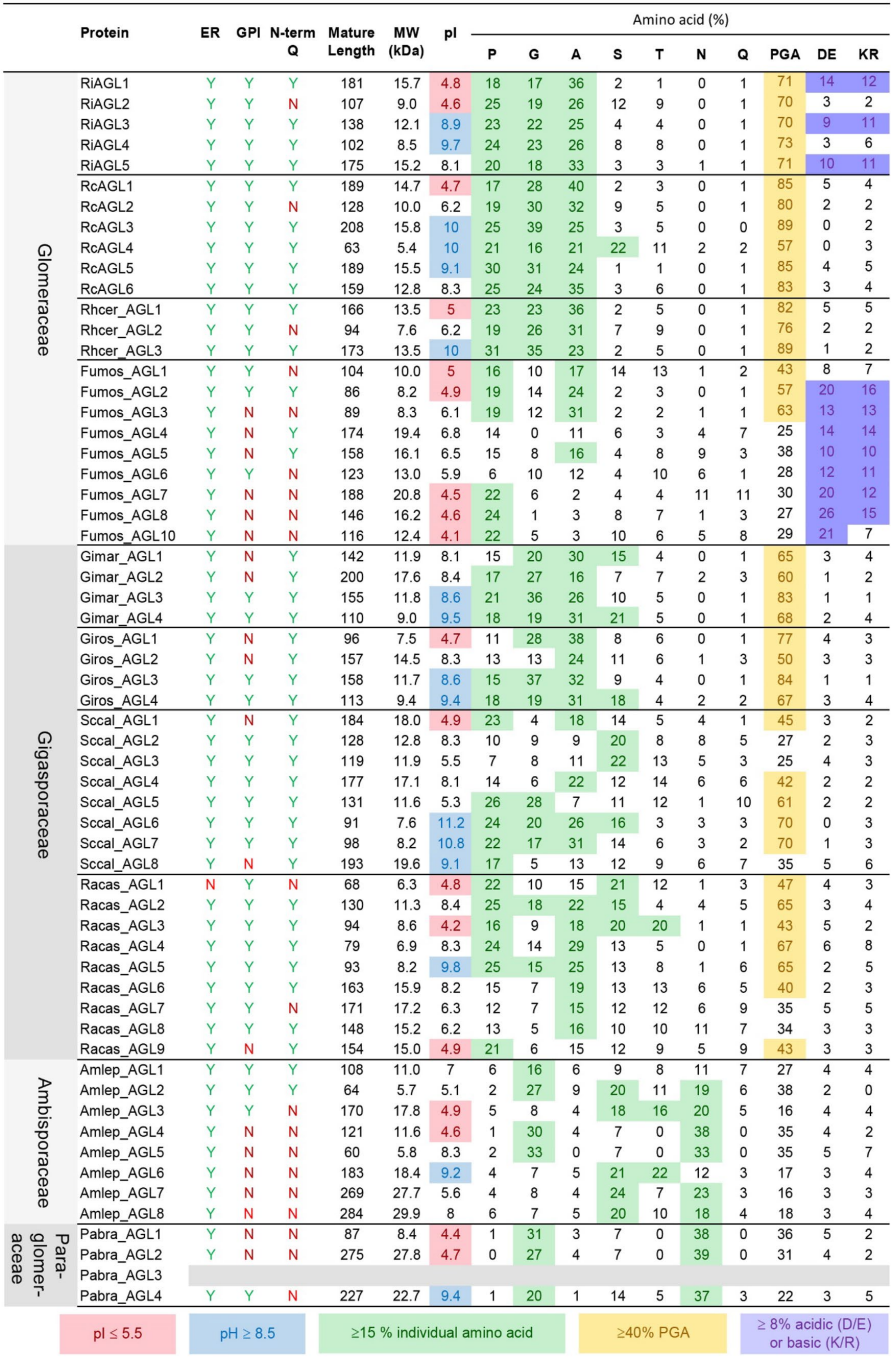
High diversity both with and between arbuscular mycorrhizal fungi species for secreted AGL/IDPs. Mature proteins were obtained after removal of predicted N-terminal endoplasmic reticulum (ER) and C-terminal glycosylphosphatidyl inositol (GPI)-anchor signal sequences (Fig. S1). Many of the predicted mature proteins start with an N-terminal Q (glutamine), which may be important for protein stability (Johnson et al. 2017b). Highlighting is used to indicate the following properties: pI, strongly basic (blue) or acidic (red); individual amino acids that are present at ≥ 15% (green); aggregate PGA% ≥ 40% (yellow); and DE (acidic) ≥ 8% or KR (basic) ≥ 8% (purple). Values for the other amino acids are reported in Table S6

For *F. mosseae*, ten sequences were “retained,” but only three of them had both ER and GPI-anchor signal sequences. The contigs obtained after mirabait/mira were compared to the bait (Oases) sequences, and the best nine sequences were kept for characterisation (Fig. S1i, j). Key protein statistics are summarised in Fig. 6 (length, molecular weight (MW), isoelectric point (pI), presence or absence of N-terminal Q (after ER signal cleavage), percentage of key amino acids (P, G, A, S, T, N, and Q), and %PGA, %DE, and %KR), with full amino acid composition profiles in Table S6. Fumos_AGL9 was eliminated as it had no ER signal sequence. The sequences Fumos_AGL1, Fumos_AGL2, and Fumos_AGL6 are predicted to be GPI-anchored, all nine *F. mosseae* AGLs have relatively low %G (0 to 12%), and only three sequences had PGA% ≥ 40%. A contrasting feature of *F. mosseae* AGLs compared to *Rhizophagus* AGLs is that they all were acidic to neutral, with Fumos_AG4 having the highest pI, pI 6.8. Lastly, most were highly charged, generally zwitterionic, with E preferred over D, different from the *R. irregularis* AGLs.

The Oases-mirabait-mira method identified nine AGL sequences from *R. castanea* (family Gigasporaceae). *RiAGL1* to *RiAGL5* cDNA sequences were used as query sequences for BLASTn searches of the Oases *k*49 assembly. Three potential Racas_AGLs were found and these were then used as query sequences to find an additional six sequences (Figs. 5b and S1). Mira was able to resolve a suspected frameshift in the Oases contig for *Racas_AGL2*. Seven of the encoded proteins are predicted to be GPI-anchored, seven have %PGA ≥ 40%, and only one has ≥ 8% KR (basic residues) (Fig. 6). Most of the AGLs (7 of 9) from *R. castanea* have mature proteins (after cleavage signal sequences) that start with an N-terminal Q (glutamine), which may be important for protein stability (Johnson et al. 2017b). Similar results were obtained for *S. calospora* (family Gigasporaceae), with five GPI-anchored AGLs (out of 8) identified*.* With this dataset, a second round of mirabait/mira was performed, successfully increasing the length of partial sequences *Sccal_AGL4* and *Sccal_AGL8* (Fig. S1).

Method 2 (> 20% PG, Fig. 4a) was used to identify IDPs from *A. leptoticha* (family Ambisporaceae) and *P. brasillanum* (family Paraglomeraceae) as no candidate AGLs could be found with method 1, despite using a variety of AGLs from different species as query sequences. The second method identifies a few candidate proteins by translating all 6 frames of *k*49 and *k*59 assemblies, then selecting open reading frames (ORFs) that had > 20% PG. Once a few candidate proteins were identified that included an ER signal sequence, the coding sequence of the relevant contigs was used for BLASTn searches using method 1 (Oases-mirabait-mira). A total of three GPI-anchored proteins were identified in *A. leptoticha* (out of eight) and one GPI-anchored protein from *P. brasillanum* (out of three) (Fig. 6 and Fig. S1). The most notable feature of these proteins is that they contained low levels of P (1–6% for *A. leptoticha* and 0–1% for *P. brasillanum*), but high G and for most, high levels of N (asparagine, 12–38% for *A. leptoticha* and 33–39% for *P. brasillanum*).

### Finding AGLs in transcriptomes and genomes is possible for closely related species once “seed” AGLs are identified with the IDP pipeline

The successful Oases/mirabait/mira pipeline used to identify AGLs and other novel IDPs (Fig. 5) is difficult to automate and time consuming; therefore, identifying scenarios where it is not needed would be valuable. Based on our earlier findings (Figs. 1, S2 and S3), we predicted that BLASTn would be effective for finding *AGL* genes within any given taxonomic family and possibly taxonomic order provided that the parameters were modified appropriately to maximise coverage (alignment length as percentage of query length). First, we tested this hypothesis with the family Gigasporaceae in which we had identified *AGL* sequences from both *S. calospora* and *R. castanea.* We started with *G. margarita* for which both transcriptome and genome data are available (Salvioli et al. 2016; Venice et al. 2020). Transcriptome data allows for easier manual annotation than genomic data as it eliminates the need for intron prediction. A total of 19 *G. margarita* genes and transcripts were identified by BLASTn, manually annotated where needed, and classified into five classes: 1, AGL > 40% PGA; 2, 15–39.9% PGA; 3, chimeric with “insignificant” match to PF10342; 4, chimeric–other; and 5, no ER or GPI signal (Table S7). Of these, 4 are considered AGLs based on predicted proteins with ER signal sequences (2 also have GPI signals), > 40% PGA, and small size (≤ 200 aa) (Fig. 6). All 19 *Gimar* sequences were used to find putative orthologues from the annotated *G. rosea* genome (Morin et al. 2019), and three full-length AGLs and one partial AGL were identified (Table S7 and Fig. S1).

One notable difference between AGL genes from *G. margarita* and *G. rosea* compared to *Rhizophagus* AGL genes was the presence of two introns in some genes (3 of 4 from *G. margarita* and the two full-length AGLs from *G. rosea*) Table S7. The AGL genes from *R. irregularis* and *R. clarus* only have one intron (Fig. S1)*.*

We also tested BLASTn in family Glomeraceae using *RiAGLs* as query sequences to search the recently published *R. cerebriforme* (*Rhcer*) datasets (Morin et al. 2019). Three full-length AGLs were found, each with a single intron in the conserved position observed in the other *Rhizophagus* species (Fig. S1).

To further evaluate BLASTn using seed AGLs identified by the Oases/mira/mirabait pipeline, we searched the published Trinity contigs for three of the datasets used in our study, *S. calospora* and *R. castanea* (family Gigasporaceae), and *F. mosseae* (family Glomeraceae) (Beaudet et al. 2018). Only one search (out of 27) provided an improved, apparently full-length sequence, and that was for *Sccal_AGL4* (based on alignment to *Racas_AGL6)* (Table S8). Nine of the 27 searches produced a top hit sequence that was notably worse compared to the Oases/mirabait/mira approach, highlighting the necessity of this new pipeline and manual annotation for identification of AGLs (Table S8).

Finally, we tested whether BLASTn would work for finding AGL sequences in *Geosiphon pyriformis* using AGLs identified using the pipeline from ancestral AMF species *P. brasillanum* and *A. leptoticha* (Fig. S1). *Ge. pyriformis* is in the same taxonomic order (Archaeosporales) as *P. brasillanum* and *A. leptoticha*, and is the only known fungus to form an endosymbiosis with nitrogen-fixing bacteria. *Ge. pyriformis* may represent the ancestral state of AMF (Gehrig et al. 1996; Malar et al. 2021); therefore, it is of interest to know if GN-rich IDPs are found in this non-AMF endosymbiont.

Coding sequences of representative IDPs from *P. brasillanum* and *A. leptoticha*, and AGLs from *R. irregularis* were selected as query sequences to determine if GN-rich IDPs or PG-rich AGLs occurred in *Ge. pyriformis* or other groups of fungi (Mortierellomycotina, Mucoromycotina, Basidiomycota, and Ascomycota). The three *P. brasillanum* sequences, *Pabra_AGL1*, *Pabra_AGL2*, and *Pabra_AGL4* and *Amlep_AGL4*, all detected three full-length cDNAs, MH580277.1, MH580278.1, and MH580279.1 (and no other sequences), with *Pabra_AGL2* having the best coverage (91%) with 67.2% identity to MH580278.1 (Table S9), a protein annotated as RIC2 (“Repeat containing proteins In symbiosis with Cyanobacteria” (Hoffrichter 2018)).

No sequences encoding secreted proteins were identified by BLASTn in the other two subphylla (Mortierellomycotina and Mucoromycotina) of phylum Mucoromycota in either the non-redundant or transcript shotgun assembly (TSA) databases at NCBI (Table S9). Searches also were performed for Basidiomycota and Ascomycota, and no significant sequences were identified. A few secreted proteins were identified, but these only had short regions with amino acid biases (Table S9). These results suggest that AGLs and IDPs are found only in fungi of the Glomeromycotina.

### Most species of AMF have AGLs with distinct PG-rich, or G-rich, or P-rich tandem repeats

**Fig. 7:**
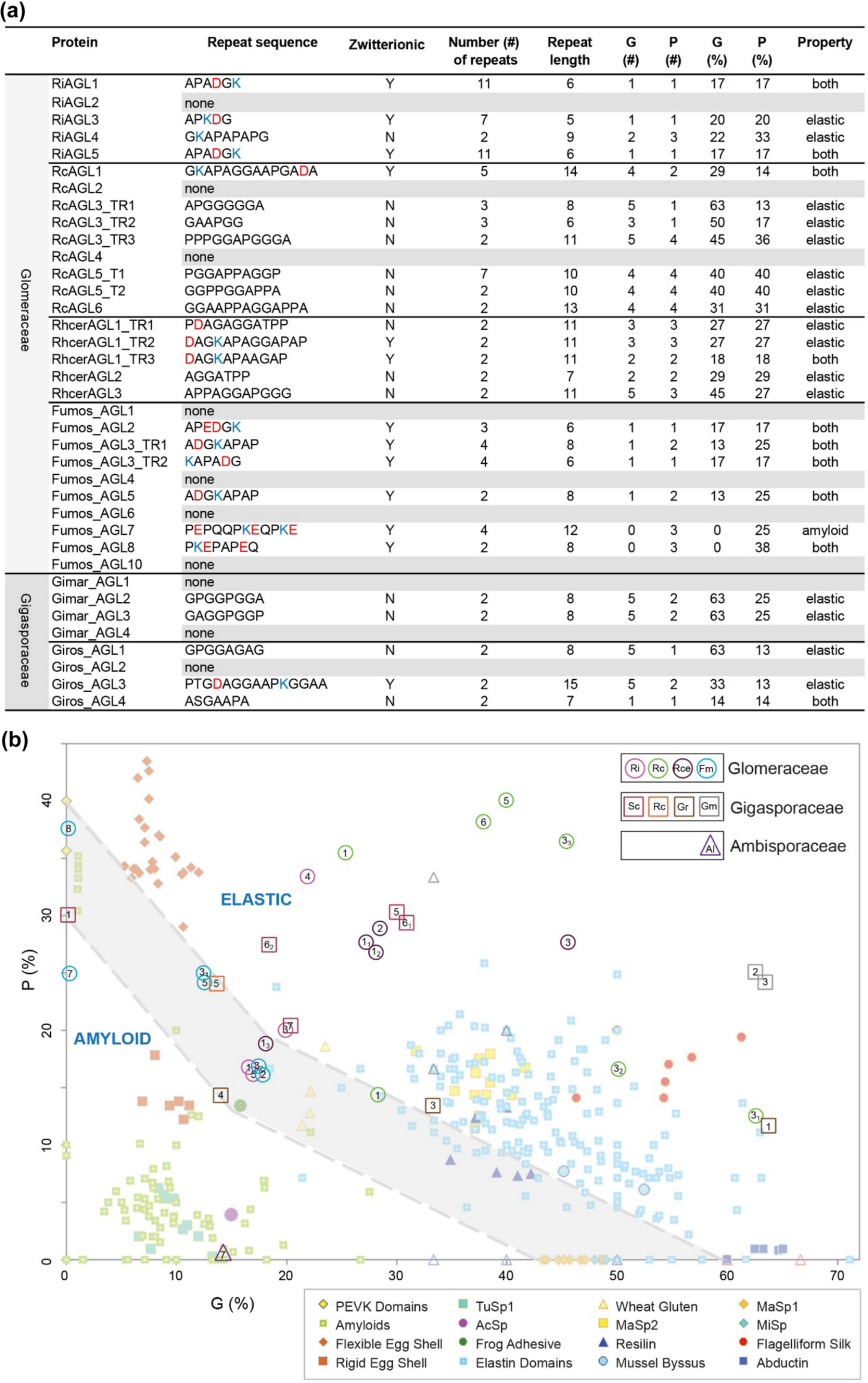
Most species of arbuscular mycorrhizal fungi (AMF) have AGLs or intrinsically disordered proteins (IDPs) with distinct PG-rich or G-rich or P-rich tandem repeats. (**a)** Subset of tandem repeats found in mature AGL proteins from four species from the family Glomeraceae, and two of four species from the family Gigasporaceae*.* All species are reported in Table S10. Repeats were identified using T-REKS (Psim = 1.0, filter overlapping repeats (off)) (Jorda and Kajava, 2009). The tandem repeat we found by eye for RiAGL2, (GATPPA)_2_ is not picked up at Psim = 1.0, but a variant including this sequence is reported at Psim = 0.9. Where different tandem repeats (TR) were found in one protein, they are indicated as TR1, TR2, and TR3 (e.g., RcAGL3). Repeats are classed as zwitterionic (Y, yes or N, no) if they have at least one positive and one negative charged amino acid. Property indicates the predicted amyloid or elastic property of the tandem repeat based on relative P and G content (Fig. 7b, based on Rauscher et al. 2006). (**b)** Proteins with high PG content generally have elastic properties, whereas proteins with low PG content are amyloid. Proteins that fall in the grey “intermediate” zone are predicted to have reversible characteristics, based on molecular dynamic simulations (Rauscher et al. 2006). Each AMF species with AGLs/IDPs containing tandem repeats is represented by a different coloured open shape as follows: family Glomeraceae (circle: Ri, *R. irregularis* (pink)); Rc, *R. clarus* (green); Rce, *R. cerebriforme* (Rhcer, dark purple); Fm, *F. mosseae* (Fumos, turquoise), Gigasporaceae (square: *S. calospora* (Sccal, dark red)), *R. castanea* (Racas, orange), *G. rosea* (Giros, brown), *G. margarita*, (Gimar, grey); and Ambisporaceae (triangle: *A. leptoticha* (Amlep, purple)). *P. brasilianum* IDPs contain imperfect tandem repeats, identified with Psim 0.8 and would all be amyloid due to absence of P (Table S10). The shape contains the protein (AGL) number, and if more than one repeat was identified (e.g., TR1, TR2, and TR3), a subscripted number is used for the different repeats (3_1_, 3_2_, 3_3_). If repeats have the same P% and G%, then one is offset, down and to the right, to allow the numbers to be seen (e.g., RiAGL1 and Fumos_AGL2). The following repeats have identical G% and P%: RiAGL1, RiAGL5, Fumos_AGL2, and Fumos_AGL3_TR2 (16.7% for both P and G); Fumos_AGL3_TR2, Fumos_AGL5, and Racas_AGL5 (12.5% and 25%, respectively); RiAGL3 and Sccal_AGL7 (20% and 20%, respectively); Sccal_AGL5 and Sccal_AGL6_TR1 (30% and 30%, respectively); TR1 and TR2 in RcAGL5 have the same G% and P% (40% for both G and P); RcAGL3_TR1 and Giros_AGL1 (62.5% and 12.5%, respectively); Rhcer_AGL1_TR1 and TR2 (27% for both G and P); and Gimar_AGL2 and Gimar_AGL3 (63% and 25%). The graph and legend (**b**) are modified with permission from Rauscher et al. (2006). Abbreviations from the legend are PEVK domains of titin (an elastin), tubulliform silk (TuSp1), aciniform silk (AcSp), major ampullate spindroin 2 (MaSp2), major ampullate spindroin 1 (MaSp1), and minor ampullate spindroin (MiSp). Open triangles (no text) are model proteins analysed by Rauscher et al. (2006)

A defining feature of AGLs is that they are IDPs that contain amino acids that promote disorder, P or G or both PG (Uversky 2011). It is not known if all AGLs contain tandem repeats. Unlike *R. irregularis*, where tandem repeats could be detected by eye (Schultz and Harrison 2008), we used T-REKS to search for tandem repeats in the new AGLs. Most species had at least one AGL with a tandem repeat (except *G. margarita* and *P. brasillanum*); however, outside of family Glomeraceae, the maximum number of repeats was two, and only *G. rosea* had a repeat that was zwitterionic (PTGDAGGAAPKGGAA) (Fig. 7a and Table S10). Because the relative proportion of P to G can change the propensity for amyloid to elastic properties, we graphed the %G to %P of the tandem repeats for each AGL and superimposed the data on a graph with known PG-rich proteins (Fig. 7b). Half of the tandem repeats (20 of 34) from AGLs occur in the “elastic” region, with only two tandem repeats occurring in the amyloid region (Fumos_AGL7 and Amlep_AGL7). There are 11 tandem repeats, from six species (families Glomeraceae and Gigasporaceae), that fall in the intermediate zone, hereafter referred to as interchangeable repeats. These repeats were found in AGLs from *R. irregularis* (RiAGL1, RiAGL5), *R. clarus* (RcAGL1), *F. mosseae* (Fumos_AGL2, Fumos_AGL3 (repeats 3_1_ and 3_2_), Fumos_AGL5, and Fumos_AGL8), *S. calospora* (Sccal_AGL1), *R. castanea* (Racas_AGL5), and *G. rosea* (Giros_AGL4).

## Discussion

This research provides an example of a small gene family of apparently species-specific proteins that could contribute to the functional diversity of AMF. These proteins appear to be restricted to subphylum Glomeromycotina based on current sequencing data. The AGL/IDP-finding bioinformatics pipeline developed allowed both targeted finding of transcripts encoding PG-rich AGLs proteins in species from family Glomeraceae and Gigasporaceae, and novel GN-rich IDPs in the ancestral AMF families Ambisporaceae and Paraglomeraceae. We propose the use of the general name IDP for proteins with low or no P but high G, as observed for *A. leptoticha* and *P. brasillanum* (Fig. 6), but retaining the name AGL, for AGP-like protein, for AMF proteins that contain high levels of P (Schultz and Harrison 2008). This study provides sequences for primer design for validation where annotation is uncertain, and tools for finding AGLs/IDPs in future genomes and transcriptomes. Below, we discuss the key similarities and differences among the proteins, their potential evolutionary relationships, and implications for functions based on the different amino acid biases, tandem repeats, and cell surface locations including GPI-anchoring.

### AGLs, IDPs and molecular property predictions

Within *Rhizophagus* species, AGLs differ in extent (and/or presence) of zwitterionic tandem repeats (compare RiAGL1 and RiAGL2), pI (compare RiAGL1 and RiAGL2), and size (Figs. 6 and 7. Only *Rhizophagus* species have exclusively PGA-rich AGLs based on the 15% level adopted as high for individual amino acids. All four species from family Gigasporaceae have some PGA-rich AGLs, but others with < 15% of at least one of P, G, or A. The other two species studied, *A. leptoticha* (Ambisporaceae) and *P. brasillanum* (Paraglomeraceae), all have IDPs with low P (≤ 6%) and both species have three proteins rich in G and N.

GN-rich IDPs similar to those of *A. leptoticha* (Ambisporaceae) and *P. brasillanum* (Paraglomeraceae) are also found in *Ge. pyriformis.* Our searches identified three proteins, GpRIC1, GpRIC2, and GpRIC3, that were identified initially as encoded proteins from short-read (Illumina) sequencing of extracted cDNA inserts from a *Ge. pyriformis* expression library (Hoffrichter 2018). Full-length cDNA clones were obtained by colony hybridisation and subsequently sequenced (Sanger). Interestingly, all three encoded GpRIC proteins include the di-motif KR suggesting they contain possible Kex2 cleavage sites (Hoffrichter 2018), which would result in short secreted proteins if cleaved as expected after the R (Le Marquer et al. 2019).

GN (and GQ)-rich proteins are associated with amyloid characteristics, in proteins with and without tandem repeats (Rauscher et al. 2006; Mier et al. 2020) (Fig. 7 and Table S10). Only one protein from *A. leptoticha* contains a tandem repeat, and this repeat is 14.3% G (0% P), making it one of only 2 repeat-containing AGLs/IDPs with predicted amyloid properties. The remaining repeats identified are predicted to impart elastic properties or are changeable between elastic and amyloid properties. It is proteins such as RiAGL1 (& 5), RcAGL1, Rhcer_AGL1, Fumos_AGL5 (&7), Giros_AGL4, and Sccal_AGL1 that have the clearest structure–function link to non-homologous proteins such as spiders’ silk and elastin (Rauscher et al. 2006; Creasey et al. 2012; Oktaviani et al. 2018).

The presence of AGLs with zwitterionic tandem repeats is most common in family Glomeraceae, ranging from one in *R. clarus* and *R. cerebriforme* and three in *R. irregularis* to six of 10 AGLs in *F. mosseae.* The zwitterionic nature of the PG-rich repeats potentially confers self-assembly, assisted by PPII helix formation, as previously demonstrated for RiAGL-derived peptides and recombinant proteins (Creasey et al. 2012; Yarawsky et al. 2017).

All species studied so far, with the exception of *F. mosseae*, had AGLs/IDPs that include proteins with both acidic and basic pIs. This potentially could broaden the pH range in which AGL/IDPs function within a species, such as in different soils or in different intraradical compartments. Recent studies show some eukaryotic IDPs, albeit with significantly larger, net opposite charges, are attracted at picomolar affinity as observed between histone H1 and its chaperone prothymosin-α (Borgia et al. 2018).

The high diversity within and among AMF species for AGL/IDPs is consistent with known IDPs where disordered proteins evolve under a regime of stabilising selection that preserves features important for function, but leads to a high diversity of protein sequences (Pritišanac et al. 2020).

### AGL/IDP pipeline, assembly methodology and manual annotation

The pipeline developed highlights the on-going challenge of finding and annotating IDPs/tandem repeat proteins de novo. A limitation of this pipeline is the need for manual annotation and analysis of BLAST alignments after assembly, of both the Oases and mira assemblies. We attempted to use GRABB (Brankovics et al. 2016) as a more iterative approach that incorporates mirabait/mira; however, the number of potential contigs increased too rapidly to be useful (data not shown). As observed for plant HRGPs (Johnson et al. 2017a), a multiple *k-*mer assembly approach was necessary for success of step 1 as no single *k*-mer was best at finding fungal AGLs/IDPs. The additional step of mirabait/mira improved the assembly of about half the 18 sequences where there were differences between the two assemblies (Fig. 5b). Now that additional AGLs/IDPs are known, it may be possible to start with step 2 (mirabait/mira) performing two rounds, one at low stringency (optimising the parameters of *k-*mer length and number of matches) followed by a second higher stringency round. Our experience with both AMF (this paper) and plant IDPs (Schultz et al. 2002; Johnson et al. 2017a) suggests that there is no single method that works well for all IDP gene families and there is a large amount of trial and error to find suitable methods for each gene family, especially when covering a broad taxonomic range.

The sequences obtained for *R. irregularis*, *R. clarus*, *G. margarita*, and *G. rosea* are reliable as they are based on multiple forms of data, multiple datasets, or where comparison to another species from the same genus improved annotation (Figs. 1, S3 and Table S7). Despite the limitations of “ultra-low” input RNAseq approaches (Beaudet et al. 2018), we obtained useful AGL/IDP sequences from two datasets in the family Gigasporaceae, and one dataset each from ancestral families Ambisporaceae and Paraglomeraceae. The most problematic dataset in terms of signal sequence prediction was *F. mosseae*, and this may be because of relatively low data quality based on a “BUSCO” benchmark with 180/290 (62.1%) of the core fungal sequences either fragmented or missing (Beaudet et al. 2018).

Three other datasets had relatively high numbers of missing or fragmented sequences: *A. leptoticha* (64.8%), *P. brasillanum* (61.4%), and *R. castanea* (56.6%). We recommend that the sequences from these species be validated when future datasets are available. *R. cerebriforme* genome data (Morin et al. 2019) may be incomplete, based on the relatively few AGLs identified (three) compared to five and six in the other two *Rhizophagus* species*.*

Where proteins are not predicted to be GPI-anchored, these could be true non-GPI-anchored proteins, or false negatives (due to issues with prediction of GPI-anchor cleavage or due to inappropriate sequence assembly). We speculate, based on experience with plant GPI-anchor predictions (Johnson et al. 2017a, b), that big-PI fungal predictor (Eisenhaber et al. 2004) and even big-PI plant predictor (Eisenhaber et al. 2003) work well for AMF, and therefore, AGL1 and AGL2 in *G. margarita* and *G. rosea* are likely not GPI-anchored, but that in the lower quality datasets (as noted above), the negative results may largely be due to partial sequences. Purification and analysis of proteins from *Gigaspora* species would be a good place to start to validate GPI-anchor predictions.

Annotation of AGL genes likely is hampered by the presence of one (Glomeraceae) or two introns (some Gigasporaceae) near the boundary between the signal sequences and the mature protein coding sequence. *R. irregularis* and *R. clarus AGLs* all had a single intron between the sequences coding the ER signal sequence and the start of the mature protein, leading to short first exons (e.g., *R. irregularis* (65–79 nt) (Figs. 1, 3 and S2)).

### Evolution of AGLs

Understanding the evolution of AGLs remains a challenge. Rapid evolution is common for proteins with tandem repeats where repeats can reduce in number, undergo changes including frameshifts, and then subsequently expand (Brown et al. 2011; van der Lee et al. 2014). Such changes are evident comparing the DNA and proteins of putative orthologues of AGL1 and AGL3 from *R. irregularis* and *R. clarus* (Fig. S3). Changes also could occur as a result of recombination (Chen et al. 2018a) and gene duplication. We found evidence for gene duplication of tandem repeat AGL genes (Tables S4 and S5) from the long-read PacBio data. Future availability of additional AMF genomes derived from long-read sequence data will provide improved understanding of whether tandem duplication of AGL genes exists outside of *Rhizophagus* spp. It is unlikely that synteny relationships will be evident except for closely related species, based on a “very high level of structural rearrangements” (Morin et al. 2019).

Robust phylogenetic analysis will likely remain a challenge into the future, and it may take tens of species per genus and many genera to obtain meaningful phylogenetic trees. We predict that even with such comprehensive data, it will be necessary to analyse separately the orthologous groups of AGLs (e.g., *RiAGL1*, *RcAGL1*, and new putative AGL1 orthologues), based on the loss of information introduced when non-homologous AGLs are compared (for example, compare Fig. 2 and Fig. S4c). Useful data are emerging for *R. irregularis* where there are now two studies providing the genome sequence of five different isolates (Chen et al. 2018b; Yildirir et al. in press). These data will enable the analysis of relatively recent evolutionary changes in AGL sequences, providing opportunities for comparison of the evolution of AGLs with other tandem repeat proteins in non-AMF species. Analysis of these new data may need to be supported by high-quality Sanger sequences for the tandem repeat regions to overcome uncertainty around assembly methods.

When AGLs were first described in *R. irregularis* and no similar genes were found in other fungi, one possible explanation was that they arose by horizontal gene transfer from an early plant (Schultz and Harrison 2008). Horizontal gene transfer recently has been shown to occur between *R. irregularis* and both plants and bacteria (Li et al. 2018); however, the low conservation of AGLs even within the same genus will make it difficult, if not impossible to prove such an origin, even if it did occur.

### Potential function of AGLs and future directions

**Fig. 8:**
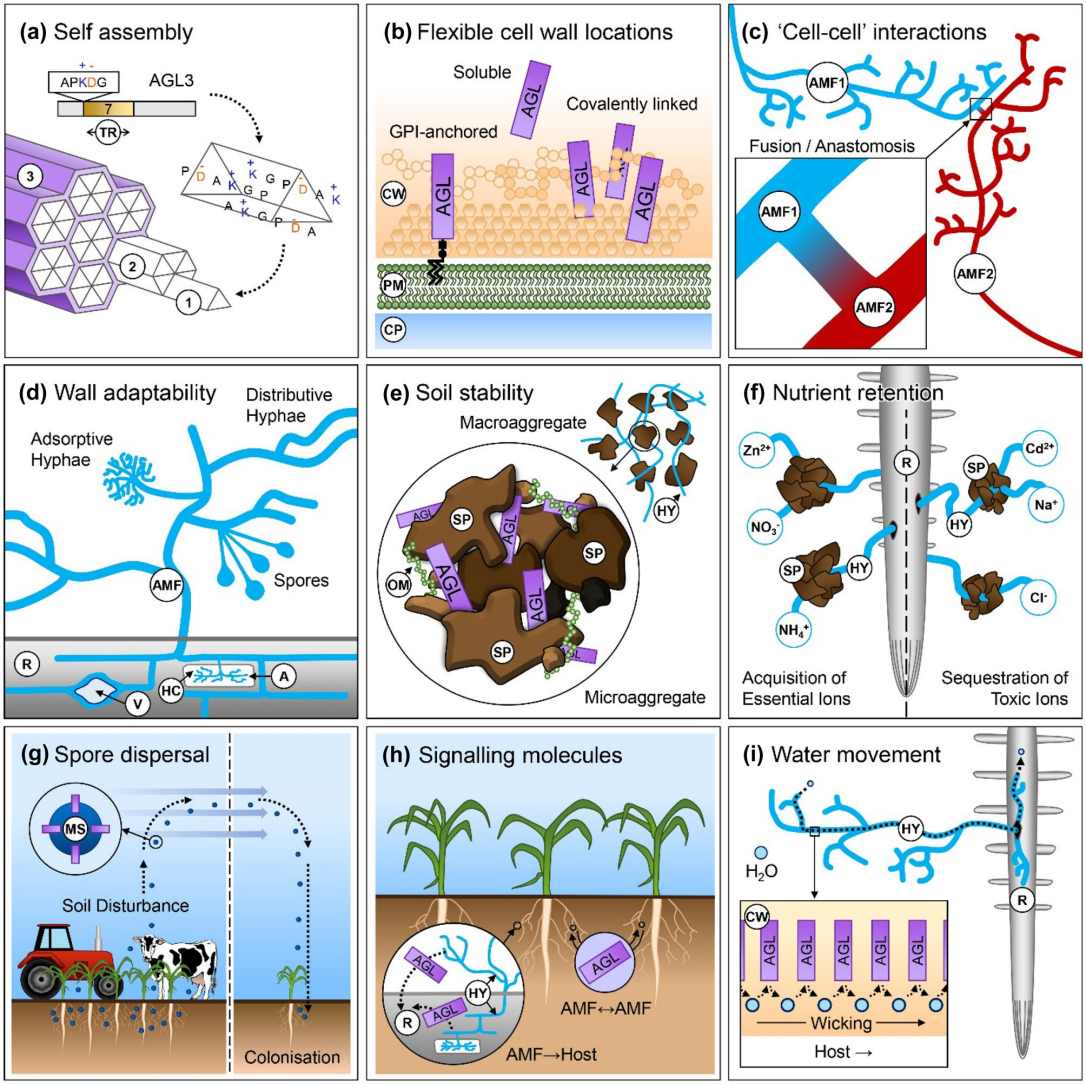
Model of possible AGL and intrinsically disordered protein (IDP) functions. (**a)** Self-assembly. AGLs may function as individual polypeptides (1), small multimers (2), and/or as larger complexes such as fibres (3). (**b)** Flexible cell wall locations. AGLs can potentially exist in any one, or all of the following extracellular locations due to the presence of a GPI-anchor: attached to the outer leaflet of the plasma membrane (PM), soluble (after cleavage of GPI-anchor), or potentially covalently linked to the cell wall (CW) (Essen et al. 2020). CP, cytoplasm. (**c)** “Cell–cell” interactions. AGLs could participate in cell–cell or cell-substrate adhesion (Essen et al. 2020) generally and/or more specifically for self-recognition as occurs during anastomoses in AMF (Chagnon et al. 2013). (**d)** Wall adaptability. AGLs could contribute to cell wall strength/flexibility/adaptability in soils and in different fungal structures within the root (R) (arbuscules (A), vesicles (V), intraradical hyphae). HC, host cell. (**e)** Soil stability. Formation and stabilisation of soil particles, as originally proposed for glomalin-related soil proteins (Rillig and Mummey 2006; Holátko et al. 2021). AGLs could stabilise microaggregates (< 250 μm) and macroaggregates (> 250 μm) (Rillig and Mummey 2006). HY, fungal hyphae; OM, organic matter; SP, soil particulate. (**f)** Nutrient retention. Capture and retention of micronutrients in soil (e.g., NH_4_^+^, NO_3_^−^, Ca^2+^, and Zn^2+^) (Cavagnaro et al. 2015) or amelioration of toxic ions (Na^+^, Cl^−^) and/or heavy metals (e.g., Cd^2+^) (Latef et al. 2016). (**g)** Spore dispersal. Mycorrhizal spores (MS) decorated with AGLs could facilitate aerial dispersal and transport (Chaudhary et al. 2020). (**h)** Signalling molecules. AGLs, after cleavage of GPI-anchors, could be soluble-signalling molecules (in soil and/or in roots), secreted by various AMF structures (e.g., germinating spores or hyphae). Image adapted from Lanfranco et al. (2018). (**i**) Water movement. Zwitterionic AGLs could facilitate the apoplastic wicking of water, first along hyphal walls to the root, where water could continue through the fungal apoplast or the plant apoplastic or cell-to-cell routes as previously suggested for ectomycorrhizas (Lehto and Zwiazek 2011)

One requirement for functional analysis of AGLs is knowledge of their precise location (Fig. 8). Being GPI-anchored proteins, AGLs could exist in one or more cell surface locations (plasma membrane, soluble, or covalently bound to the cell wall) (Schultz and Harrison 2008; Müller 2018; Yeats et al. 2018; Urbar-Ulloa et al. 2019). Antibodies could be used to address their spatial localisation.

Based on published research, we can envisage that AGLs/IDPs could be involved in one or more of the following processes (Fig. 8c-i: “Cell–cell” interactions/self-recognition (Chagnon 2014; Essen et al. 2020); wall adaptability (strength and/or flexibility); retention of nutrients (Cavagnaro et al. 2015) or amelioration of toxic ions/heavy metals (Latef et al. 2016); soil stability (Rillig and Mummey 2006; Holátko et al. 2021); spore dispersal (Chaudhary et al. 2020); signalling molecules; and water movement (Lehto and Zwiazek 2011; Püschel et al. 2020). Many of these processes already have been studied in *R. irregularis*, and the different AGLs could contribute individually or collectively to these processes. *R. irregularis* is particularly good at improving soil stability (Kohler et al. 2017) and its unique AGLs could contribute to this trait. Testing whether one or more AGLs are present in high-temperature citrate extracts from soil (Irving et al. 2021), in addition to glomalin, is an exciting possibility for future research. A comparison of gene expression under stress conditions in related species (e.g., *R. irregularis* and *R. clarus*) may clarify whether AGLs play a role in stress tolerance (e.g., drought or salt stress). Studies of recombinant proteins from the different fungus species will allow us to compare properties of self-assembly as done for RiAGL1 and RiAGL3 (Creasey et al. 2012). Methods such as host-induced gene silencing (Hartmann et al. 2020) could be particularly useful in uncovering the roles of this intriguing family of cell surface proteins in the important plant–AMF symbiosis (Fig. 8).

## Supplementary information

Below is the link to the electronic supplementary material.

Supplementary file1 (DOCX 4685 KB)

Supplementary file2 (DOCX 93 KB)

Supplementary file3 (XLSX 30 KB)

Supplementary file4 (XLSX 26 KB)

Supplementary file5 (XLSX 18 KB)

Supplementary file6 (XLSX 16 KB)

Supplementary file7 (XLSX 20 KB)

Supplementary file8 (XLSX 18 KB)

## Data Availability

Sequences from the “wet-bench experiments” (Fig. S1a, c) are available from GenBank, https://www.ncbi.nlm.nih.gov, reference numbers MZ382300–MZ382308.
