# Supplementary material for: A targeted bioinformatics approach identifies highly variable cell surface proteins that are unique to Glomeromycotina
Source: Mycorrhiza. 2022 Jan 15;32(1):45–66. doi: 10.1007/s00572-021-01066-x (PMC8786786; doi:10.1007/s00572-021-01066-x)

**Supporting Information**

**Fig. S1** AGL sequences from a variety of arbuscular mycorrhizal fungi. For each species and sequence source the DNA sequences are provided first, followed by the protein translations. For DNA sequences, untranslated regions (UTRs) and introns are in lowercase text, and coding regions (uppercase). The ‘Oases’ DNA sequences have an additional 20 nucleotides (nt) of 5' and 3' UTRs to maximise the chance of getting full length sequences. Oases sequences also include, in the sequence ID line, the *k-*mer value (*k*39, *k*49, *k*59 or *k*69) of the assembly in which the sequence was found. ‘Mira’ sequences are only provided if they were clearly better than the Oases sequence. In some cases, the differences between Oases and mira are extensively annotated with comment lines starting with ‘#’ (see Sccal (Fig S1k,l)). Highlighted proteins (i–r) indicate the ones used for analysis of mature proteins. Protein translations are displayed with a line break between the predicted ER and GPI signal sequences (if present). The sequence header information incorporates the species name and information about the source of the sequence, with the following abbreviations (species first, then other information): Ri, *Rhizophagus irregularis;* Rc, *R. clarus;* Fumos, *Funneliformis mosseae; Sccal, Scutellospora calospora; Racas, Racocetra castanea; Pabra, Paraglomus brasilianum; Amlep, Ambispora leptoticha; Rhcer, R. cerebriforme; Gimar, Gigaspora margarita, Giros, Gigaspora margarita;* rc, reverse complement; pm20 = plus minus 20 nt additional 5' and 3' UTRs respectively (in lower case); The nt counts at the end of the header information are for CDS only, and amino acid (aa) counts include the signal peptides.

Sequences from the “wet-bench experiments” (Fig. S1a,c) are available from GenBank, https://www.ncbi.nlm.nih.gov, reference numbers MZ382300–MZ382308.

**References:** Beaudet *et al.,* 2018. DNA Research 25: 217-227; Kobayashi *et al.,* 2018. BMC Genomics 19; Maeda *et al.,* 2018. Communications Biology 1.

**Summary of sequences**

(a) RiAGL_gDNA_PCR products

(b) RiAGL_protein_PCR products

(c) RcAGLs_gDNA_PCR products

(d) RcAGL_protein_PCR products

(e) RiAGL_PacBio_gDNA

(f) RiAGL_PacBio_protein

(g) RcAGL_PacBio_gDNA

(h) RcAGL_PacBio_protein

(i) Fumos_oases (cDNA, then protein)

(j) Fumos_mira (cDNA, then protein)

(k) Sccal_oases (cDNA, then protein)

(l) Sccal_mira (cDNA, then protein)

(m) Racas_oases (cDNA, then protein)

(n) Racas_mira (cDNA, then protein)

(o) Amlep_oases (cDNA, then protein)

(p) Amlep_mira (cDNA, then protein)

(q) Pabra_oases (cDNA, then protein)

(r) Pabra_mira (cDNA, then protein)

(s) Gimar, NCBI BLASTn (gDNA, cDNA, then protein)

(t) Giros, NCBI BLASTn (gDNA, then protein)

(u) Rhcer, NCBI BLASTn (gDNA, then protein)

***see separate file for the sequences***

**Fig. S2** BLASTp and BLASTn search results show the difficulties with identifying AGLs in annotated genomes. BLAST results are summarized for each query sequence, the partial *RiAGL* (Fig. S1a) and *RcAGL* (Fig. S1b) DNA and encoded protein sequences obtained by degenerate primer PCR. The query sequence is indicated by a number (eg 1, AGL1), above a solid line. Two solid arrowheads indicate the approximate location of the predicted cleavage sites of the N-terminal ER and C-terminal GPI-anchor signal sequence (if present). The coloured rectangles (approximately to scale) depict the coverage of the query AGL sequence compared to the matching protein (BLASTp) or for BLASTn, the %ID of the annotated protein of the corresponding nucleotide sequence (if present). A dashed grey box is used to indicated the full length protein where coverage is significantly <100%. For BLASTn, the coverage and %ID were all high ranging from 84–100% and %ID from 89.1–100%, however the annotated protein was often wrong (Table S3). Manual annotation was used to obtained full length AGL sequences for subsequent analyses (Fig. S1e–h)). *Ri* genome 1 (PRJDB4945, Maeda *et al.,* 2018), *Ri* genome 2 (PRJNA208392, Tisserant *et al*., 2013), *Ri* genome 3 (PRJNA230015, Lin et al 2014), *Rc* genome 1 (PRJDB6444, Kobayashi *et al.,* 2018), *Rc* transcriptome 1 (Sędzielewska Toro and Brachmann, 2016). Accession numbers, E-values, sequences and predicted cleavages sites are indicated in (Table S3).

**References:** Kobayashi *et al.,* 2018. BMC Genomics 19; Lin *et al.,* 2014. PLoS Genetics 10. Maeda *et al.,* 2018. Communications Biology 1; Sędzielewska Toro and Brachmann, 2016. BMC Genomics 17; Tisserant *et al*., 2013. Proc Natl Acad Sci 110: 20117-20122.


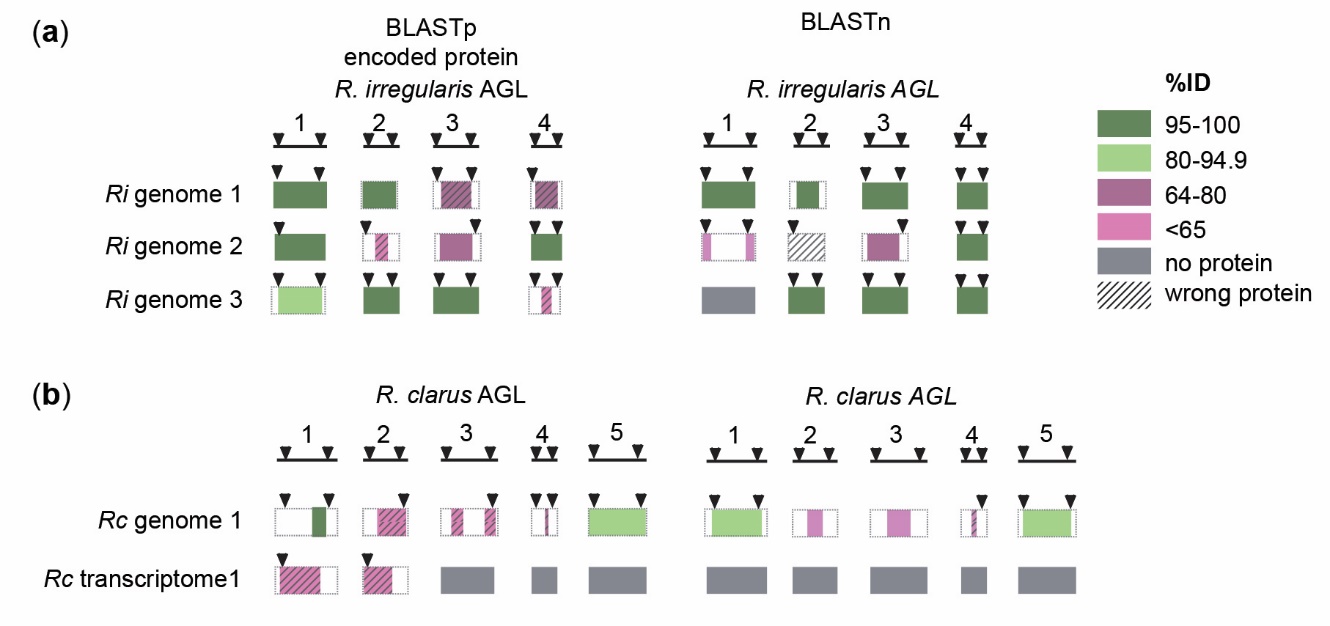


**Fig. S3** Pairwise alignment between pairs of potentially orthologous AGL gene sequences from *R. irregularis* and *R. clarus.* Alignment of full length PacBio gene sequences were performed with EMBOSS Needle (DNA, <https://www.ebi.ac.uk/Tools/psa/emboss_needle/>). *RiAGL1* (Fig. S1e) and *RcAGL1* (Fig. S1g) (see Fig. 2), (a) *RiAGL2* (Fig. S1e) and *RcAGL2* (Fig. S1g), and (b) *RiAGL3* (Fig. S1e) and *RcAGL3* (Fig. S1g). The translated protein sequences are superimposed, with the corresponding DNA sequence. Intron sequences in lower case blue text. Conserved nucleotides are indicated by * (coding) and | (intron) and semi-conserved bases (.). The sequence encoding the cleaved portions of the N-terminal ER and C-terminal GPI-anchor signals are in green text. The N-terminal Q (glutamine) residue of the predicted mature proteins (RiAGL1 and AGL3, not AGL2) are highlighted (pink). Gaps in the coding sequences that generate frameshifts (not multiples of three (potential codons)) are shaded light red. Repeat motifs (Figs 3,7) are highlighted for each translated protein sequences: GATPPA of RiAGL2 and RcAGL2 (grey); and APKDG of RiAGL3 (green) and RcAGL3_TR1 (APGGGGGA) (blue), RcAGL3_TR2 (GAAPGG, pink) and RcAGL3_TR3 (PPPGGAPGGGA, yellow) (Table S9).

Figure on next page

(**a**)

RiAGL2 1 ATGGCAAAATTCACAAAATTAACGTTCGTTTGCGTTCTTGTCCTCGTTCTCCTTGTTTCCTTCAGCGTATCATCTCCTGgttagtaaattaatccctt-- 98

M A K F T K L T F V C V L V L V L L V S F S V S S P E||||||||||.|.| ||

M A K F S K L T I V C V L V L V L L V S F S V S E

*RcAGL2*  1 ATGGCCAAATTCTCAAAATTAACGATCGTTTGCGTTCTCGTTCTCGTCCTTCTTGTTTCCTTCAGTGT------TTCTGgttagtaaataagt---ttta 91

*****.******.***********.*************.**.*****.**.**************.** *.***

*RiAGL2*  99 tttacggtttc----ttttttggttaaa--aattccttttatt-------taatttattca-----ttttattttttttc---------ttaacagAACG 171

||| ||||| ||||||.|||||| ||| |.||||| ||||||||||| ||||.||||||||| ||||||| R

||| ||||| ||||||.|||||| ||| |.||||| ||||||||||| ||||.||||||||| ||||||| R

*RcAGL2*  92 ttt---gtttcctttttttttagttaaattaat---tattattcaatttataatttattcacattttttttttttttttctctcaatcattaacagAACG 185

****

*RiAGL2* 172 ATTAGCTGTTCGTCAAGCACCTACACCA----GATACTGGAGCTCCAGCACCAGGAGGTGATGCAACTCCACCAGCA------------------GGTGG 249

L A V R Q A P T P D T G A P A P G G D A T P P A G G

L A V R Q A P A P A G G A P P A G G A P P A G G A G G A G G

*RcAGL2* 186 ATTAGCTGTTCGTCAAGCACCAGCACCAGCAGGA----GGAGCACCACCAGCTGGAGG------AGCACCACCAGCAGGAGGAGCTGGAGGAGCTGGAGG 275

*********************..***** ** *****.***.**.*.***** *.*.********* **.**

*RiAGL2* 250 AGCCACACC------------------AGGAGCAACTCCACCAGC---------------AGCTGGAGGAG---CACCACCAGCAGGAGCAACCCCACCA 313

A T P G A T P P A A G G A P P A G A T P P

A T P P A G G A G G A T P P A G G A G G A G G A T P P A G G A P P

*RcAGL2* 276 AGCCACACCACCAGCAGGAGGAGCTGGAGGAGCTACACCACCAGCAGGAGGAGCAGGAGGAGCTGGAGGAGCCACACCACCAGCAGGAGGAGCACCACCA 375

********* ******.**.******** *********** ***************.*.*.******

*RiAGL2* 314 GCAGCTGGAGGAGCACCACCAGCAGGAGCAACCCCACCAGCAGGAGCAACTCCACCAGCAGGAGGAGCACCACCAGCAGGAGGTCC---------ATCAT 404

A A G G A P P A G A T P P A G A T P P A G G A P P A G G A G A

A G G D A G A G G A T P P A G G A P P A G G A P P A G G A A G G A S

*RcAGL2* 376 GCAGGAGGAGATGCAGGAGCAGGAGGAGCTACACCACCAGCAGGAGGAGCACCACCAGCTGGAGGAGCACCACCAGCAGGAGGAGCAGCAGGAGGAGCAT 475

****..****..***..*.***.******.**.*************.*.*.********.***********************..* *.***

*RiAGL2* 405 CATCACCTTCTTCATCACCAACTGTGAGCGCTTCAGCTGCCGGCCCTAGTG--------GTAGCT----CATCTCCAGCAG---CAACAGGCGCTGCCTA 489

S P S S S P T V S A S A A G P S G S S S P A A T G A A Y

S A G A P T P S S S M A S P S A S G G A A A A P S A S T S A A F

*RcAGL2* 476 CATCAGCTGGT---GCCCCAACTCCCAGTTCTTCAATGGCCAGCCCAAGTGCATCAGGAGGAGCTGCTGCAGCCCCATCAGCTTCCACTAGTGCTGCCTT 572

*****.**..* .*.******...**..*****...***.****.*** * *.**** **.*.***.*** *.**..*.*******.

*RiAGL2* 490 CAAAATTGAAAGTGGTTTATCTAGCGTTGTTGCCCTCGCTGCTCTCGTTGGTTATTTCTTGTAA 553

K I E S G L S S V V A L A A L V G Y F L *

K V E S G L S S I A A L T A L V G F F L *

*RcAGL2* 573 CAAAGTTGAAAGTGGTTTATCCAGCATTGCTGCCCTTACCGCTCTCGTTGGTTTCTTCTTGTAA 636

****.****************.***.***.******..*.*************..*********

(**b**)

*RiAGL3*  1 ATGAAATTTAATAATCGTATCTTCTTTCTTCTTATTGTTCTCACCGTATTGATTGCCTGCGTTGCTGCACAgtaag---ttttttcctaaagaaaaaaa- 96

M K F N N R I F F L L I V L T V L I A C V A A Q ||||| |||||||||.||.|||||||

M K F N K R I F F L L T G L A V L I A C V A A Q ||||| |||||||||.||.|||||||

*RcAGL3*  1 ATGAAATTTAATAAACGTATCTTCTTTCTACTTACTGGTCTCGCTGTATTGATTGCCTGCGTTGCTGCACAgtaagtatttttttccttaaaaaaaaaac 100

**************.**************.****.**.****.*.**************************

*RiAGL3*  97 -------ttttaatgactgaat-agataaaataaact-tcttgtattgtgataatagGGGACCAGG------AGGAGCACCAGGAGCACCAG------CA 175

|||||||||.||.|| ||||||.|.||.|| ||.| ||||.||||||| G P G G A P G A P A

|||||||||.||.|| ||||||.|.||.|| ||.| ||||.||||||| A P G A P G A P G A P G A P

*RcAGL3* 101 aaaaggtttttaatgattgtataagataataaaatctctcat---ttgtaataatagAGCACCAGGAGCACCAGGAGCACCAGGAGCACCAGGAGCACCA 197

.*.****** ******************** **

*RiAGL3* 176 CCAGCACCA------------GGAGCACCACCACCAGATGG---------AGCACCTAAAGATGG------------AGCACCCAAAGATGG------AG 236

P A P G A P P P D G A P K D G A P K D G A

P A P G G G G G A A P G G G G G A A P G G G G G A A P G G G A A

*RcAGL3* 198 CCAGCACCAGGAGGTGGAGGTGGAGCAGCACCAGGAGGTGGAGGTGGAGCAGCACC---AGGTGGAGGAGGTGGAGCAGCACC---AGGTGGAGGAGCAG 291

********* ******.*****..**.*** ****** **.*** ****** **.*** **

*RiAGL3* 237 CACC------TAAAGATGGAGCACCCAAAGATGGAGCAC-------------------------CCAA------------AGATGGAGCACC--CAAA-- 289

P K D G A P K D G A P K D G A P K

P G G G G G A P G G G A A G G G G G G K A K V T P P G G A P A K G

*RcAGL3* 292 CACCAGGAGGTGGAGGTGGAGCACCAGGAGGTGGAGC-CGCGGGAGGTGGAGGTGGAGGTAAGGCCAAGGTAACTCCACCAGGAGGAGCACCAGCAAAAG 390

**** *..**.*********...**.****** * **** **..******** ****

*RiAGL3* 290 ---------------GATGGAGCACCTAAAGATGGAG--------CCA----------------------------------------AAGGAGCAC--C 324

D G A P K D G A K G A P

A A P G G G A P P A G G A A P G G G A A P G G G A A P G G G A P P

*RcAGL3* 391 GAGCAGCACCAGGAGGA-GGAGCACCACCAGCAGGAGGAGCAGCACCAGGAGGAGGAGCAGCACCAGGAGGAGGAGCAGCACCAGGAGGAGGAGCACCAC 489

** ********...**..**** *** .******** *

*RiAGL3* 325 CA-----AAGATGCAC--CCA-----AAGATGCACC----CAAAGATG-----CAGGAGGTGATGCTA-----------------------AGGGTAAAG 380

K D A P K D A P K D A G G D A K G K V

A G G A P P P G G A P P P G G A P G G G A P P P G G A P G G G

*RcAGL3* 490 CAGCGGGAGGA-GCACCACCACCAGGAGGA-GCACCACCACCAGGAGGAGCACCAGGAGGCGGAGC-ACCACCACCAGGAGGAGCACCAGGAGG----CG 582

** *.** **** *** *.** ***** *.*.**.* *******.*..** * *** .*

*RiAGL3* 381 TAGCTCCGCCAGCAGGAGGAGCACCACCAGCAGGAGGAGCACCAGCAGG------------------------ACCACCACCAGAAGGAGCAGCACCATC 456

A P P A G G A P P A G G A P A G P P P E G A A P S

A P P A G G A P P P G G A P G G G A T P A G G A P P P E G A S P S

*RcAGL3* 583 GAGCACCACCAGCAGGAGGAGCACCACCACCAGGAGGAGCACCAGGAGGTGGAGCAACACCAGCAGGAGGAGCACCACCACCAGAAGGAGCATCACCATC 682

.***.**.*********************.***************.*** *******************.*******

*RiAGL3* 457 ACCAGCGAAAACAGCCGCACCTACTCCAGGTGGAGGAACAGGTACATCAGTTGCTCCAGCAGGAGCATCAGGAAGCACACCTGCCAAATCAGCAACAGGA 556

P A K T A A P T P G G G T G T S V A P A G A S G S T P A K S A T G

P A T S T P P T P G P G T G T S A T P P G A S G S A A K T T A

*RcAGL3* 683 ACCAGCGACATCAACTCCACCTACTCCAGGTCCAGGAACTGGTACATCAGCTACTCCACCAGGAGCATCAGGAAGTGCA---GCCAA---AACAACAGCA 776

********.*.**.*..**************..******.**********.*.*****.****************..** ***** *.******.*

*RiAGL3* 557 GCCGGAAACAGTCTTAAAGCCGAAGTCGGAGTTTCATTCGCAGCTGT---AATTCTTGGTGCTATATTTGCTTAA 628

A G N S L K A E V G V S F A A V I L G A I F A *

A G N S L K S E V G I S F A A V A I L G A I L A *

*RcAGL3* 777 GCTGGAAACAGTCTTAAATCTGAAGTCGGAATTTCATTCGCCGCTGTTGCAATTCTTGGTGCTATATTAGCTTAA 851

**.***************.*.*********.**********.***** ******************.******

**Fig. S4** Phylogenetic analysis supports orthology of *AGL1* and *AGL3* genes from *R. irregularis* and *R. clarus*. (a) Maximum likelihood (ML) tree generated from a multiple sequence alignment (b) of the conserved exon sequences of *RiAGL1, RiAGL3, RiAGL4, RiAGL5, RcAGL1, RcAGL3, RcAGL4, RcAGL5* and *RcAGL6. RiAGL2* and *RcAGL2* sequences were excluded due to low sequence identity with the other AGLs (data not shown). The genes that align well, at least for the ER/GPI-signal sequences (b), are the ones that are co-located in the genome and contain a generally higher number of tandem repeats (Figs 3, S3, Tables S4,S5). Identical bases are indicated with an asterisk (*). The best ML model (T92+I) was found using MEGA X (Kumar *et al.,* (2018) Mol Biol Evol 35:1547-1549). Scale bar for branch length measures the number of substitutions per site. Numbers on the nodes represent support with 1000 bootstrap replicates. The alignment comprises the entire exon 1 sequence (encoding the ER signal and a few additional amino acids, 65-71nt), the first nine nucleotides (nt) of exon 2, and the final 81–84 nt of exon 2 (encoding some of the mature C-terminus and all of the GPI-signal). (c) DNA sequence alignment of the full length gene sequences of *RiAGL1, RiAGL3, RiAGL4, RiAGL5, RcAGL1, RcAGL3, RcAGL4, RcAGL5* and *RcAGL6.* The conserved sequences used for phylogenetic analysis (Fig. S4a) are indicated in blue text. The translation for the conserved regions of RiAGL1 includes the N-terminal ER- and C-terminal GPI-anchor signals (green text above the alignment). The N-terminal Q (glutamine) residue of the predicted mature RiAGL1 is highlighted (pink). The asterisks below the conserved sequences are the same as in Fig. 4b, but in one case a gap has been introduced due to the use of full length sequences. Note that most of the coding sequence for the mature AGL proteins was not suitable for phylogenetic analysis due to the large number of gaps and low % identity (Dwivedi & Gadagkarc, 2009. BMC Evol Biol 9: 211). Only 57 out of 790 aligned bases (7.2%, black text) are 100% identical (#, hash symbol) in all 9 sequences. Full length sequences were aligned in Genious 8.1.9, using Muscle (Distance measure: kmer4_6 (Iteration 1), pctid_kimura (subsequent iterations (max of 8); Clustering method (UPGMB); gap open score (-1)). (d) Comparison of % DNA identity for selected full length AGL genes and the conserved regions used for phylogenetic analysis (a,b), were generated from pairwise DNA alignments using EMBOSS Needle (<https://www.ebi.ac.uk/Tools/psa/emboss_needle/>).

Figure on next page

(**a**)


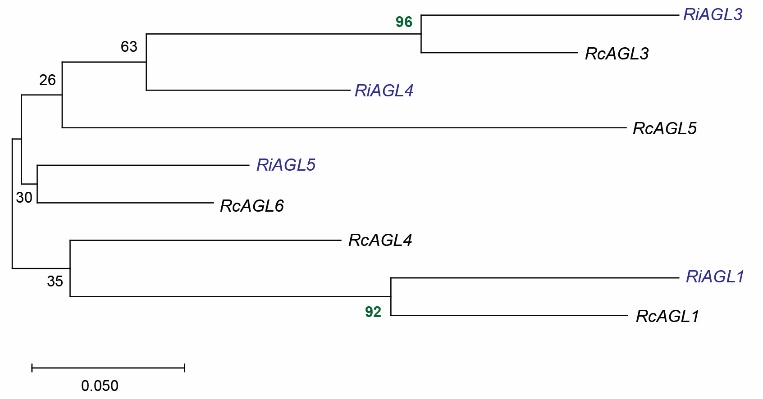


(**b**)

**<--------------------------- EXON 1---------------------------------->**

*RiAGL1* ATG------AATAAACGCATCTTCATTATTCTCGCCATTTTCTTCGCTTTAGTTACCTACGTTGCTGCACA

*RcAGL1* ATG------AATAAACGCATTATCTTTCTTCTCGCCGTTCTCTTAGCTTTAGTAACTTATGTTGCTGCACA

*RiAGL3* ATGAAATTTAATAATCGTATCTTCTTTCTTCTTATTGTTCTCACCGTATTGATTGCCTGCGTTGCTGCACA

*RcAGL3* ATGAAATTTAATAAACGTATCTTCTTTCTACTTACTGGTCTCGCTGTATTGATTGCCTGCGTTGCTGCACA

*RiAGL4* ATGAAATTCAATAAACGCATCCTCTTTCTTCTTGCTGTTCTCATTGTATTGATTGCCTGCGTTGCTGCACA

*RcAGL4* ATGGCATTCAATAAACGCATCATCCTTCTTCTTACTGTTCTCGCCGTGTTAATTGCCTGCGTTGCTGCACA

*RiAGL5* ATGAAATTCACTAAACGTACCTTCTTTCTTCTTGCTGTTCTCACTTTGTTGATTGCCTGTGTTGCTGCACA

*RcAGL5* ATGAAATTCAATAAACGTATCTTATTT---CTTATTGTCTTCACTGTGTTGTTTATCTATGTTGCTGCACA

*RcAGL6* ATGAAATTCAATAAACGTACTTTCTTTCTTCTTGCTGTCTTCACTGTGTTGATTGCTTGCGTTGCTGCACA

*** * *** ** * * ** ** ** ** * * ***********

**EXON 2 EXON 2**

**<- 5’ -><-------------------------------------- 3’ end ------------------------------------>**

*RiAGL1* AGGACCAGCGCAGCATCATCCGGAAATTCTCTTAAATC---ATCTGGATATTCATTCGCTGCCATTGCCGTTCTTGGAGCTATCTTCGCTTAA

*RcAGL1* AGCACCAGCACAGCATCAGCTGGAAGTTCTCTTAAATCAGAAGCTGGAGTTTCATTCGCTGCCATTGCCGTTATTGCTGCTATCCTCGCTTAA

*RiAGL3* GGGACCAGGGCAACAGGAGCCGGAAACAGTCTTAAAGCCGAAGTCGGAGTTTCATTCGCAGC---TGTAATTCTTGGTGCTATATTTGCTTAA

*RcAGL3* AGCACCAGGACAACAGCAGCTGGAAACAGTCTTAAATCTGAAGTCGGAATTTCATTCGCCGCTGTTGCAATTCTTGGTGCTATATTAGCTTAA

*RiAGL4* GGTACCAGGGCAACAGGATCCGGAAATTCTCTTAAATCTGAAATTGGAGTTTCATTCGTTGCTATTGCCGTTCTTGGTGCTATCCTCGCTTAA

*RcAGL4* AGCACCAGCACAGCATCAGCCGGAAATTCTCTTAGATCTGAAGTTGGAGTTCCATTCGCTGCCATTGCCATTCTTGGTGCTATCCTTGTCTAA

*RiAGL5* AGGACCAGCGCAGCATCATCCGGAAATACTCTTAAATC---ATCTGGAGTTTCATTCGCTGCCATTGCCGTTCTTGGTGCTATCTTCGCTTAA

*RcAGL5* GGAACCAGGACAGTATCGGCTGGAAATTCTCTTGAATCTGAAGCTGGAATTTCATTTGTTGCAATTGCCATCCTTGGTGCTATTTTCGGTTAA

*RcAGL6* AGCACCAGCACAGCATCAGCCGGAAACTCTCTTAAAGCTGAAGTTGCAGTTTCATTCGCTGCCATTGCCGTTCTTGGTGCTATCTTCGCTTGA

* ***** ** * * **** **** * * * * * * **** * ** ** * *** ***** * * * *

(**c**)

M N K R I F I I L A I F F A L V T Y V A A Q

*RiAGL1*  ATG------AATAAACGCATCTTCATTATTCTCGCCATTTTCTTCGCTTTAGTTACCTACGTTGCTGCACAgtaagtttccttcac-aattattattcaagaaaagaataattaatataa

*RcAGL1*  ATG------AATAAACGCATTATCTTTCTTCTCGCCGTTCTCTTAGCTTTAGTAACTTATGTTGCTGCACAgtaagttttttccgcgattaattattcaaaggaaaacgaaaaaataatg

*RiAGL3*  ATGAAATTTAATAATCGTATCTTCTTTCTTCTTATTGTTCTCACCGTATTGATTGCCTGCGTTGCTGCACAgtaagttttttcc-------------taaagaaaaaaattttaatgact

*RcAGL3*  ATGAAATTTAATAAACGTATCTTCTTTCTACTTACTGGTCTCGCTGTATTGATTGCCTGCGTTGCTGCACAgtaagtatttttttc---------cttaaaaaaaaaacaa-----aagg

*RiAGL4*  ATGAAATTCAATAAACGCATCCTCTTTCTTCTTGCTGTTCTCATTGTATTGATTGCCTGCGTTGCTGCACAgtaagtttttttccc-------atgaaaaaaaaaaaataataaataaaa

*RcAGL4*  ATGGCATTCAATAAACGCATCATCCTTCTTCTTACTGTTCTCGCCGTGTTAATTGCCTGCGTTGCTGCACAgtaagttatttttct-----------tggaaaaaaaacgtatactaact

*RiAGL5*  ATGAAATTCACTAAACGTACCTTCTTTCTTCTTGCTGTTCTCACTTTGTTGATTGCCTGTGTTGCTGCACAgtaagtttttttttt------tccctgaaaaaaaaaaataataataaat

*RcAGL5*  ATGAAATTCAATAAACGTATCTTATTT---CTTATTGTCTTCACTGTGTTGTTTATCTATGTTGCTGCACAgtaagt--------------------ggaaaaaaaaaaaaataattttt

*RcAGL6*  ATGAAATTCAATAAACGTACTTTCTTTCTTCTTGCTGTCTTCACTGTGTTGATTGCTTGCGTTGCTGCACAgtaagttttttcttc-------ctgaaaaaaaaaaacaaataaataaaa

*** * *** ** * * ** ** ** ** * * ***********###### ## #

*RiAGL1*  tatggtgaagttgt----------taatcaaatcactcttttt------tatgataacagAGGACCAGCTGACGG---AGCACCA---GCTGACGGAAAAGCACCAGCTGACGGAGCACC

*RcAGL1*  taataaaaaatccc------------------taaccacttttttt---tatgataacagAGCACCAGCAGGAGG---AGCACCAGATGCAGGAGCAGGAGCACCACCAGCAGGAGGAGC

*RiAGL3*  gaatagataa--------------------aataaacttcttgtat---tgtgataatagGGGACCAGGAGG------AGCACCAGGAGCACCAGCACCAGCACCA--------------

*RcAGL3*  tttttaatgattgt---------ataagataataaaatctctcatt---tgtaataatagAGCACCAGGAGCACCAGGAGCACCAGGAGCACCAGGAGCACCACCAGCACCAGGAGGTGG

*RiAGL4*  aaattaataatgat----------tgaataaataaatcctcatatt---tgtgataatagGGTACCAGGGGCACC---AGCACCAGGAGGCGACGGAAAAGCTCCAGCAG----------

*RcAGL4*  gcatgcgtataaattttataaagttgaataaataaatccctttgctttctgtgttaatagAGCACC------------AGCACCACCAGCTGGCGGAAAAGCCCCTACTTC---------

*RiAGL5*  tttttaatagttta---------ataaataaaaaatatttcttgtt---tatgataatagAGGACCAGCTGACGGAAAAGCTCCA---GCTGACGGAAAAGCTCCAGCTGACGG------

*RcAGL5*  taatggataa-------------ataaataaataaacctcttgcta---cgggataatagGGAACCAGG---------AGCACCAGCAGCACCAGCAGCAGCACTAGCAAAGGCACCTGA

*RcAGL6*  taaataaaaaatattttgaatagataaataaataattctttttgtt---tatgataatagAGCACCAGCACC------AGCACCAGGTGATGCAGGAAAAGCCGCTCCAGCGGG------

# ## * ***** ### ### # # # #

*RiAGL1*  AGCTGACGGAAAAGCACCAGCT---GACGGAGCACCAGC------------------CGACGGAAAAGCACCAGCTGACGGAGCACCAGCCGACGGAAAAGC---------ACCAGCCGA

*RcAGL1*  ACCAGATGCAGGAGCAGGAGCA---GGAGGAGCACCACCAGCACCAGACGCAGGAGCAGGAGGAGCAGGAGCACCAGACGCAGGAGCAGG---AGGAGCAGGAGCACCACCAGCAGGAGG

*RiAGL3*  ----------------------------GGAGCACCACC---------------------------------ACCAGATGGAGC---------------------------ACCTAAAGA

*RcAGL3*  AGGTGGAGCAGCACCAGGAGGTGGAGGTGGAGCAGCACC------------AGGTGGAGGAGGTGGAGCAGCACCAGGTGGAGGAGCAGCACCAGG---------------AGGTGGAGG

*RiAGL4*  ------------------------------------------------------------------------------------------------------------------------

*RcAGL4*  ------------------------------AACACTACC---------------------------------------------------------------------------------

*RiAGL5*  ---------AAAAGCTCCAGCT---GATGGAAAAGCTCC---------------AGCCGACGGAAAAGCCCCAGCTGACGGTGC---------------------------TGCTGACGG

*RcAGL5*  AGGTGAAGCACCTAAAGGGGAT---GGCGTAAAGGTACCTAA------------GGGTGATGGAAAAGCACCTAAAGACGCAGGAGCACCTAAAGCAGAAGCTAAAGGCAAGGCTGGAGG

*RcAGL6*  ---------------------A---GGTGCCGCACCACC---------------AGCAGGAGGAAAAGCCGCTCCAGATGCAGGA------------------------------AAAGG

*RiAGL1*  CGGAGCACCAGCCGACGGAAAAGCACCAGACGCAGCCG------------------------ACGGAAAAGCACCAGCTGACGGAAAAGCACCAGCTGA---------CGGAAAAGCACC

*RcAGL1*  AAAAGCCCCAGCAGGAGGTGCCGCCCCAGGTGCTGATG------------------------CAGGAAAAGCCCCAGCAGGAGGTGCCGCCCCAGGTGCTGATGC---AGGAAAAGCCCC

*RiAGL3*  TGGAGCACCCAAAGATGGAGCACCTAAAGATGGAG---------------------------------------CACCCAAAGATGGAGCACCCAAAGA---------TGGAGCACCCAA

*RcAGL3*  TGGAGCACCAGGAGGTGGAGCCGCGGGAGGTGGAGGTGGAGGTAAGGCCAAGGTAACTCCACCAGGAGGAGCACCAGCAAAAGGAGCAGCACCAGGAGG---------AGGAGCACCACC

*RiAGL4*  --------------------------------------------------------------------------CACCAGGAGGTGACGC------------------GGGAAAAGCACC

*RcAGL4*  ------------------------------------------------------------------------------------------------------------GGGAAAAGCTCC

*RiAGL5*  AAAAGCCCCAGCCGACGGAAAAGCCCCAGCTGACG------------------------------GAAAAGCTCCAGCTGACGGAAAAGCTCCAGCTGA---------TGGAAAAGCTCC

*RcAGL5*  TGCTCCACCAGCAAAAGGTCCACCAGGAGGTGCTCCAC------------------------CAGCAGGAGGTCCACCAGGAGGTGCCCCACCAGCAGGAGGTCCACCAGGAGGCGCCCC

*RcAGL6*  TGCTCCACCAGCAGGAGGTGCCGCTCCAGATGCAG------------------------------GAAAAGCTCCAGCGGGAGGCGCTGCCCCACCAGCAGG------AGGAAAAGCCCC

### #

*RiAGL1* AGCCGACGGAAAAGCACCA------GCTGACGGAAAAGCACCAGCCGACGGAAAAGCACCA------GCTGACGGAAAAGCTCCAGCTGACGGAAAAGCTCCAGCTGACGGAAAAGCACC

*RcAGL1* AGCCGGAGGTGCCGCCCCAGGTGCTGATGCAGGAAAAGCCCCAGCCGGAGGTGCCGCCCCAGGTGCTGATGCAGGAAAAGCCC---CAGCAGGAGGTGCCGCCCC---------AGGTGC

*RiAGL3* A------------------------------------------------------------------------GATGGAGCACCTAAAGATGGAG-------------------------

*RcAGL3* AGCAGGAGGAGCAGCACCA------GGAGGAGGAGCAGCACCAGGAGGAGGAGCAGCACCA---------GGAGGAGGAGCACCACCAGCGGGAGGAGCACCACCACCAGGAGGAGCACC

*RiAGL4* AGC------------------------------------------------ACCAGCACCA---------GGCGGAAAAGCTCCA------------------------------GCTCC

*RcAGL4* A------------------------------------------------------------------------GGGGGTGCCACAAACACTTTAAG------------------------

*RiAGL5* AGCTGATGGAAAAGCTCCA------GCTGACGGAAAAGCTCCAGCTGACGGAAAAGCCCCA------GCCGACGGAAAAGCCCCAGCTGATGGAAAAGCTCCAGCTGATGGAAAAGCTCC

*RcAGL5* ACCAGCAGGAGGTCCACCA------------GGAGGCGCCCCACCAGCAGGAGGTCCACCA------------GGAGGCGCTCCACCAGCAGGAGGTCCACCA------GGAGGCGCTCC

*RcAGL6* AGCA---------------------GATGCAGGAAAAGCCCCAGCAGGAGGTGCTGCCCCA------CCAGCAGGAGGTGCTCCACCAGCAGGAGGTGCT---------------GCCCC

# # ##

*RiAGL1*  AGCTGACGGAAAAGCACCAGCTG------ACGGAAAAGCTCCAGCTGACGGAAAAGCTCCAGCAGGTGCTGCTCCAGGAGGTGCTGCTCCAGCTCCAGGTGCTGCTC-------------

*RcAGL1*  CGATGCAGGAAAAGCCCCAGCCG------GAGGTGCCGCCCCAGCCCCAGGTGCCGATGCAGGAAAAGCCCCAGCCGGAGGTGCCGCTCCCCCAGCAGGAGCTGCTC-------------

*RiAGL3*  -----CCAAAGGAGCACCCAAAGATGCACCCAAAGATGCACCCAAAGATG---------CAGGAGGTGATGCTAAGGGTAAAGTAGCTCCGCCAGCAGGAGGAGCAC-------------

*RcAGL3*  ACCACCAGGAGGAGCACCAGGAG------GCGGAGCACCACCACCAGGAGGAGCACCAGGAGGCGGAGCACCACCAGCAGGAGGAGCACCACCACCAGGAGGAGCACCAGGAGGTGGAGC

*RiAGL4*  AGCACCAGGAGGCGCTCCAGCTG------GCGGTAAAGCTCCAGCAGGCGATG---------------------CAGGAAAAGCTGCCCCA---------GGCACTC-------------

*RcAGL4*  ---------------------------------------------------------------------------------------TCCAACAACAGGAGGCGCTC-------------

*RiAGL5*  AGCTGACGGAAAAGCTCCAGGTGCTGCTCCAGGTGGTGCTCCAGCTCCAGGTGCTGCTCCAGGTGCTGCTCC------AGGTGCTGCTCCAGCA------GGAGCTC-------------

*RcAGL5*  ACCAGCAGGAGGTCCACCA------------GGAGGTGCTCCACCAGCCGGAGGTCCACCAGGAGGCGCTCCACCAGCAGGAGGCGCTCCACCAGCA---GGTGCCC-------------

*RcAGL6*  ACCAGCAGGAGGTGCTCCACCAG------CAGGAGGTGCTCCACCAGCAGG---------AGGCGCTGCCCCACCAGCAGGAGGTGCTCCAGCT------GACGCTC-------------

## # # #

*RiAGL1*  --CAAAAGACGGAAAAGCACCAG------CCCCAGCTGACGGAAAAACACCAGCAGCA------TCACCAGCA------------GGAGGAGCAGCCCCATC------AGCATCAGCCAA

*RcAGL1*  --CACCAGCAGGAGGTGCCACAC------CATCAGCAGGTACCGCAACCCCACCAGCA------GGAGCAT---------------------------CACC---AAAAGCATCAACAGG

*RiAGL3*  --CACCAGCAGGAGGAGCACCAGCAGGACCACCACCAGAAGGAGCAGCACCATCACCAGCGAAAACAGCCGCACCTACTCCAGGTGGAGGAACAGGTACATC---AGTTGCTCCAGCAGG

*RcAGL3*  AACACCAGCAGGAGGAGCAC---------CACCACCAGAAGGAGCATCACCATCACCAGCGACATCAACTCCACCTACTCCAGGTCCAGGAACTGGTACATC---AGCTACTCCACCAGG

*RiAGL4*  --CACCAGCAGGAGGTCCACCAGGAGTAACACCATCAGGTTCGGCATCACCACCGGCA------GCAACACCATCAACATCAAGCGGGACTGCTGCCGCACC---AAAATCATCACCAGG

*RcAGL4*  --CACCAGCGGGAAGTC------------CACCATCAGGTTCAACGTCGTCATCACCA------TCAGCATCA------------------------GCATC---AGCATCTCCATCAGG

*RiAGL5*  --CACCAGCAGGAGGTCCAAAAGGAGTAACACCAGCAGGCACAGCAACACCACCACCA------CCACCACCA---------AACAGTGCTGCTTCCGCACCAAAAACATCATCAACAGG

*RcAGL5*  --CACCAGCAGGAGGTCCACCAGGAGGCGCTCCACCAG---CAGGGGGTCCACCAGGAGGTGCCCCACCAGCCGGAGGTCCAGCAGGAGGCGCTCCACCAGCAGGAGGTCAAATAACAGA

*RcAGL6*  --CACCAGCAGGAGGTCCAGCACCAGTTACACCAGCAGGTACAGCAACACCACCAACA------CCACCACCA---------------TCTGCTGGTGCACC---CTCACCATCAACAGG

## # ### # ## # ## # ## # # # # # #

A A S S G N S L K S S G Y S F A A I A V L G A I F A *

*RiAGL1*  AGCAG------------------------CAGCATCATCCGGAAATTCTCTTAAATC---ATCTGGATATTCATTCGCTGCCATTGCCGTTCTTGGAGCTATCTTCGCTTAA

*RcAGL1*  AGCAACAG---------------------CAGCATCAGCTGGAAGTTCTCTTAAATCAGAAGCTGGAGTTTCATTCGCTGCCATTGCCGTTATTGCTGCTATCCTCGCTTAA

*RiAGL3*  AGCATCAGGAAGCACACCTGCCAAATCAGCAACAGGAGCCGGAAACAGTCTTAAAGCCGAAGTCGGAGTTTCATTCGCAGC---TGTAATTCTTGGTGCTATATTTGCTTAA

*RcAGL3*  AGCATCAGGAAGTGCAGCCAAAA------CAACAGCAGCTGGAAACAGTCTTAAATCTGAAGTCGGAATTTCATTCGCCGCTGTTGCAATTCTTGGTGCTATATTAGCTTAA

*RiAGL4*  AACAACAG---------------------CAACAGGATCCGGAAATTCTCTTAAATCTGAAATTGGAGTTTCATTCGTTGCTATTGCCGTTCTTGGTGCTATCCTCGCTTAA

*RcAGL4*  GG---------------------------CAGCATCAGCCGGAAATTCTCTTAGATCTGAAGTTGGAGTTCCATTCGCTGCCATTGCCATTCTTGGTGCTATCCTTGTCTAA

*RiAGL5*  AGGAGCAGCAG------------------CAGCATCATCCGGAAATACTCTTAAATC---ATCTGGAGTTTCATTCGCTGCCATTGCCGTTCTTGGTGCTATCTTCGCTTAA

*RcAGL5*  AGCGACAG---------------------CAGTATCGGCTGGAAATTCTCTTGAATCTGAAGCTGGAATTTCATTTGTTGCAATTGCCATCCTTGGTGCTATTTTCGGTTAA

*RcAGL6*  AACAACAGGAACAACAGGAGCAGGAGCATCAGCATCAGCCGGAAACTCTCTTAAAGCTGAAGTTGCAGTTTCATTCGCTGCCATTGCCGTTCTTGGTGCTATCTTCGCTTGA

** * * **** **** * ** * * * **** * ** ** * *** ***** * * * *

(**d**)

|  | % identity (DNA) | |
| --- | --- | --- |
| Genes | Full length  (ATG to stop) | Conserved  coding |
| *RiAGL1 : RcAGL1^a^* | 65.7 | 88.4 |
| *RiAGL2 : RcAGL2^b^* | 67.3 | nd |
| *RiAGL3 : RcAGL3^a^* | 60.1 | 84.2 |
| *RiAGL4 : RcAGL4* | 61.2 | 83.5 |
| *RiAGL4 : RcAGL5* | 51.0 | 78.2 |
| *RiAGL4 : RcAGL6* | 60.0 | 84.8 |

^a^ Putative orthologues based on DNA phylogeny of conserved coding sequence (Fig S4a,b above).

^b^ Putative orthologues based on relatively high % DNA identity, and similarity of the predicted start of the mature protein (after ER signal cleavage) including absence of N-terminal glutamine as found in the other tandemly located AGLs in both species (Fig S3, Tables S4,S5).

nd, not determined.

**Table S1** Expressed sequence tag (EST) libraries made from plant roots grown in fields, or in natural soils, or inoculated with mycorrhizas were used to generate alignments for degenerate primer design to amplify genomic AGL sequences from *R. irregularis* and *R. clarus* (see Fig. 1).

| Plant species | Abbreviation | EST collection/conditions^a^ |
| --- | --- | --- |
| *Arachis stenoperma* | As | Roots of wild peanut inoculated by juvenile *Meloidogyne arenaria* in soil (Proite *et al.*, 2007) |
| *Avena barbata* | Ab | Root, pooled from different levels of rain and nitrogen |
| *Elaeis guineensis* | Eg | Root tissue of three-month old oil palm seedlings (Ho *et al.*, 2007) |
| *Malus domestica* | Md | Roots from apple plants subjected to water stress for 1 week |
| *Medicago truncatula* | Mt | Roots harvested 3 weeks post inoculation with *Glomus intraradices* (Schenck & Smith, isolate LPA8) |
| *Oryza longistaminata* | Ol | Roots from plants grown under nitrogen-limiting conditions in soil (Yang *et al.*, 2010) |
| *Petunia hybrida* | Ph | Roots were subtracted from RNA of mycorrhizal roots (*Glomus intraradices* MUCL 43204) (Breuillin *et al.*, 2010) |
| *Saccharum* hybrid | Sh | Roots of field grown sugarcane plants (Gupta *et al.*, 2010) |

^­­a^ NCBI accession numbers of the EST sequences are as follows (with “name” in parentheses): GR364405 (Ab1); GR360928 (Ab2); EH047300 (As1); EL692929 (Eg1); GO528602 (Md1); GO564967 (Md2); GO528411 (Md3); AL387003 (Mt1); AL388047 (Mt2); AL388048 (Mt3); AL384424 (Mt4); AL385482 (Mt5); HS341901 (Ol1); FN040184 (Ph1); FN045610 (Ph2); FN041219 (Ph3); FN043344 (Ph4); FN044933 (Ph5); BI452321 (RiAGL3 (Schultz & Harrison, 2008)); DV638923 (Sh1). Three other sequences were obtained from the non-redundant database EU931681 (*RiAGL1*) and EU931682 (*RiAGL2*) (Schultz & Harrison, 2008) and AF106929 (*MtAM1*) (van Buuren et al. 1999). NCBI searches were performed in April 2013.

**References:** Breuillin *et al.*, (2010) Plant J 64:1002-1017; Gupta *et al.*, (2010) Funct Integr Genomics, 10:207-214; Ho *et al.*, (2007) BMC Genomics 8:381; Proite *et al.*, (2007) BMC Plant Biol 7:7; van Buuren *et al.*, (1999) Mol. Plant Microbe Interact. 12: 171-181; Yang *et al.*, (2010) BMC Genomics 11, 705.

**Table S2** Sequence and characteristics of degenerate primers designed to amplify genomic AGL sequences from *R. irregularis* and *R. clarus.* The primers bind to the N-terminal and C-terminal regions of *RiAGL1*, *RiAGL2* and *RiAGL3* cDNA sequences (Fig. 1).

| Primer | Direction | Sequence (5’ to 3’)^a^ | Length | Degeneracy |
| --- | --- | --- | --- | --- |
| AGL1_degF1 | Forward | CWTYMTTMTTCTYRCCRTTYTCTTCG | 26 | 256 |
| AGL1_degF2 | Forward | CYTTAGTWRCCTWCGTTGCTGC | 22 | 16 |
| AGL1_degR1 | Reverse | TTAATTTAAGCRARDATAGCDCCRA | 25 | 72 |
| AGL2_degF1 | Forward | TGGCAAAATTCWCMAAATTAAC | 22 | 4 |
| AGL2_degR1 | Reverse | TTACAAGAAGWAACCAACGAGA | 22 | 2 |
| AGL3_degF1 | Forward | AAATGAAATTYAATAAHCGYATCHTC | 26 | 36 |
| AGL3_degF2 | Forward | TCTCTTTTCYRWTAACCTTCATCA | 24 | 8 |
| AGL3_degR1 | Reverse | ARTTTAARCRAAKAWAGCWCCAAGA | 25 | 64 |

^a^ Degenerate bases are represented by the standard International Union of Pure and Applied Chemistry (IUPAC) notation.

**Table S3 Tab 1**  Summary of BLASTp and BLASTn search results showing the difficulties associated with identifying AGLs in annotated genomes. The degenerate PCR derived RiAGL and RcAGL encoded proteins and genomic DNA sequences (Fig. S1) were used as query sequences in BLASTp (non-redundant proteins (nr) database, word size =2, no filter) and BLASTn (NCBI whole-genome shotgun contigs database, word size =7, no filter) searches respectively. Organism was restricted to either *R. irregularis* or *R. clarus*, as indicated. The "top hit" results are shown for each search. see separate excel file – for full information; indicative information as image below


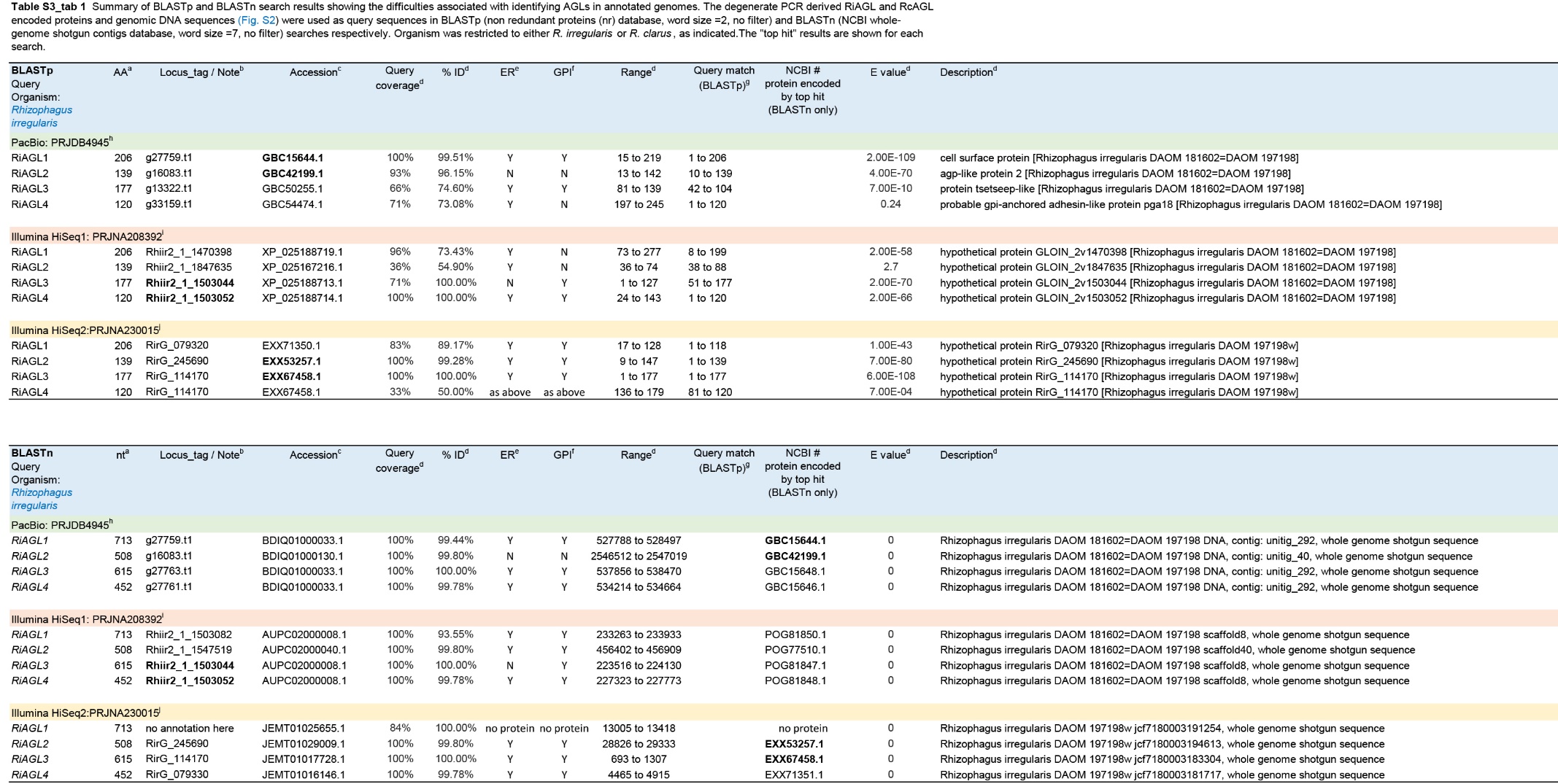


**Table S3_tab 2**  Sequences and ER and GPI-anchor signal sequence analysis of BLASTp and BLASTn top hits (see tab 1). For each query AGL, the BLASTp results are shown in columns D (NCBI accession), E (protein sequence), F (ER prediction), G (GPI prediction) and BLASTn results are provided for the encoded protein corresponding to the top nucleotide hit: columns H (NCBI accession), I (protein sequence), J (ER prediction), K (GPI prediction). The query protein sequence for each AGL (column C), see Fig S1a (*R. irregularis*) and Fig S1c (*R. clarus*) for the query nucleotide sequences. In columns E and I, green shaded boxes indicate correct or mostly correct AGL sequence identified as the top hit. Sequence matching the full length, manually annotated AGL sequences (Fig S1f,h) are underlined (ignoring, small insertions and deletions (indels) and substitutions). see separate excel file – for full information; indicative information as image below


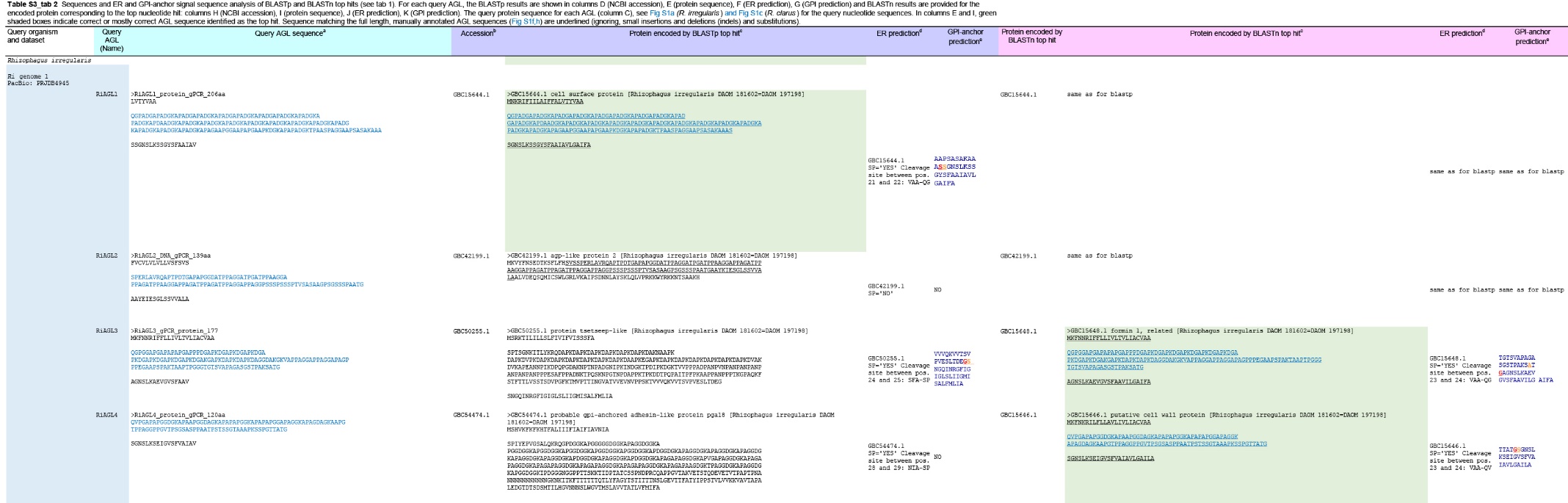


**Table S4** Genes encoding zwitterionic AGLs, *RiAGL1* and *RiAGL3* are co-located in the genome on Unitig 292, with two other full length AGL genes, *RiAGL4* (identified in this study by PCR with degenerate primers, Fig. 1, Fig. S1) and a new gene, *RiAGL5*. *RiAGL2* was identified on a different contig, Unitig 40. See Materials and Methods for prediction of ER and GPI-anchor signal sequences.


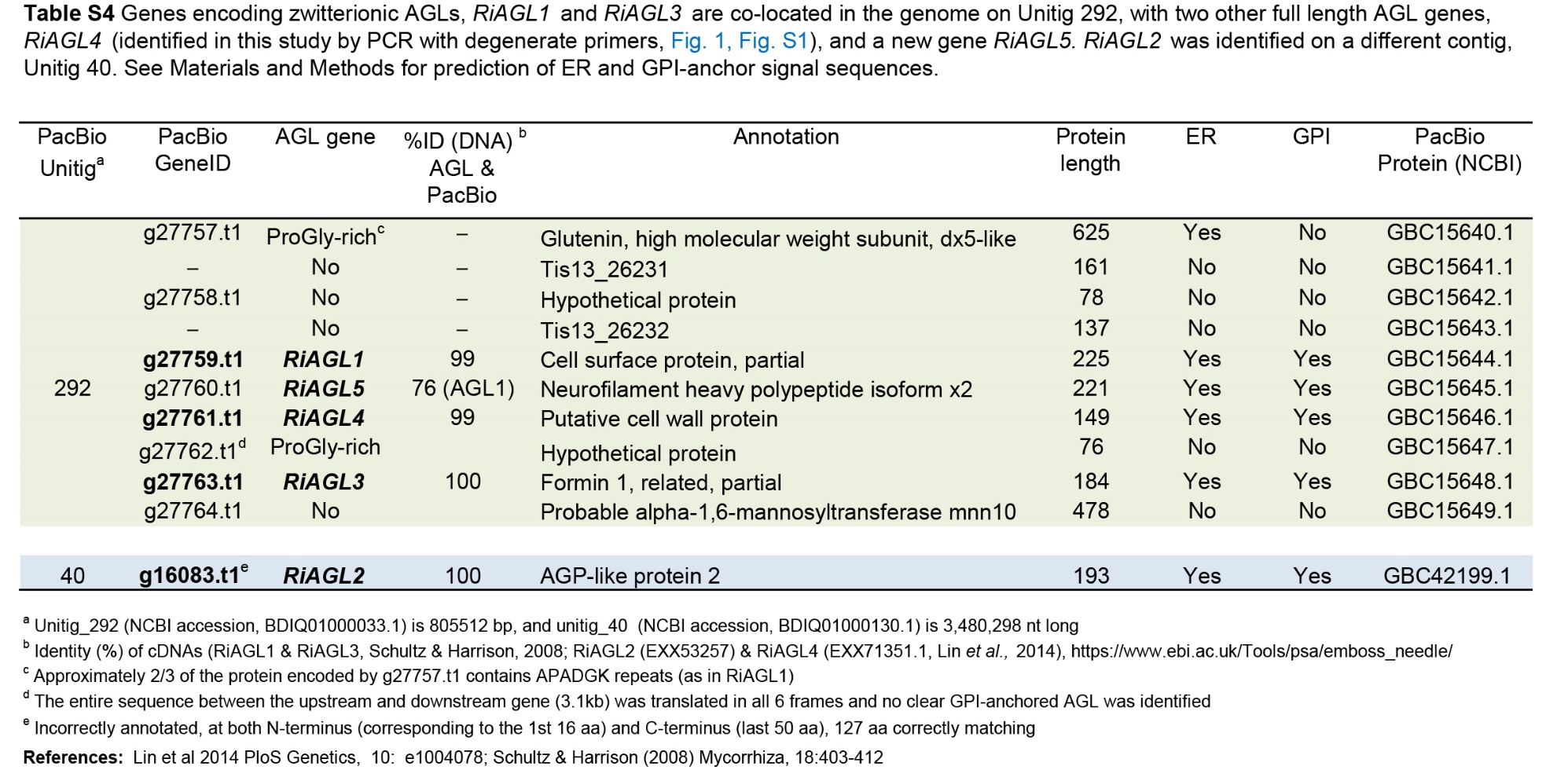


**Table S5** Most AGLs from *R. clarus* are co-located in the genome, as observed for *R. irregularis* (Table S4). See Materials and Methods for prediction of ER and GPI-anchor signal sequences.


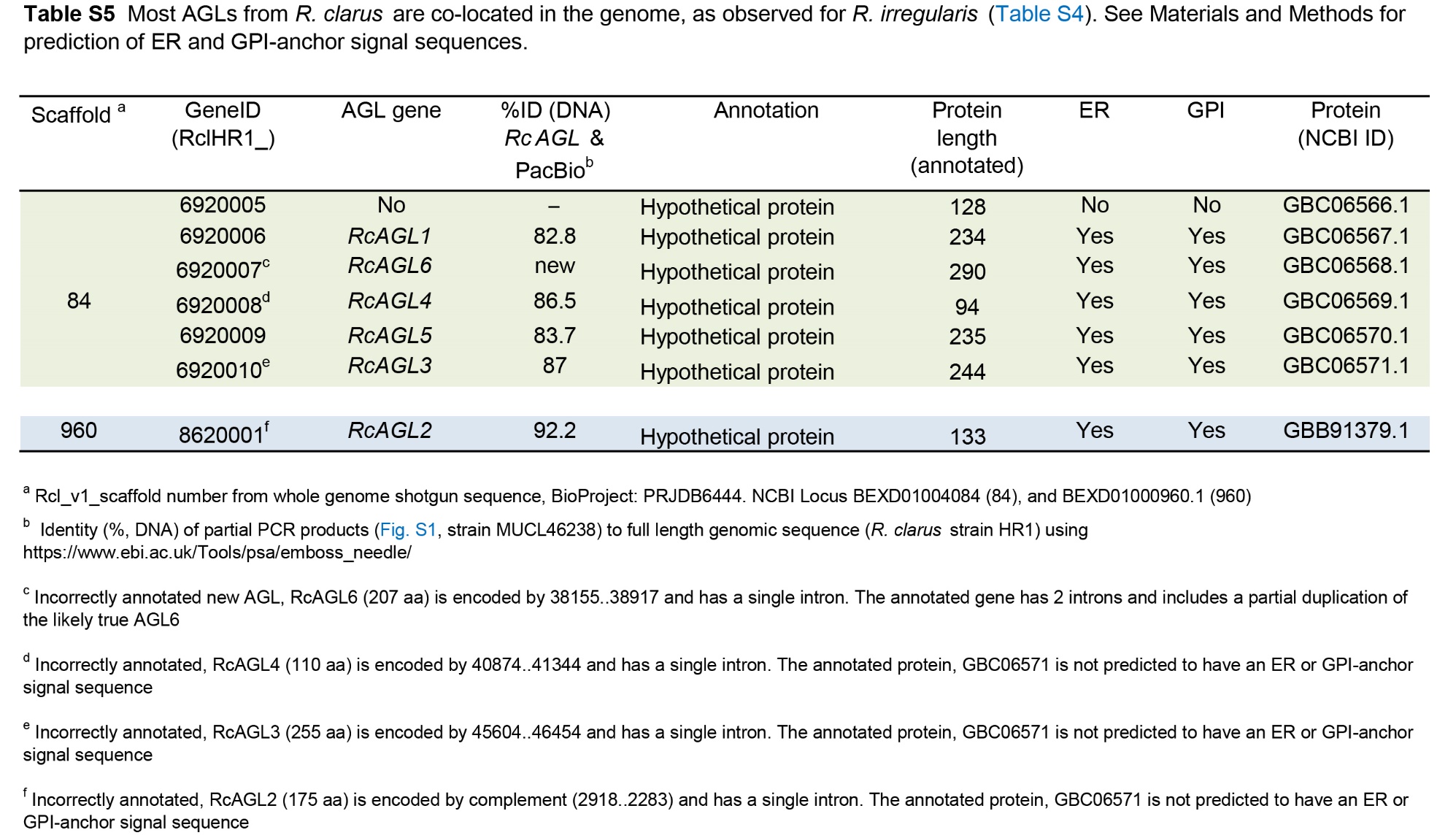


**Table S6**  Protein characteristics of the mature AGL proteins of all arbuscular mycorrhizal species analysed. This is the full set of data including % of all amino acids (to 1 decimal place) and repeats some of the information in Fig. 6. Highlighting is used to indicate the following properties: strongly basic (blue) or acidic (red) pI, individual amino acids that are present at ≥ 15% (green), aggregate PGA% ≥ 40% (yellow), and DE (acidic) ≥ 8% or KR (basic) ≥ 8% (purple). Mature proteins were obtained after removal of predicted N-terminal endoplasmic reticulum (ER) and C-terminal glycosylphosphatidyl inositol (GPI)-anchor signal sequences (Fig. S1).

see separate excel file (for full data) or Fig 5 (for the subset of data)

**Table S7** Accession numbers of AGL-like genes in *Gigaspora margarita* and *G. rosea*. AGLs were identified by NCBI-BLASTn from annotated genomes (whole-genome shotgun contigs, WGS) or transcriptomes (transcriptome shotgun assemblies). Parameters, word size =2, no filter. There was no TSA for *G. rosea*. Query sequences included all the Sccal coding sequences (ATG to stop, starting with Sccal AGL4) identified by Oases / Mira (Fig. S1). Region aligned for each WGS or TSA contig are indicated by the range on the contig (start..finish, rc, reverse complement). *G. margarita* sequences were manually annotated by aligning WGS and TSA sequences, whereas *G. rosea* WGS sequences were aligned to *G. margarita* sequences, then compared to the automatic annotation. ‘Same’ means the start, stop and intron / exon boundaries used in Fig. S1 are the same as at NCBI Jan 2021). Sequences were assessed as either full length (full) or partial sequences, and the number of introns reported. The encoded proteins were assessed for ER and GPI signal sequences (see Materials and Methods). Proteins were classified into five classes: AGL, >40% PGA, 15-39.9% PGA, Chimeric with PF10342, chimeric - other, no ER or GPI signal. Comments are provided for notable differences.

**
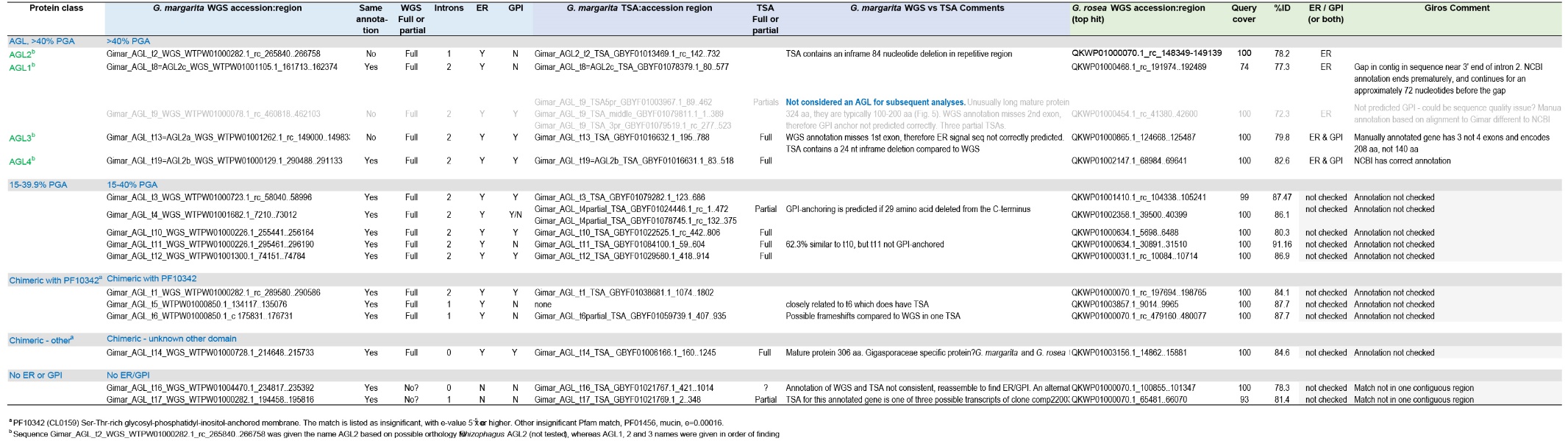
**

**Table S8** Blast searches of transcript shotgun assembly (TSA) datasets. Query sequences include *RiAGL2* coding sequence (CDS) and the best of the oases/mira cDNA sequences for the AGLs identified from *S. calospora, R. castanea* and *F. mosseae*. No hits were obtained when *RiAGL2* from *R. irregularis* was used as query. Only one TSA transcript produced a better "AGL" outcome, encoding a protein with the missing ER signal sequence (Sccal_AGL6, green shading) and two *S. calospora* and four *R. castanea* sequences produced a worse outcome (yellow shading).

see separate excel file – for full information; indicative information as image below


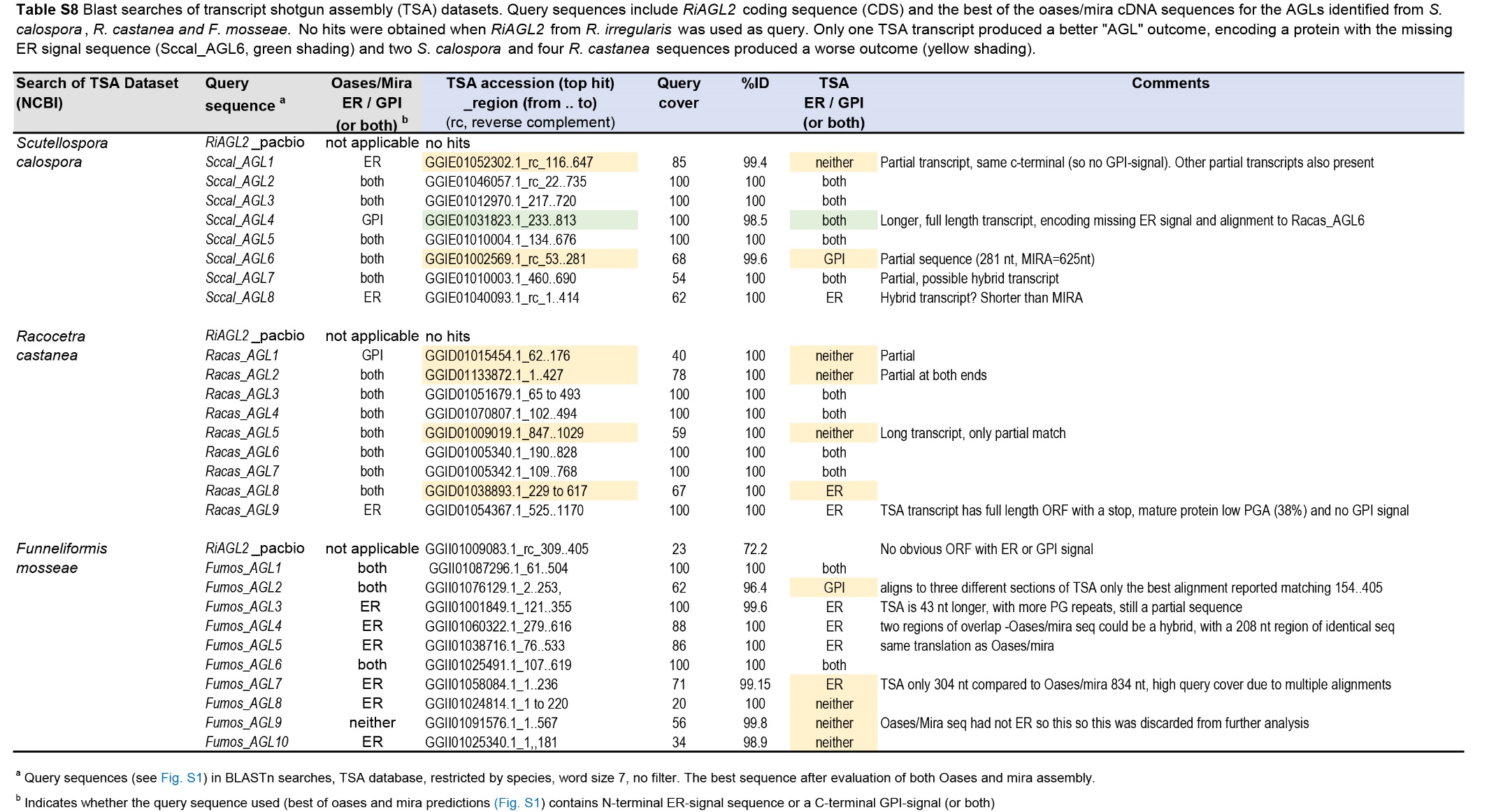


**Table S9** BLASTn searches of NCBI's non-redudtant (NR) and transcript shotgun assembly (TSA) datasets to search for AGLs/IDPs in non-arbuscular mycorrhizal fungal datasets. Query sequences include *Pabra_AGL1, Pabra_AGL2, Pabra_AGL4, RiAGL1, RiAGL2* and *RiAGL3* coding sequence (CDS). Searches were restricted by organism as follows: *Geosiphon pyriformis* (an obligate symbiont of a cyanobacteria), from the the same subphylum (Glomeromycotina, phyllum Mucoromycota) as arbuscular mycorrhizal fungi; Mortierellomycotina (phyllum Mucoromycota), Mucoromycotina subphylum (phyllum Mucoromycota), Ascomycota, Basidiomycota.

see separate excel file – for full information; indicative information as image below

**
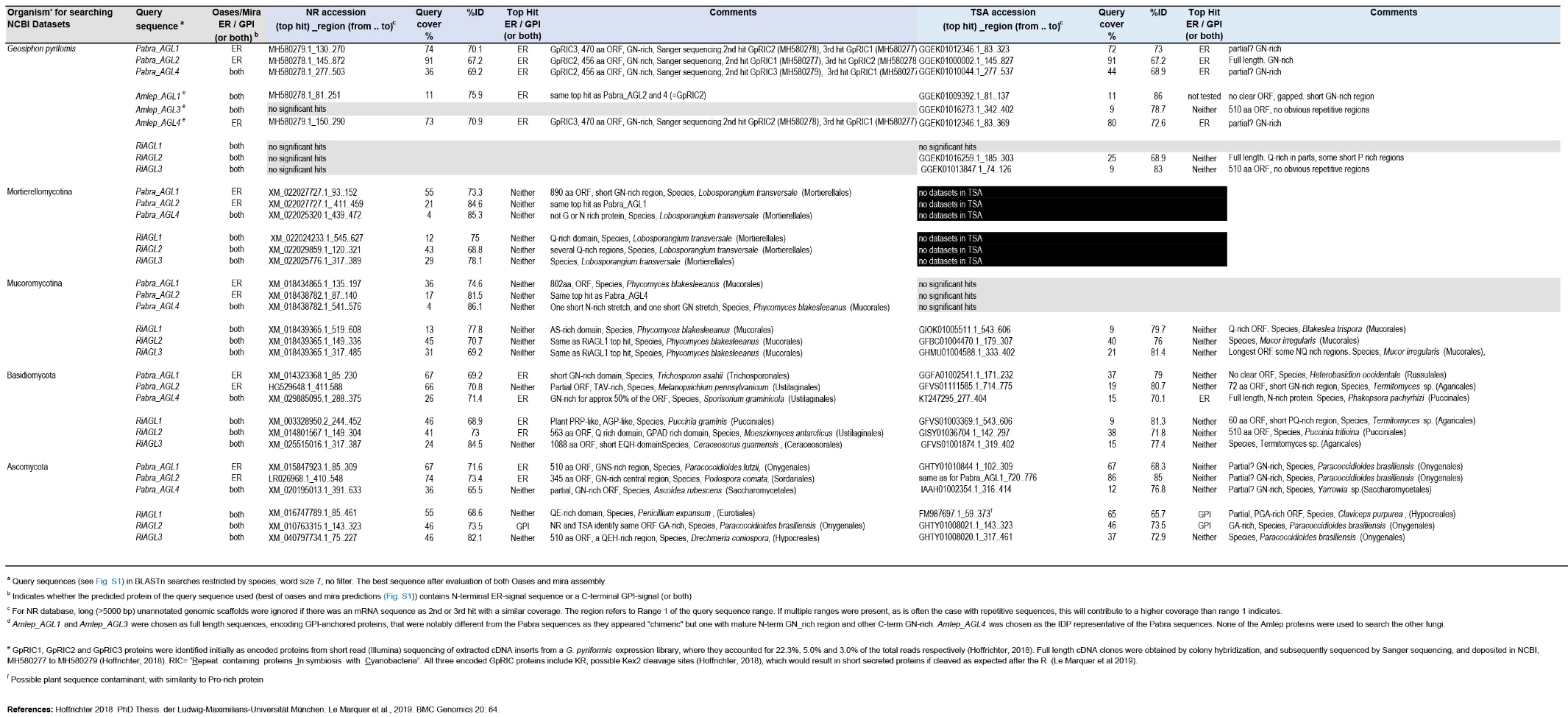
**

**Table S10** Summary of tandem repeats found in mature AGL proteins. Repeats were identified using T-REKS (Psim = 1.0, filter overlapping repeats (off)) (Jorda and Kajava, 2009). Non-overlapping tandem repeats (TR), where present, are indicated as TR1, TR2 and TR3 (eg RcAGL3). Property indicates the predicted physical property of the tandem repeat based on relative P and G content (Fig. 7) based on Rauscher *et al.* (2006). Both indicates that the protein, can be amyloid or elastic (depending on conditions).


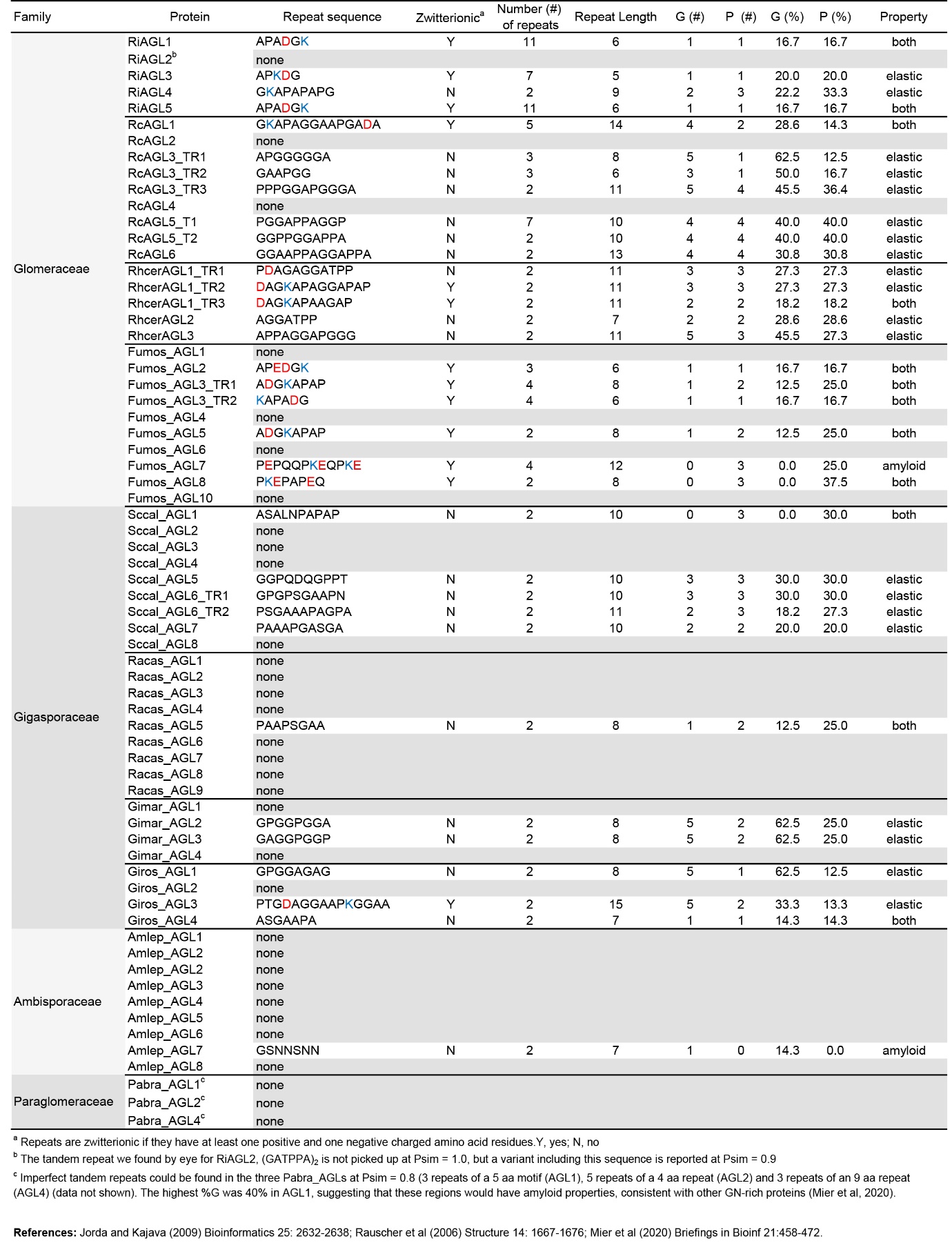

Supplement: Supplementary file 1 — Supplementary file1 (DOCX 4685 KB) [file 572_2021_1066_MOESM1_ESM.docx]
