# Supplementary material for: A targeted bioinformatics approach identifies highly variable cell surface proteins that are unique to Glomeromycotina
Source: Mycorrhiza. 2022 Jan 15;32(1):45–66. doi: 10.1007/s00572-021-01066-x (PMC8786786; doi:10.1007/s00572-021-01066-x)
Supplement: Supplementary file 2 — Supplementary file2 (DOCX 93 KB) [file 572_2021_1066_MOESM2_ESM.docx]

**Fig. S1** AGL sequences from a variety of arbuscular mycorrhizal fungi. For each species and sequence source the DNA sequences are provided first, followed by the protein translations. For DNA sequences, untranslated regions (UTRs) and introns are in lowercase text, and coding regions (uppercase). The ‘Oases’ DNA sequences have an additional 20 nucleotides (nt) of 5' and 3' UTRs to maximise the chance of getting full length sequences. Oases sequences also include, in the sequence ID line, the *k-*mer value (*k*39, *k*49, *k*59 or *k*69) of the assembly in which the sequence was found. ‘Mira’ sequences are only provided if they were clearly better than the Oases sequence. In some cases, the differences between Oases and mira are extensively annotated with comment lines starting with ‘#’ (see Sccal (Fig S1k,l)). Highlighted proteins (i–r) indicate the ones used for analysis of mature proteins. Protein translations are displayed with a line break between the predicted ER and GPI signal sequences (if present). The sequence header information incorporates the species name and information about the source of the sequence, with the following abbreviations (species first, then other information): Ri, *Rhizophagus irregularis;* Rc, *R. clarus;* Fumos, *Funneliformis mosseae; Sccal, Scutellospora calospora; Racas, Racocetra castanea; Pabra, Paraglomus brasilianum; Amlep, Ambispora leptoticha; Rhcer, R. cerebriforme; Gimar, Gigaspora margarita, Giros, Gigaspora rosea;* rc, reverse complement; pm20 = plus minus 20 nt additional 5' and 3' UTRs respectively (in lower case); The nt counts at the end of the header information are for CDS only, and amino acid (aa) counts include the signal peptides.

Sequences from the “wet-bench experiments” (Fig. S1a,c) are available from GenBank, https://www.ncbi.nlm.nih.gov, reference numbers MZ382300–MZ382308.

**References:** Beaudet *et al.,* 2018. DNA Research 25: 217-227; Kobayashi *et al.,* 2018. BMC Genomics 19; Maeda *et al.,* 2018. Communications Biology 1.

**Summary of sequences**

(a) RiAGL_gDNA_PCR products

(b) RiAGL_protein_PCR products

(c) RcAGLs_gDNA_PCR products

(d) RcAGL_protein_PCR products

(e) RiAGL_PacBio_gDNA

(f) RiAGL_PacBio_protein

(g) RcAGL_PacBio_gDNA

(h) RcAGL_PacBio_protein

(i) Fumos_oases (cDNA, then protein)

(j) Fumos_mira (cDNA, then protein)

(k) Sccal_oases (cDNA, then protein)

(l) Sccal_mira (cDNA, then protein)

(m) Racas_oases (cDNA, then protein)

(n) Racas_mira (cDNA, then protein)

(o) Amlep_oases (cDNA, then protein)

(p) Amlep_mira (cDNA, then protein)

(q) Pabra_oases (cDNA, then protein)

(r) Pabra_mira (cDNA, then protein)

(s) Gimar, NCBI BLASTn (gDNA, cDNA, then protein)

(t) Giros, NCBI BLASTn (gDNA, then protein)

(u) Rhcer, NCBI BLASTn (gDNA, then protein)

**Start of sequences**

(a) RiAGL_gDNA_PCR products

>RiAGL1_DNA

CTTTAGTTACCTACGTTGCTGCACAgtaagtttccttcacaattattattcaagaaaagaataattaatataatatggtgaagttgttaatcaaatcactcttttttatgataacagAGGACCAGCTGACGGAGCACCAGCTGACGGAAAAGCACCAGCTGACGGAGCACCAGCTGACGGAAAAGCACCAGCTGACGGAGCACCAGCCGACGGAAAAGCACCAGCTGACGGAGCACCAGCCGACGGAAAAGCACCAGCCGACGGAAAAGCACCAGCTGACGGAAAAGCACCAGACGCAGCCGACGGAAAAGCACCAGCTGACGGAAAAGCACCAGCTGACGGAAAAGCACCAGCCGACGGAAAAGCACCAGCTGACGGAAAAGCACCAGCCGACGGAAAAGCACCAGCTGACGGAAAAGCTCCAGCTGACGGAAAAGCTCCAGCTGACGGAAAAGCACCAGCTGACGGAAAAGCACCAGCTGACGGAAAAGCTCCAGCTGACGGAAAAGCTCCAGCAGGTGCTGCTCCAGGAGGTGCTGCTCCAGCTCCAGGTGCTGCTCCAAAAGACGGAAAAGCACCAGCCCCAGCTGACGGAAAAACACCAGCAGCATCACCAGCAGGAGGAGCAGCCCCATCAGCATCAGCCAAAGCAGCAGCATCATCCGGAAATTCTCTTAAATCATCTGGATATTCATTCGCTGCCATTGCCGTT

>RiAGL2_DNA

GTTCGTTTGCGTTCTTGTCCTCGTTCTCCTTGTTTCCTTCAGCGTATCATCTCCTGgttagtaaattaatccctttttacggtttcttttttggttaaaaattccttttatttaatttattcattttattttttttcttaacagAACGATTAGCTGTTCGTCAAGCACCTACACCAGATACTGGAGCTCCAGCACCAGGAGGTGATGCAACTCCACCAGCAGGTGGAGCCACACCAGGAGCAACTCCACCAGCAGCTGGAGGAGCACCACCAGCAGGAGCAACCCCACCAGCAGCTGGAGGAGCACCACCAGCAGGAGCAACCCCACCAGCAGGAGCAACTCCACCAGCAGGAGGAGCACCACCAGCAGGAGGTCCATCATCATCACCTTCTTCATCACCAACTGTGAGCGCTTCAGCTGCCGGCCCTAGTGGTAGCTCATCTCCAGCAGCAACAGGCGCTGCCTACAAAATTGAAAGTGGTTTATCTAGCGTTGTTGCCCTCGCTGC

>RiAGL3_DNA

aattataaaATGAAATTTAATAATCGTATCTTCTTTCTTCTTATTGTTCTCACCGTATTGATTGCCTGCGTTGCTGCACAgtaagttttttcctaaagaaaaaaattttaatgactgaatagataaaataaacttcttgtattgtgataatag

GGGACCAGGAGGAGCACCAGGAGCACCAGCACCAGCACCAGGAGCACCACCACCAGATGGAGCACCTAAAGATGGAGCACCCAAAGATGGAGCACCTAAAGATGGAGCACCCAAAGATGGAGCACCCAAAGATGGAGCACCCAAAGATGGAGCACCTAAAGATGGAGCCAAAGGAGCACCCAAAGATGCACCCAAAGATGCACCCAAAGATGCAGGAGGTGATGCTAAGGGTAAAGTAGCTCCGCCAGCAGGAGGAGCACCACCAGCAGGAGGAGCACCAGCAGGACCACCACCAGAAGGAGCAGCACCATCACCAGCGAAAACAGCCGCACCTACTCCAGGTGGAGGAACAGGTACATCAGTTGCTCCAGCAGGAGCATCAGGAAGCACACCTGCCAAATCAGCAACAGGAGCCGGAAACAGTCTTAAAGCCGAAGTCGGAGTTTCATTCGCAGCTGTAAT

>RiAGL4_DNA

ACAgtaagtttttttcccatgaaaaaaaaaaaaataataaataaaaaaattaataatgattgaataaataaatcctcatatttgtgataatagGGTACCAGGGGCACCAGCACCAGGAGGCGACGGAAAAGCTCCAGCAGCACCAGGAGGTGACGCGGGAAAAGCACCAGCACCAGCACCAGGCGGAAAAGCTCCAGCTCCAGCACCAGGAGGCGCTCCAGCTGGCGGTAAAGCTCCAGCAGGCGATGCAGGAAAAGCTGCCCCAGGCACTCCACCAGCAGGAGGTCCACCAGGAGTAACACCATCAGGTTCGGCATCACCACCGGCAGCAACACCATCAACATCAAGCGGGACTGCTGCCGCACCAAAATCATCACCAGGAACAACAGCAACAGGATCCGGAAATTCTCTTAAATCTGAAATTGGAGTTTCATTCGTTGCTATTGCCGTTC

(b) RiAGL_protein_PCR products

>RiAGL1_protein

LVTYVAA

QGPADGAPADGKAPADGAPADGKAPADGAPADGKAPADGAPADGKAPADGKAPADGKAPDAADGKAPADGKAPADGKAPADGKAPADGKAPADGKAPADGKAPADGKAPADGKAPADGKAPADGKAPADGKAPAGAAPGGAAPAPGAAPKDGKAPAPADGKTPAASPAGGAAPSASAKAAA

SSGNSLKSSGYSFAAIAV

>RiAGL2_protein

FVCVLVLVLLVSFSVS

SPERLAVRQAPTPDTGAPAPGGDATPPAGGATPGATPPAAGGAPPAGATPPAAGGAPPAGATPPAGATPPAGGAPPAGGPSSSPSSSPTVSASAAGPSGSSSPAATG

AAYKIESGLSSVVALA

>RiAGL3_protein

MKFNNRIFFLLIVLTVLIACVAA

QGPGGAPGAPAPAPGAPPPDGAPKDGAPKDGAPKDGAPKDGAPKDGAPKDGAPKDGAKGAPKDAPKDAPKDAGGDAKGKVAPPAGGAPPAGGAPAGPPPEGAAPSPAKTAAPTPGGGTGTSVAPAGASGSTPAKSATG

AGNSLKAEVGVSFAAV

>RiAGL4_protein

QVPGAPAPGGDGKAPAAPGGDAGKAPAPAPGGKAPAPAPGGAPAGGKAPAGDAGKAAPGTPPAGGPPGVTPSGSASPPAATPSTSSGTAAAPKSSPGTTATGSGNSLKSEIGVSFVAIAV

(c) RcAGLs_gDNA_PCR products

>RcAGL1_DNA

ACAgtaagttttttccgcgattaattattcaaaggaaaacgaaaaaataatgtaataaaaaatccctaaccacttttttttatgataacagAGCACCAGCAGGAGGAGCACCAGATGCAGGAGCAGGAGGAGCAGGAGCACCACCAGCAGGAGGAGCACCAGATGCAGGAGCAGGAGGARCAGGAGCACCACCAGCAGGAGGAGCACCAGACGCAGGAGCAGGAGGAGCAGGAGCACCAGACGCAGGAGCAGGAGGAGCAGGAGCACCACCAGCAGGAGGAAAAGCCCCAGCAGGRGGTGCCGCCCCAGGTGCTGATGCAGGAAAAGCCCCAGCAGGAGGTGCCGCCCCAGGTGCTGATGCAGGAAAAGCCCCAGCCGGAGGTGCCGCCCCAGGTGCTGATGCAGGAAAAGCCCCAGCCGGAGGTGCCGCCCCAGGTGCTGATGCAGGAAAAGCCCCAGCCGGAGGTGCCGCCCCAGGTGCTGATGCAGGAAAAGCCCCAGCAGGAGGTGCCGCCCCAGGTGCCGATGCAGGAAAAGCCCCAGCCGGAGGTGCCGCCCCAGCCCCAGGTGCCGATGCAGGAAAAGCCCCAGCCGGAGGTGCCGCTCCCCCAGCAGGAGCTGCTCCACCAGCAGGAGGTGCCACACCATCAGCAGGTACCGCAACCCCACCAGCAGGAGCATCACCAAAAGCATCAACAGGAGCAACAGCAGCATCAGCTGGAAGTTCTCTTAAATCAGAAGCTGGAGTTYCATTCGCTGCCATTGCCGTTA

>RcAGL2_DNA

GATCGTTTGCGTTCTCGTTCTCGTCCTTCTTGTTTCCTTCAGTGTTTCTGgttagtaaataagttttatttgtttcctttttttttagttaaattaattattcaatttataatttattcacatttttttttttttttttctctcaatcattaacagAACGATTAGCTGTTCGTCAAGCACCAGCACCAGCAGGAGGAGCACCACCAGCTGGAGGAGCACCACCAGCAGGAGGAGCTGGAGGAGCTGGAGGAGCCACACCACCAGCAGGAGGAGCTGGAGGAGCTACACCACCAGCAGGAGGAGCAGGAGGAGCTGGAGGAGCCACACCACCAGCAGGAGGAGCACCACCAGCAGGAGGAGATGCAGGAGCAGGAGGAGCTACACCACCAGCAGGAGGAGCACCACCAGCTGGAGGAGCACCACCAGCAGGAGGAGCAGCAGGAGGAGCATCATCAGCTGGTGCCCCAACTCCCAGTTCTTCAATGGCCAGCCCAAGTGTATCAGGAGGAGCTGCTGCAGCCCCATCAGCTTCCACTAGTGCTGCCTTCAAAGTTGAAAGTGGTTTATCCAGCATTGCTGCCCTTACCGC

>RcAGL3_DNA

TTTCTACTTACTGGTCTCGCTGTATTGATTGCCTGCGTTGCTGCACAgtaagtatttttttccttaaaaaaaaaacaaaaggtttttaatgattgtataagataataaaatctctcatttgtaataatagAGCACCAGGAGCTCCAGGAGCTCCAGGAGCACCAGGAGCACCACCAGCACCAGGAGGTGGAGGTGGAGCAGCACCAGGAGGTGGAGGTGGAGCAGCACCAGGTGGAGGAGGTGGAGCAGCACCAGGTGGAGGAGCAGCACCAGGTGGAGGAGCAGCACCAGGAGGTGGAGGTGGAGCACCAGGAGGTGGAGCCGCGGGAGGTGGAGGTGGAGGTAAGGCCAAGGTAACTCCACCAGGAGGAGCACCAGCAAAAGGAGCAGCACCAGGAGGAGGAGCACCACCAGCAGGAGGAGCAGCACCAGGAGGAGGAGCACCACCAGCGGGAGGAGCACCACCACCAGGAGGAGCACCACCACCAGGAGGAGCACCAGGAGGCGGAGCACCACCAGCAGGAGGAGCACCACCACCAGGAGGAGCACCAGGAGGAGGAGCACCACCACCAGGAGGAGCACCAGGAGGTGGAGCAACACCAGCAGGAGGAGCACCACCACCAGAAGGAGCATCACCATCACCAGCGACATCAACTCCACCTACTCCAGGTCCAGGAACTGGTACATCAGCTACTCCACCAGGAGCATCAGGAAGTGCGGCCAAAACAGCAGCAGCTGGAAACAGTCTTAAATCTGAAGTCGGAATTTCATTCGCCGCTGTTGCAAT

>RcAGL4_DNA

CTTCTTCTTACTGTTCTCGCCGTGTTAATTGCCTGCGTTGCTGCACAgtaagttatttttcttggaaaaaaaaacgtatactaactgcatgcgtataaattttataaagttgaataaataaatccctttgctttctgtgttaatagAGCACCAGCACCACCAGCTGGCGGAAAAGCCCCTACTTCAACACTACCGGGAAAAGCTCCAGGGGGTGCCACAAACACTTTAAGTCCAACAACAGGAGGCGCTCCACCAGCGGGAAGTCCACCATCAGGTTCAAAGTCGTCATCACCATCACCATCAGCATCAGCATCAGCATCAGCATCTCCATCAGGGGCAGCATCAGCCGGAAATTCTCTTAGATCTGAAGTTGGAGTTCCATTCGCTGCCATTGCCAT

>RcAGL5_DNA

ACAgtaagtgaaaaaaaaaaaaataattttttaatggataaataaataaataaacctcttgntacgggataatagGGAACCAGGAGCACCAGCAGCACCAGCAGCAGCACTAGCAAAGGCACCTGAAGGTGAAGCACCTAAAGGGGATGGCGTAAAGGTACNTAAGGGTGATGGAAAAGCACCTAAAGACGCAGGAGCACNTAAAGCAGAAGCTAAAGGCAAGGCAGGAGGTGCTCCACCAGCAAAAGGTCCACCAGGAGGTGCTCCACCAGCAGGAGGTCCACCAGGAGGTGCCCCACCAGCAGGAGGTCCACCAGGAGGCGCCCCACCAGCAGGAGGTCCACCAGGAGGCGCTCCACCAGCAGGAGGTCCACCAGGAGGCGCTCCACCAGCAGGAGGTCCACCAGGAGGTGCCCTACCAGCCGGAGGTCCACCAGGAGGTGCTCCACCAGCAGGAGGTCCACCAGGAGGCGCTCCACCAGCCGGAGGTCCACCAGGAGGTGCCCCACCAGCCGGAGGTCCACCAGGAGGTGCCCCACCAGCCGGAGGTCCACCAGGAGGTGCCCCACCAGCAGGAGGTCCAGCAGGAGGTGCTCCACCAGCAGGAGGTCCAGCAGGAGGCGCTCCACCAGCAGGAGGTCAAATAACAGAAGCGACAGCAGTATCGGCTGGAAATTCTCTTGAATCTGAAGCTGGAATTTCATTTGTTGCAATTGCCATCC

(d) RcAGL_protein_PCR products

>RcAGL1_protein

QAPAGGAPDAGAGGAGAPPAGGAPDAGAGGXGAPPAGGAPDAGAGGAGAPDAGAGGAGAPPAGGKAPAGGAAPGADAGKAPAGGAAPGADAGKAPAGGAAPGADAGKAPAGGAAPGADAGKAPAGGAAPGADAGKAPAGGAAPGADAGKAPAGGAAPAPGADAGKAPAGGAAPPAGAAPPAGGATPSAGTATPPAGASPKASTGATAASAGSSLKSEAGVXFAAIAV

>RcAGL2_protein

IVCVLVLVLLVSFSVSERLAVRQAPAPAGGAPPAGGAPPAGGAGGAGGATPPAGGAGGATPPAGGAGGAGGATPPAGGAPPAGGDAGAGGATPPAGGAPPAGGAPPAGGAAGGASSAGAPTPSSSMASPSVSGGAAAAPSASTSAAFKVESGLSSIAALT

>RcAGL3_protein

FLLTGLAVLIACVAAQAPGAPGAPGAPGAPPAPGGGGGAAPGGGGGAAPGGGGGAAPGGGAAPGGGAAPGGGGGAPGGGAAGGGGGGKAKVTPPGGAPAKGAAPGGGAPPAGGAAPGGGAPPAGGAPPPGGAPPPGGAPGGGAPPAGGAPPPGGAPGGGAPPPGGAPGGGATPAGGAPPPEGASPSPATSTPPTPGPGTGTSATPPGASGSAAKTAAAGNSLKSEVGISFAAVA

>RcAGL4_protein

LLLTVLAVLIACVAAQAPAPPAGGKAPTSTLPGKAPGGATNTLSPTTGGAPPAGSPPSGSKSSSPSPSASASASASPSGAASAGNSLRSEVGVPFAAIA

>RcAGL5_protein

QEPGAPAAPAAALAKAPEGEAPKGDGVKVXKGDGKAPKDAGAXKAEAKGKAGGAPPAKGPPGGAPPAGGPPGGAPPAGGPPGGAPPAGGPPGGAPPAGGPPGGAPPAGGPPGGALPAGGPPGGAPPAGGPPGGAPPAGGPPGGAPPAGGPPGGAPPAGGPPGGAPPAGGPAGGAPPAGGPAGGAPPAGGQITEATAVSAGNSLESEAGISFVAIAI

(e) RiAGL_PacBio_gDNA

>RiAGL1_g27759.t1_pacbio_gDNA_pm20

ATGAATAAACGCATCTTCATTATTCTCGCCATTTTCTTCGCTTTAGTTAC

CTACGTTGCTGCACAgtaagtttccttcacaattattattcaagaaaagaataattaatataatatggtg

aagttgttaatcaaatcactcttttttatgataacagAGGACCAGCTGACGGAGCACCAGCTGACGGAAA

AGCACCAGCTGACGGAGCACCAGCTGACGGAAAAGCACCAGCTGACGGAGCACCAGCCGACGGAAAAGCA

CCAGCTGACGGAGCACCAGCCGACGGAAAAGCACCAGCCGACGGAGCACCAGCCGACGGAAAAGCACCAG

ACGCAGCCGACGGAAAAGCACCAGCTGACGGAAAAGCACCAGCTGACGGAAAAGCACCAGCCGACGGAAA

AGCACCAGCTGACGGAAAAGCACCAGCCGACGGAAAAGCACCAGCTGACGGAAAAGCTCCAGCTGACGGA

AAAGCTCCAGCTGACGGAAAAGCACCAGCTGACGGAAAAGCACCAGCTGACGGAAAAGCTCCAGCTGACG

GAAAAGCTCCAGCAGGTGCTGCTCCAGGAGGTGCTGCTCCAGCTCCAGGTGCTGCTCCAAAAGACGGAAA

AGCACCAGCCCCAGCTGACGGAAAAACACCAGCAGCATCACCAGCAGGAGGAGCAGCCCCATCAGCATCA

GCCAAAGCAGCAGCATCATCCGGAAATTCTCTTAAATCATCTGGATATTCATTCGCTGCCATTGCCGTTC

TTGGAGCTATCTTCGCTTAA

>RiAGL2_g16083.me_pacbio_gDNA_pm20

ATGGCAAAATTCAC

AAAATTAACGTTCGTTTGCGTTCTTGTCCTCGTTCTCCTTGTTTCCTTCAGCGTATCATCTCCTGgttag

taaattaatccctttttacggtttcttttttggttaaaaattccttttatttaatttattcattttattt

tttttcttaacagAACGATTAGCTGTTCGTCAAGCACCTACACCAGATACTGGAGCTCCAGCACCAGGAG

GTGATGCAACTCCACCAGCAGGTGGAGCCACACCAGGAGCAACTCCACCAGCAGCTGGAGGAGCACCACC

AGCAGGAGCAACCCCACCAGCAGCTGGAGGAGCACCACCAGCAGGAGCAACCCCACCAGCAGGAGCAACT

CCACCAGCAGGAGGAGCACCACCAGCAGGAGGTCCATCATCATCACCTTCTTCATCACCAACTGTGAGCG

CTTCAGCTGCCGGCCCTAGTGGTAGCTCATCTCCAGCAGCAACAGGCGCTGCCTACAAAATTGAAAGTGG

TTTATCTAGCGTTGTTGCCCTCGCTGCTCTCGTTGGTTATTTCTTGTAA

>RiAGL3_g27763.t1_pacbio_gDNA_pm20

ATGAAATTTAATAATCGTATCTTCTTTCTTCTTATTGTTCTCACCGTATT

GATTGCCTGCGTTGCTGCACAgtaagttttttcctaaagaaaaaaattttaatgactgaatagataaaat

aaacttcttgtattgtgataatagGGGACCAGGAGGAGCACCAGGAGCACCAGCACCAGCACCAGGAGCA

CCACCACCAGATGGAGCACCTAAAGATGGAGCACCCAAAGATGGAGCACCTAAAGATGGAGCACCCAAAG

ATGGAGCACCCAAAGATGGAGCACCCAAAGATGGAGCACCTAAAGATGGAGCCAAAGGAGCACCCAAAGA

TGCACCCAAAGATGCACCCAAAGATGCAGGAGGTGATGCTAAGGGTAAAGTAGCTCCGCCAGCAGGAGGA

GCACCACCAGCAGGAGGAGCACCAGCAGGACCACCACCAGAAGGAGCAGCACCATCACCAGCGAAAACAG

CCGCACCTACTCCAGGTGGAGGAACAGGTACATCAGTTGCTCCAGCAGGAGCATCAGGAAGCACACCTGC

CAAATCAGCAACAGGAGCCGGAAACAGTCTTAAAGCCGAAGTCGGAGTTTCATTCGCAGCTGTAATTCTT

GGTGCTATATTTGCTTAA

>RiAGL4_g27761.t1_pacbio_gDNA_pm20

ATGAAATTCAATAAACGCATCCTCTTTCTTCTTGCTGTTCTCATTGTATT

GATTGCCTGCGTTGCTGCACAgtaagtttttttcccatgaaaaaaaaaaaataataaataaaaaaattaa

taatgattgaataaataaatcctcatatttgtgataatagGGTACCAGGGGCACCAGCACCAGGAGGCGA

CGGAAAAGCTCCAGCAGCACCAGGAGGTGACGCGGGAAAAGCACCAGCACCAGCACCAGGCGGAAAAGCT

CCAGCTCCAGCACCAGGAGGCGCTCCAGCTGGCGGTAAAGCTCCAGCAGGCGATGCAGGAAAAGCTGCCC

CAGGCACTCCACCAGCAGGAGGTCCACCAGGAGTAACACCATCAGGTTCGGCATCACCACCGGCAGCAAC

ACCATCAACATCAAGCGGGACTGCTGCCGCACCAAAATCATCACCAGGAACAACAGCAACAGGATCCGGA

AATTCTCTTAAATCTGAAATTGGAGTTTCATTCGTTGCTATTGCCGTTCTTGGTGCTATCCTCGCTTAA

>RiAGL5_g27760.t1_pacbio_gDNA_pm20

ATGAAATTCACTAAACGTACCTTCTTTCTTCTTGCTGTTCTCACTTTGTT

GATTGCCTGTGTTGCTGCACAgtaagtttttttttttccctgaaaaaaaaaaataataataaatttttta

atagtttaataaataaaaaatatttcttgtttatgataatagAGGACCAGCTGACGGAAAAGCTCCAGCT

GACGGAAAAGCTCCAGCTGACGGAAAAGCTCCAGCTGATGGAAAAGCTCCAGCCGACGGAAAAGCCCCAG

CTGACGGTGCTGCTGACGGAAAAGCCCCAGCCGACGGAAAAGCCCCAGCTGACGGAAAAGCTCCAGCTGA

CGGAAAAGCTCCAGCTGATGGAAAAGCTCCAGCTGATGGAAAAGCTCCAGCTGACGGAAAAGCTCCAGCT

GACGGAAAAGCCCCAGCCGACGGAAAAGCCCCAGCTGATGGAAAAGCTCCAGCTGATGGAAAAGCTCCAG

CTGACGGAAAAGCTCCAGGTGCTGCTCCAGGTGGTGCTCCAGCTCCAGGTGCTGCTCCAGGTGCTGCTCC

AGGTGCTGCTCCAGCAGGAGCTCCACCAGCAGGAGGTCCAAAAGGAGTAACACCAGCAGGCACAGCAACA

CCACCACCACCACCACCAAACAGTGCTGCTTCCGCACCAAAAACATCATCAACAGGAGGAGCAGCAGCAG

CATCATCCGGAAATACTCTTAAATCATCTGGAGTTTCATTCGCTGCCATTGCCGTTCTTGGTGCTATCTT

CGCTTAA

(f) RiAGL_PacBio_protein

>RiAGL1_g27759.t1_pacbio

MNKRIFIILAIFFALVTYVAA

QGPADGAPADGKAPADGAPADGKAPADGAPADGKAPADG

APADGKAPADGAPADGKAPDAADGKAPADGKAPADGKAPADGKAPADGKAPADGKAPADG

KAPADGKAPADGKAPADGKAPADGKAPADGKAPAGAAPGGAAPAPGAAPKDGKAPAPADG

KTPAASPAGGAAPSASAKAAAS

SGNSLKSSGYSFAAIAVLGAIFA

>RiAGL2_g16083.me_pacbio

MAKFTKLTFVCVLVLVLLVSFSVS

SPERLAVRQAPTPDTGAPAPGGDATPPAGGATPGAT

PPAAGGAPPAGATPPAAGGAPPAGATPPAGATPPAGGAPPAGGPSSSPSSSPTVSASAAG

PSGSSSPAATG

AAYKIESGLSSVVALAALVGYFL

>RiAGL3_g27763.t1_pacbio

MKFNNRIFFLLIVLTVLIACVAA

QGPGGAPGAPAPAPGAPPPDGAPKDGAPKDGAPKDGA

PKDGAPKDGAPKDGAPKDGAKGAPKDAPKDAPKDAGGDAKGKVAPPAGGAPPAGGAPAGP

PPEGAAPSPAKTAAPTPGGGTGTSVAPAGASGSTPAKSATG

AGNSLKAEVGVSFAAVILGAIFA

>RiAGL4_g27761.t1_pacbio

MKFNKRILFLLAVLIVLIACVAA

QVPGAPAPGGDGKAPAAPGGDAGKAPAPAPGGKAPAP

APGGAPAGGKAPAGDAGKAAPGTPPAGGPPGVTPSGSASPPAATPSTSSGTAAAPKSSPG

TTATG

SGNSLKSEIGVSFVAIAVLGAILA

>RiAGL5_g27760.t1_pacbio

MKFTKRTFFLLAVLTLLIACVAA

QGPADGKAPADGKAPADGKAPADGKAPADGKAPADGA

ADGKAPADGKAPADGKAPADGKAPADGKAPADGKAPADGKAPADGKAPADGKAPADGKAP

ADGKAPADGKAPGAAPGGAPAPGAAPGAAPGAAPAGAPPAGGPKGVTPAGTATPPPPPPN

SAASAPKTSSTGGAAAAS

SGNTLKSSGVSFAAIAVLGAIFA

(g) RcAGL_PacBio_gDNA

>RcAGL1_v1_6920006_pacbio_gDNA_pm20

ATGAATAAACGCATTATCTTTCTTCTCGCCGTTCTCTTAGCTTTAGTAAC

TTATGTTGCTGCACAgtaagttttttccgcgattaattattcaaaggaaaacgaaaaaataatgtaataa

aaaatccctaaccacttttttttatgataacagAGCACCAGCAGGAGGAGCACCAGATGCAGGAGCAGGA

GCACCACCAGCAGGAGGAGCACCAGATGCAGGAGCAGGAGCAGGAGGAGCACCACCAGCACCAGACGCAG

GAGCAGGAGGAGCAGGAGCACCAGACGCAGGAGCAGGAGGAGCAGGAGCACCACCAGCAGGAGGAAAAGC

CCCAGCAGGAGGTGCCGCCCCAGGTGCTGATGCAGGAAAAGCCCCAGCAGGAGGTGCCGCCCCAGGTGCT

GATGCAGGAAAAGCCCCAGCCGGAGGTGCCGCCCCAGGTGCTGATGCAGGAAAAGCCCCAGCCGGAGGTG

CCGCCCCAGGTGCTGATGCAGGAAAAGCCCCAGCAGGAGGTGCCGCCCCAGGTGCCGATGCAGGAAAAGC

CCCAGCCGGAGGTGCCGCCCCAGCCCCAGGTGCCGATGCAGGAAAAGCCCCAGCCGGAGGTGCCGCTCCC

CCAGCAGGAGCTGCTCCACCAGCAGGAGGTGCCACACCATCAGCAGGTACCGCAACCCCACCAGCAGGAG

CATCACCAAAAGCATCAACAGGAGCAACAGCAGCATCAGCTGGAAGTTCTCTTAAATCAGAAGCTGGAGT

TTCATTCGCTGCCATTGCCGTTATTGCTGCTATCCTCGCTTAA

>RcAGL2_v1_18620001_pacbio_gDNA_pm20

ATG

GCCAAATTCTCAAAATTAACGATCGTTTGCGTTCTCGTTCTCGTCCTTCTTGTTTCCTTC

AGTGTTTCTGgttagtaaataagttttatttgtttcctttttttttagttaaattaatta

ttattcaatttataatttattcacattttttttttttttttctctcaatcattaacagAA

CGATTAGCTGTTCGTCAAGCACCAGCACCAGCAGGAGGAGCACCACCAGCTGGAGGAGCA

CCACCAGCAGGAGGAGCTGGAGGAGCTGGAGGAGCCACACCACCAGCAGGAGGAGCTGGA

GGAGCTACACCACCAGCAGGAGGAGCAGGAGGAGCTGGAGGAGCCACACCACCAGCAGGA

GGAGCACCACCAGCAGGAGGAGATGCAGGAGCAGGAGGAGCTACACCACCAGCAGGAGGA

GCACCACCAGCTGGAGGAGCACCACCAGCAGGAGGAGCAGCAGGAGGAGCATCATCAGCT

GGTGCCCCAACTCCCAGTTCTTCAATGGCCAGCCCAAGTGCATCAGGAGGAGCTGCTGCA

GCCCCATCAGCTTCCACTAGTGCTGCCTTCAAAGTTGAAAGTGGTTTATCCAGCATTGCT

GCCCTTACCGCTCTCGTTGGTTTCTTCTTGTAA

>RcAGL3_v1_69200010me_pacbio_gDNA_pm20

ATGAAATTTAATAAACGTATCTTCTTTCTACTTACTGGTCTCGCTGTAT

TGATTGCCTGCGTTGCTGCACAgtaagtatttttttccttaaaaaaaaaacaaaaggtttttaatgattg

tataagataataaaatctctcatttgtaataatagAGCACCAGGAGCACCAGGAGCACCAGGAGCACCAG

GAGCACCACCAGCACCAGGAGGTGGAGGTGGAGCAGCACCAGGAGGTGGAGGTGGAGCAGCACCAGGTGG

AGGAGGTGGAGCAGCACCAGGTGGAGGAGCAGCACCAGGAGGTGGAGGTGGAGCACCAGGAGGTGGAGCC

GCGGGAGGTGGAGGTGGAGGTAAGGCCAAGGTAACTCCACCAGGAGGAGCACCAGCAAAAGGAGCAGCAC

CAGGAGGAGGAGCACCACCAGCAGGAGGAGCAGCACCAGGAGGAGGAGCAGCACCAGGAGGAGGAGCAGC

ACCAGGAGGAGGAGCACCACCAGCGGGAGGAGCACCACCACCAGGAGGAGCACCACCACCAGGAGGAGCA

CCAGGAGGCGGAGCACCACCACCAGGAGGAGCACCAGGAGGCGGAGCACCACCAGCAGGAGGAGCACCAC

CACCAGGAGGAGCACCAGGAGGTGGAGCAACACCAGCAGGAGGAGCACCACCACCAGAAGGAGCATCACC

ATCACCAGCGACATCAACTCCACCTACTCCAGGTCCAGGAACTGGTACATCAGCTACTCCACCAGGAGCA

TCAGGAAGTGCAGCCAAAACAACAGCAGCTGGAAACAGTCTTAAATCTGAAGTCGGAATTTCATTCGCCG

CTGTTGCAATTCTTGGTGCTATATTAGCTTAA

>RcAGL4_v1_6920006_pacbio_gDNA_pm20

ATGG

CATTCAATAAACGCATCATCCTTCTTCTTACTGTTCTCGCCGTGTTAATTGCCTGCGTTGCTGCACAgta

agttatttttcttggaaaaaaaacgtatactaactgcatgcgtataaattttataaagttgaataaataa

atccctttgctttctgtgttaatagAGCACCAGCACCACCAGCTGGCGGAAAAGCCCCTACTTCAACACT

ACCGGGAAAAGCTCCAGGGGGTGCCACAAACACTTTAAGTCCAACAACAGGAGGCGCTCCACCAGCGGGA

AGTCCACCATCAGGTTCAACGTCGTCATCACCATCAGCATCAGCATCAGCATCTCCATCAGGGGCAGCAT

CAGCCGGAAATTCTCTTAGATCTGAAGTTGGAGTTCCATTCGCTGCCATTGCCATTCTTGGTGCTATCCT

TGTCTAA

>RcAGL5_v1_6920009_pacbio_gDNA_pm20

ATGAAATTCAATAAACGTATCTTATTTCTTATTGTCTTCACTGTGTTGTT

TATCTATGTTGCTGCACAgtaagtggaaaaaaaaaaaaataattttttaatggataaataaataaataaa

cctcttgctacgggataatagGGAACCAGGAGCACCAGCAGCACCAGCAGCAGCACTAGCAAAGGCACCT

GAAGGTGAAGCACCTAAAGGGGATGGCGTAAAGGTACCTAAGGGTGATGGAAAAGCACCTAAAGACGCAG

GAGCACCTAAAGCAGAAGCTAAAGGCAAGGCTGGAGGTGCTCCACCAGCAAAAGGTCCACCAGGAGGTGC

TCCACCAGCAGGAGGTCCACCAGGAGGTGCCCCACCAGCAGGAGGTCCACCAGGAGGCGCCCCACCAGCA

GGAGGTCCACCAGGAGGCGCCCCACCAGCAGGAGGTCCACCAGGAGGCGCTCCACCAGCAGGAGGTCCAC

CAGGAGGCGCTCCACCAGCAGGAGGTCCACCAGGAGGTGCTCCACCAGCCGGAGGTCCACCAGGAGGCGC

TCCACCAGCAGGAGGCGCTCCACCAGCAGGTGCCCCACCAGCAGGAGGTCCACCAGGAGGCGCTCCACCA

GCAGGGGGTCCACCAGGAGGTGCCCCACCAGCCGGAGGTCCAGCAGGAGGCGCTCCACCAGCAGGAGGTC

AAATAACAGAAGCGACAGCAGTATCGGCTGGAAATTCTCTTGAATCTGAAGCTGGAATTTCATTTGTTGC

AATTGCCATCCTTGGTGCTATTTTCGGTTAA

>RcAGL6_v1_6920007me_pacbio_gDNA_pm20

ATGAAATTCAATAAACGTACTTTCTTTCTTCTTGCTGTCTTCACTGTGTT

GATTGCTTGCGTTGCTGCACAgtaagttttttcttcctgaaaaaaaaaaacaaataaataaaataaataa

aaaatattttgaatagataaataaataattctttttgtttatgataatagAG

CACCAGCACCAGCACCAGGTGATGCAGGAAAAGCCGCTCCAGCGGGAGGTGCCGCACCACCAGCAGGAGG

AAAAGCCGCTCCAGATGCAGGAAAAGGTGCTCCACCAGCAGGAGGTGCCGCTCCAGATGCAGGAAAAGCT

CCAGCGGGAGGCGCTGCCCCACCAGCAGGAGGAAAAGCCCCAGCAGATGCAGGAAAAGCCCCAGCAGGAG

GTGCTGCCCCACCAGCAGGAGGTGCTCCACCAGCAGGAGGTGCTGCCCCACCAGCAGGAGGTGCTCCACC

AGCAGGAGGTGCTCCACCAGCAGGAGGCGCTGCCCCACCAGCAGGAGGTGCTCCAGCTGACGCTCCACCA

GCAGGAGGTCCAGCACCAGTTACACCAGCAGGTACAGCAACACCACCAACACCACCACCATCTGCTGGTG

CACCCTCACCATCAACAGGAACAACAGGAACAACAGGAGCAGGAGCATCAGCATCAGCCGGAAACTCTCT

TAAAGCTGAAGTTGCAGTTTCATTCGCTGCCATTGCCGTTCTTGGTGCTATCTTCGCTTGA

(h) RcAGL_PacBio_protein

>RcAGL1_v1_6920006_pacbio

MNKRIIFLLAVLLALVTYVAA

QAPAGGAPDAGAGAPPAGGAPDAGAGAGGAPPAPDAGAG

GAGAPDAGAGGAGAPPAGGKAPAGGAAPGADAGKAPAGGAAPGADAGKAPAGGAAPGADA

GKAPAGGAAPGADAGKAPAGGAAPGADAGKAPAGGAAPAPGADAGKAPAGGAAPPAGAAP

PAGGATPSAGTATPPAGASPKASTGATAAS

AGSSLKSEAGVSFAAIAVIAAILA

>RcAGL2_v1_18620001_pacbio

MAKFSKLTIVCVLVLVLLVSFSVS

ERLAVRQAPAPAGGAPPAGGAPPAGGAGGAGGATPP

AGGAGGATPPAGGAGGAGGATPPAGGAPPAGGDAGAGGATPPAGGAPPAGGAPPAGGAAG

GASSAGAPTPSSSMASPSASGGAAAAPSASTS

AAFKVESGLSSIAALTALVGFFL

>RcAGL3_v1_69200010me_pacbio

MKFNKRIFFLLTGLAVLIACVAA

QAPGAPGAPGAPGAPPAPGGGGGAAPGGGGGAAPGGG

GGAAPGGGAAPGGGGGAPGGGAAGGGGGGKAKVTPPGGAPAKGAAPGGGAPPAGGAAPGG

GAAPGGGAAPGGGAPPAGGAPPPGGAPPPGGAPGGGAPPPGGAPGGGAPPAGGAPPPGGA

PGGGATPAGGAPPPEGASPSPATSTPPTPGPGTGTSATPPGASGSAAKTTA

AGNSLKSEVGISFAAVAILGAILA

>RcAGL4_v1_6920006_pacbio

MAFNKRIILLLTVLAVLIACVAA

QAPAPPAGGKAPTSTLPGKAPGGATNTLSPTTGGAPP

AGSPPSGSTSSSPSASASASPSGAAS

AGNSLRSEVGVPFAAIAILGAILV

>RcAGL5_v1_6920009_pacbio

MKFNKRILFLIVFTVLFIYVAA

QEPGAPAAPAAALAKAPEGEAPKGDGVKVPKGDGKAPK

DAGAPKAEAKGKAGGAPPAKGPPGGAPPAGGPPGGAPPAGGPPGGAPPAGGPPGGAPPAG

GPPGGAPPAGGPPGGAPPAGGPPGGAPPAGGPPGGAPPAGGAPPAGAPPAGGPPGGAPPA

GGPPGGAPPAGGPAGGAPPAGGQITEATAVS

AGNSLESEAGISFVAIAILGAIFG

>RcAGL6_v1_6920007me_pacbio_cDNA_pm20

MKFNKRTFFLLAVFTVLIACVAA

QAPAPAPGDAGKAAPAGGAAPPAGGKAAPDAGKGAP

AGGAAPDAGKAPAGGAAPPAGGKAPADAGKAPAGGAAPPAGGAPPAGGAAPPAGGAPPAG

GAPPAGGAAPPAGGAPADAPPAGGPAPVTPAGTATPPTPPPSAGAPSPSTGTTGTTGAGA

SAS

AGNSLKAEVAVSFAAIAVLGAIFA

(i) Fumos_oases (DNA, then protein)

>Fumos_AGL1_bRiAGL1_k39_L8944_T1of1_444nt

ctttatttaatcaatcgaaa

ATGTCTAAATTCACCAAATTAGTCATCTGCGCC

ATTGTCGCCCTCCTCATAATTGGCACGGCTTTTGCTGGAGAAGCACGCCT

TGTAGCTCGTCAAGACGAACCCTCCGCAACACCATCAGGTGACGCAACAC

CAGACGCAACACCAGTTGCAACACCAGTTGACGGTAAAGCACCAACTGAC

GGAAAAGCACCAAATCTTCCATCATCTCCAGCCCCCTCAGGCTCAGTCCC

AATCGCATCAAAAATTGCTGATGCCTTTAGCTCTGCTACAGCCGGAGCAA

GCATGTACCCACAACCAACCGGTGCACCAAAATACCCAACTACCGGCTCC

GGCTCCAGCTCTACCACCCCCGCCACAACTCCAAAGTCAGGTGCCGCCAA

GATCGAAAGTGGTTTATTCAGTGTTGCCGCAATCGCTTCCTTTGGTTTAT

TCTTCTTGTAA

aggattaattagtattttat

>Fumos_AGL2_bRiAGL1_k59_L1164_L2097_405nt

cagttcaataatcatacaaa

ATGAAATTCAATAACCGCATCATC

CTTCTTCTTATCGTTCTCTTCGTGTTGGTTTCATTCGTTGCTGCACAAGA

TCCAGAAGATGGAAAAGCACCAGCTGATGGAAAAGCACCAGAAGACGGAA

AAGCACCAGCTGATGGAAAAGCACCAGAAGACGGCAAAGCACCAGAAGAC

GGCAAAGCACCAGAAGACGGCAAAGCA

CCAGCTGACGGTAAAGCC

CCAGCCGACGGAAAAGCCCCAACAGGAAAAGGCCCAGATGGTAAAGCACC

AACACCAGCCAAGACCCCCGAGGCCAAATCCCCAGCACCTTCTGCCAAAG

CCGCCGCTGCCGCTGCCAACAATCTTAAATCCGAAGTTGGAGTTACATTC

GCAGCCATCGCTGTTCTTGGTGCCTTATTTGCTTAA

tttaataccgtttagactta

>Fumos_AGL3part_bRiAGL1_k59_L1374rc_T7of7_235nt

cttttaatacctaatataaa

ATGGCAAAA

TTCTCAAAATTGACTGTTCTCTTCATTGTTGCTATCCTCATCATTGGAACTCTCGCTTCC

TTTGGAGCCACAGAAAATTCTCGTTTAGTTGCTCGTCAAGACCCAGCACCAGCTGACGGT

AAAGCACCAGCACCAGCTGACGGAAAAGCACCAGCACCAGCTGACGGTAAAGCACCAGCA

CCAGCTGACGGTAAAGCACCAGCACCAGCTGACGGTAAAGCACCAG

>Fumos_AGL4_bRiAGL3_k69_L5911rc_T1of1_615nt

cgcagagtacattccatata

ATGGCGTCAACACGACGCATCTCGATATATCT

TTTACTCCTACTTGTCGCTTTATTCCTTGTGAGTGTTGCTTTTCCTGTTGCAAATGCACA

AGACCCAAAAGCACCAGACGATGCAAAACAACCAGAAACTAAACAACCAGAGAAAGCACC

AGAAGACGCTGCACCAAAGGAACCAAAACAAGAGGCGCCCAAAGATCTTCCACCTGATGA

CCAGCCAAAGGACCAACCAAAGGAACAGCCAAAGGAACAACCAAAGGTAGTATCAACGCA

GAGTACATTCCATATAATGGCGTCAACACGACGCATCTCGATATATCTTTTACTCCTACT

TGTCGCTTTATTCCTTGTGAGTGTTGCTTTTCCTGTTGCAAATGCACAAGACCCAAAAGC

ACCAGACGATGCAAAACAACCAGAAACTAAACAACCAGAGAAAGCACCAGAAGACGCTGC

ACCAAAGGAACCAAAACAAGAGGCGCCCAAAGATCTTCCACCTGTAAGTTTGTTCTGTAT

TATTTCAAAAAAAAATTTTTTTTCTTCTCTATTTAATCTTTCAAAAAACATTTGGAGAAA

CTTATTTTTAATTAAAGCCAACTTTGCAAACGTGGTTATTTGA

atagtatattta

>Fumos_AGL5_bRiAGL5_k59_L2159_T7of10_k49_L2758_T4of10_531nt

aaaaaaaaccaacttatatc

ATGTCTCGTTTTATAACTTGTTTGACGCTGCTTCTTGCTATTGTTACCTTGGCCT

CTGCCCAGGTCAAGACGATAAATACTCCAAATGGAGACATTACACAAGGAGCGACTGTTA

AAATCACTTGGGAACTAATGGCACCAGTGAATACTTTAGGAAACTTACGAGCCGTTAACA

AAGCTACGCAAGGTTCAACTACAATATCAGATACTTTAGATCTGAATGCGTTATCACTTG

ACTGGGTGGTTAATGTAGACCCGGGTAATTATAACTTCGCCCTTAACGATGGAAGTGGAG

ATAAATATTCTGGTCCATTTAATGTAGTAGCACCAAAAACACCTGCCCCTGCTGATGGCA

AAGCTCCTGCTGATGGCAAAGCTCCTGCTCCTGCTGACGGAAAAGCTCCTGCTCCTGCCG

ATGGCAAAGCTCCAGCGGCTAAAGATCCAGCTAATAAAACACC

GCTGAAGAAAAATCGAAGAGCAAACCCTCAGCTTCAGGGAATATT

TTTCAAGCATTGGACAATAAACCTCTAA

gtgataatgtccacagtaac

>Fumos_AGL6_bRiAGL1_k59_L4177_T1of1_513nt

caaacaaaataataatcaaa

ATGAACAAAGCTATTACCGTCTTTTTATTTCTCGCTGGTC

TCATTGCCTTAACTCAAGCTGCGGAACACTTAGTTAACGTTGGTACCAAA

GATGGAAAAAATTTGTTCGAACCTGACACCGTAAATGCCGCCATGGGTGA

TACCGTCAAATTCGTTTGGGTTTCGGGTAAACACAACGTCATCGAATCTG

ATGCAAAGGGAGCTTGTACCGCATCAAAAGCTCCAAATGCTTTCACTACC

GAAATATACGAAGCTCCTAAAGAATGGGTCCTTGACCTTAAAGATGTTAC

TACTCCCAAGAAATGGTTCTACTGCAGTGTTGGTCAACATTGCGCAAATG

GTATGACCGGTACTATTCTCATTGGTGGCGACAACAAAACTGCACCCGCT

GATGGTAAAACACCTGACGGAAAAGCCCCCGCTTCTGCCGCTGCTCAAAC

CTATACTTCTGCTTATGCTGTCACCGCTGGAGTCGTATTCTCAAGCATGT

GCATGGCCGCTTATATGTTATAA

gtgctttcttatgactaaat

>Fumos_AGL7_FumosAGL4_k69_L8818_T1of3_834nt

aattttcaaatagactcaag

ATGTATT

ATGTTGAACTTAAATACAAGCACCTTTATTTACTTATCATATTTCTTATT

GCGGTACTTATTGCCGTTAATGGTGCATCTCCAACATATGAAATAAAGGA

TTCGATTAACTTTCTTCGAAAACGACAAGATCCTGAACCACAACAACCGA

AAGAGCAACCGAAGGAGCCGGAACCACAACAACCGAAAGAGCAACCAAAG

GAGCCGGAACCACAACAACCGAAGGAGCAACCGAAAGAGCCTGAACCACA

ACAACCGAAAGAGCAACCGAAAGAGCCTGAAAAGAGCCTGAACGAGCCTG

AACAAAAGAGCCTGAACCACAACAGCCGAAGGAGCAACCGAAGGAGCAAC

CGAAAGAGCCTAAAAGAGCCTGAACCACAACAACCGAAAGAGCCTGAACC

ACAACAGCCGAAGGAGCAACCAAAAGAGCCTGAACCACAACCGAAAGAGC

CTGAGCCACAACAACCGAAGGAGCAACCGAAAGAGCCTGAGCCGCAACAA

CCGAAGGAGCAACCGAAAGAGCCTGAACCACAACAACCGAAAGAGCAACC

GACGAAAGAGCCTGAACCACAACAGAAAGAGCAACCGAAGGAAGAACCTG

AGTCTGTAGCACCGAAAGAAGCACCAAAAGATCCTGAGCCAAAAGTTCCT

GAACCACCAAAGCAAACCTGCGCTTCTCCTGATGATCCTGGATGCAAACC

TCCATCAGGAGTTACTCCGAAAGTTGAGACATCTATGCAAACATCAATAG

AAACATTAATGCCAACTCCAGATAATGATGATGGGAATAATGGGAATAAT

GGGAATAATGATGGGAATAATGAGTAG

caccgaaagaagcaccaaaa

>Fumos_AGL8_bFumosAGL8part_k49_L1444rc_T5of7_525nt

taatctcaattatattcaag

ATGATTTATGACAAAACTACATATAAGCGAC

TTTGTTTAATAATCATAGTTCTTATTGCGGTATTTATTGCCGTCAATTATGCACATCCAA

CTCTTGAACGGAAGAATTCAGCAAGCATTCTTCGAAAACGACAAGATCCAAAATCTCCAA

AAGAGCCGGCACCTGAACAACCGAAAGAGCCTGCACCTGAACAACCGAAAGAGCCTGAAC

CCGAATCACCAAAATCACCAAAAGAGCCTGCACCTGAACAACCTAAAGAGCCTGAACCAA

AAGAGCCTAAATCACCTAAATCACCAAAAACACCTGAATCACCTAAATCACCAAAAGAGC

CAGAACCTACTTGTGTTGAAGATGATGAAACTACATGTCCACCAACCGAGTCCGATTGCG

TTGAAGATGATGAAACTACATGTCCAGAAGAACCTAAAGAACCCGAGTCCGTTTGCGTTA

AAAATGATGGAACTATATGTACAGAACCTAAAGAACCAGAGCCCGACTGCGATGAACCTA

AATGTCTTGTCTAA

atgtccaaatactttaggtt

>Fumos_AGL9_bFumosAGL8_k69_L4757rc_T1of1_1059nt

catctctattttcattcaac

ATGACCGATCAAGACACTACTACTACAAGTACCACTAATCCTGAGGTATCAGCC

AGCAGTCAGTCAGTCCCTGAAACGAATGCTGAAGTTAAAAAAACTGAAGTGAAAACCGAG

GAACTTCACGAGGTTCCCAAAATTAAACCTGAAGTACTTAAAACAGAAGAGCTTAAAAAA

GAAGACCCCCCAAAAGAACCTAAAATAGAAGAGGTCAAAAAAGAACAAGTAAAACAGCCA

GAGCCGATTAGCGCTGCTATTGTTGCAGAAGAGAAACCAGTAGAGAAACCTAAAGAGAAT

GCTGAAGCAACTAAGAGTGTTGAACCTAAACTTGAAGAAACTAAAGTTGTAGAACCAACC

ATTGAAGAACAATCCAAAAAGGAACCAGCTAATAAAGAACCACCTAAACCAACCACAAAG

GAACCAACTAATGAGGAACCACCTAAACAAACCACGAAGGAACCAACTAATGAGGAACCA

CCTAAGCCAACCACAAAGGAACCAACTAATGAGGAACCACCTAAACTAACCACAAAGGAA

CCACCTAAACCAACTACAGAGGAACCACCTAAACCAACCACAGAGGAACCACCTAAACCA

ACCACAGTGGAACCACCTAAACCAACTACAGAGGAACAACCTAAACCAACCACAGAGGAA

CCACCTAAACCAACCACAGAGGAACCACCTAAACCAACCACAAAGGAACCAGCTAATGAA

CCAACTAAAGATGAACCAAAGAAAGATGAACCAAAACAAGTTGAACAAAATCAAGAACAG

AAAGAGGATCCTAAAGTTAATGCAGTAGAAAAGAAAACTGAAGATGAATCAACAAAGGTA

GTTGAAGAACCAAGTAAAGCAAGCACCCCTGAATCTAATCCACCCGTTGTTCCACCTAAA

GCTGAAGATAAAACCGCTGAAAATGATGCTCCTGAAGTTCCACCTAAAAGTACGGAAACT

ACCGAATCAATTAACAAGCGGAAGAGTTTTGTTAAAAAATTAAAAGATTACTTTGGCTTT

AAAAGTCCTAAAGATACTAAAGTTTCAGACAGAGGAACCACCTAA

accaaccacaaaggaaccag

>Fumos_AGL10_bFumosAGL8_k59_L2818rc_T9of16_528nt

catacgag

ATGACGTCGAGTCTCGTGGGCTCGGAGATGTGTATAAGAGACAGGTATCAAC

GCAGAGTCATTTATTCTTTTTTTTTATTCTTTGTTTTTTCCTCAAAAATGAAATTATCTA

TAATTACATTTCTTTCAATTTTCCTTTCATTATTCGTAGTTACAATAGCCGAACCCATTC

ATAAAGTCAGACGTTTATCAGTATTTCCAGTGTCAATAGCCTTACCAGAATCAGATGGAC

CTATAGCAAATGATCCAGAAAAACCGGAAAAACAACCAGAACAAGAAGCACCAGAACAAC

CAAAAGAAAGTAATCCACAGGAACAACCAGATGAAGATCCGCAAGAACAACCAAAAGGAA

GTGATCCAGAAGAAGAGTCACCAAATCCAGTTGAACCTCCATCATCATCACCAACAAGTG

ATTCAAAAGGAATTCCTGTAGAAACTCCCCAAAACAATTCAGACGGAACTCCTGTAGAAA

CGCCCCCCACCACACCAGGCGGAACTCTGTTAATGTTTGATAACAATATAATTTGA

aagtgtccgcttcaagtact

>Fumos_AGL1_bRiAGL1_k39_ L8944_T1of1_147aa

MSKFTKLVICAIVALLIIGTA

FAGEARLVARQDEPSATPSGDATPDATPVATPVDGKAPTDGKAPNLPSSPAPSGSVPIAS

KIADAFSSATAGASMYPQPTGAPKYPTTGSGSSSTTPATTPKSG

AAKIESGLFSVAAIASFGLFFL

>FumosAGL2_bRiAGL1_k59_L1164_L2097_405nt

MKFNNRIILLLIVLFVLVSFVAA

QDPEDGKAPADGKAPEDGKAPADGKAPEDGKAPEDGKAPEDGKAPADGKAPADGKAPTGKGPDGKAPTPAKTPEAKSPAPSAKAAA

AAANNLKSEVGVTFAAIAVLGALFA

>Fumos_AGL3part_bRiAGL1_k59_L1374rc_T7of7_78aa

MAKFSKLTVLFIVAILIIGTLA

SFGATENSRLVARQDPAPADGKAPAPADGKAPAPADGKAPAPADGKAPAPADGKAP

>Fumos_AGL4_bRiAGL3_k69_L5911rc_T1of1_204aa

MASTRRISIYLLLLLVALFLVSVAFPVANA

QDPKAPDDAKQPETKQPEKAPEDAAPKEPKQEAPKDLPPDDQPKDQPKEQPKEQPKVVSTQSTFHIMASTRRISIYLLLLLVALFLVSVAFPVANAQDPKAPDDAKQPETKQPEKAPEDAAPKEPKQEAPKDLPPVSLFCIISKKNFFSSLFNLSKNIWRNLFLIKANFANVVI

>Fumos_AGL5_bRiAGL5_k59_L2159_T7of10_k49_L2758_T4of10_176aa

MSRFITCLTLLLAIVTLASA

QVKTINTPNGDITQGATVKITWELMAPVNTLGNLRAVNKATQGSTTISDTLDLNALSLDWVVNVDPGNYNFALNDGSGDKYSGPFNVVAPKTPAPADGKAPADGKAPAPADGKAPAPADGKAPAAKDPANKTPLKKNRRANPQLQGIFFKHWTINL

>Fumos_AGL6_bRiAGL1_k59_L4177_T1of1_513nt

MNKAITVFLFLAGLIALTQA

AEHLVNVGTKDGKNLFEPDTVNAAMGDTVKFVWVSGKHNVIESDAKGACTASKAPNAFTTEIYEAPKEWVLDLKDVTTPKKWFYCSVGQHCANGMTGTILIGGDNKTAPADGKTPDGKAPASA

AAQTYTSAYAVTAGVVFSSMCMAAYML

>Fumos_AGL7_FumosAGL4_k69_L8818_T1of3_277aa

MYYVELKYKHLYLLIIFLIAVLIAVNG

ASPTYEIKDSINFLRKRQDPEPQQPKEQPKEPEPQQPKEQPKEPEPQQPKEQPKEPEPQQPKEQPKEPEKSLNEPEQKSLNHNSRRSNRRSNRKSLKEPEPQQPKEPEPQQPKEQPKEPEPQPKEPEPQQPKEQPKEPEPQQPKEQPKEPEPQQPKEQPTKEPEPQQKEQPKEEPESVAPKEAPKDPEPKVPEPPKQTCASPDDPGCKPPSGVTPKVETSMQTSIETLMPTPDNDDGNNGNNGNNDGNNE

>Fumos_AGL8_bFumosAGL8part_k49_L1444rc_T5of7_174aa

MIYDKTTYKRLCLIIIVLIAVFIAVNYA

HPTLERKNSASILRKRQDPKSPKEPAPEQPKEPAPEQPKEPEPESPKSPKEPAPEQPKEPEPKEPKSPKSPKTPESPKSPKEPEPTCVEDDETTCPPTESDCVEDDETTCPEEPKEPESVCVKNDGTICTEPKEPEPDCDEPKCLV

>Fumos_AGL9_bFumosAGL8_k69_L4757rc_T1of1_352aa

MTDQDTTTTSTTNPEVSASSQSVPETNAEVKKTEVKTEELHEVPKIKPEVLKTEELKKEDPPKEPKIEEVKKEQVKQPEPISAAIVAEEKPVEKPKENAEATKSVEPKLEETKVVEPTIEEQSKKEPANKEPPKPTTKEPTNEEPPKQTTKEPTNEEPPKPTTKEPTNEEPPKLTTKEPPKPTTEEPPKPTTEEPPKPTTVEPPKPTTEEQPKPTTEEPPKPTTEEPPKPTTKEPANEPTKDEPKKDEPKQVEQNQEQKEDPKVNAVEKKTEDESTKVVEEPSKASTPESNPPVVPPKAEDKTAENDAPEVPPKSTETTESINKRKSFVKKLKDYFGFKSPKDTKVSDRGTT

>Fumos_AGL10_bFumosAGL8_k59_L2818rc_T9of16_175aa

MTSSLVGSEMCIRDRYQRRVIYSFFLFFVFSSKMKLSIITFLSIFLSLFVVTIAEPIHKVRRLSVFPVSIALPESDGPIANDPEKPEKQPEQEAPEQPKESNPQEQPDEDPQEQPKGSDPEEESPNPVEPPSSSPTSDSKGIPVETPQNNSDGTPVETPPTTPGGTLLMFDNNII

(j) Fumos_mira (DNA, then protein)

# CDS same as OASES

# xx ambiguities in MIRA sequence – use OASES

# other comments

# Fumos_AGL1_mira - two overlapping contigs gives the same CDS same as OASES

>Fumos_AGL1_mira_c270_23reads_c105_37reads_444nt

# Fumos_AGL2_mira two contigs 464 and 574 reads, but difficult to resolve repeats regions

# use OASES

>Fumos_AGL2_mira_c2_464reads_c5_574reads

# Fumos_AGL3part_mira sequence is 99 nt longer but still not full length (no stop)

>Fumos_AGL3part_mira_c8_179reads_334nt

ATGGCAAAATTCTCAAAATTGACTGTTCTCTTCATTGTTGCTATCCTCATCATT

GGAACTCTCGCTTCCTTTGGAGCCACAGAAAATTCTCGTTTAGTTGCTCGTCAAGACCCA

GCACCAGCTGACGGTAAAGCACCAGCACCAGCTGACGGAAAAGCACCAGCACCAGCTGAC

GGTAAAGCACCAGCACCAGCTGACGGTAAAGCACCAGCACCAGCTGACGGTAAAGCACCA

GCACCAGCTGACGGTAAAGCACCAGCTGACGGAAAAGCACCAGCTGACGGTAAAGCACCA

GCTGACGGAAAAGCACCAGCTGACGGTAAAGCACCAACTG

# Fumos_AGL4_mira three contigs, few reads (42, 4, 17 reads), difficult to resolve

# use OASES

>Fumos_AGL4_mira_c98_c1066_192

# Fumos_AGL5_mira sequence has a different C-terminus, includes stop codon, but

# still does not encode a GPI-anchored protein

>Fumos_AGL5_mira_c17rc_76reads_c4_328reads_537nt

ATGTCTCGTTTTA

TAACTTGTTTGACGCTGCTTCTTGCTATTGTTACCTTGGCCTCTGCCCAGGTCAAGACGA

TAAATACTCCAAATGGAGACATTACACAAGGAGCGACTGTTAAAATCACTTGGGAACTAA

TGGCACCAGTGAATACTTTAGGAAACTTACGAGCCGTTAACAAAGCTACGCAAGGTTCAA

CTACAAT

ATCAGATACTTTAGATCTGAATGCGTTATCACTTGACTGGGTGGT

TAATGTAGACCCGGGTAATTATAACTTCGCCCTTAACGATGGAAGTGGAG

ATAAATATTCTGGTCCATTTAATGTAGTAGCACCAAAAACACCTGCCCCT

GCTGATGGCAAAGCTCCTGCTGATGGCAAAGCTCCTGCTCCTGCTGACGG

AAAAGCTCCTGCTCCTGCCGATGGCAAAGCTCCAGCGGCTAAAGATCCAG

CTAATAAAACACCCAATAAAACACCCGCTGCTGAACCAAGTCAACCTCCA

AAAGCCCCAGCGGAACCAAATGAACCAAAAAATCCAAAGTAA

# Fumos_AGL6_mira sequence is missing first 7 nt of the CDS, go with oases sequence

>Fumos_AGL6_mira_c32_209reads

# Fumos_AGL7part_mira sequence is longer at C-term, but shorter than oases due to

# elimination of a large region of the repeat sequences

>Fumos_AGL7part_mira_c94rc_137reads_647nt

ATGTATTATGTTGAAC

TTAAATACAAGCACCTTTATTTACTTATCATATTTCTTATTGCGGTACTTATTGCCGTTA

ATGGTGCATCTCCAACATATGAAATAAAGGATTCGATTAACTTTCTTCGAAAACGACAAG

ATCCTGAACCACAACAACCGAAAGAGCCTGAACCACAACAGCCGAAGGAGCAACCAAAAG

AGCCTGAACCACAACCGAAAGAGCCTGAGCCACAACAACCGAAGGAGCAACCGAAAGAGC

CTGAGCCGCAACAACCGAAGGAGCAACCGAAAGAGCCTGAACCACAACAACCGAAAGAGC

AACCGAAAGAGCCTGAACCACAACAACCGAAAGAGCAACCGAAAGAACCTGAGTCTGTAG

CACCGAAAGAAGCACCAAAAGATCCTGAGCCAAAAGTTCCTGAACCACCAAAGCAAACCT

GCGCTTCTCCTGATGATCCTGGATGCAAACCTCCATCAGGAGTTACTCCGAAAGTTGAGA

CATCTATGCAAACATCAATAGAAACATTAATGCCAACTCCAGATAATGATGATGGGAATA

ATGGGAATAATGGGAATAATGATGGGAATAATGATGGGAATAATGGGAATAATAATGGGA

ATAATGATGGGAATAATGATGGGAATAATGA

# Fumos_AGL8_mira CDS same as OASES

>Fumos_AGL8_mira_c29_450reads

# Fumos_AGL9_mira, three overlapping contigs of 21, 15 and 97 reads

# same N-terminus as oases, and still no ER signal – discard

>Fumos_AGL9_mira_c115_208_c97

# Fumos_AGL10_mira sequence uses one of the “internal” Met (ATG) of oases seq

# and this encodes an ER-signal sequence so use mira, also has a stop codon

>Fumos_AGL10_mira_c1_318reads_414nt

ATGAAATTATCTATAATTACATTTCTTTCAATTTTCCTTTCA

TTATTCGTAGTTACAATAGCCGAACCCATTCATAAAGTCAGACGTTTATC

AGTATTTCCAGTGTCAATAGCCTTACCAGAATCAGATGGACCTATAGCAA

ATGATCCAGAAAAACCGGAAAAACAACCAGAACAAGAAGCACCAGAACAA

CCAAAAGAAAGTAATCCACAGGAACAACCAGATGAAGATCCGCAAGAACA

ACCAAAAGGAAGTGATCCAGAAGAAGAGTCACCAAATCCAGTTGAACCTC

CATCATCATCACCAACAAGTGATTCAAAAGGAATTCCTGTAGAAACTCCC

CAAAACAATTCAGACGGAACTCCTGTAGAAACGCCCCCCACCACACCAGG

CGGAACTCAGGTAATTAATTAA

# Fumos_AGL3part_mira sequence is 99 nt (33 aa) longer, partial sequence

# still not full length (no stop)

>Fumos_AGL3part_mira_c8_179reads_111aa

MAKFSKLTVLFIVAILIIGTLA

SFGATENSRLVARQDPAPADGKAPAPADGKAPAPADGKAPAPADGKAPAPADGKAPAPADGKAPADGKAPADGKAPADGKAPADGKAPT

>Fumos_AGL5_mira_c17rc_76reads_c4_328reads_178aa

MSRFITCLTLLLAIVTLASA

QVKTINTPNGDITQGATVKITWELMAPVNTLGNLRAVNKATQGSTTISDTLDLNALSLDWVVNVDPGNYNFALNDGSGDKYSGPFNVVAPKTPAPADGKAPADGKAPAPADGKAPAPADGKAPAAKDPANKTPNKTPAAEPSQPPKAPAEPNEPKNPK-

>Fumos_AGL7part_mira_c94rc_137reads_215aa

MYYVELKYKHLYLLIIFLIAVLIAVNG

ASPTYEIKDSINFLRKRQDPEPQQPKEPEPQQPKEQPKEPEPQPKEPEPQQPKEQPKEPEPQQPKEQPKEPEPQQPKEQPKEPEPQQPKEQPKEPESVAPKEAPKDPEPKVPEPPKQTCASPDDPGCKPPSGVTPKVETSMQTSIETLMPTPDNDDGNNGNNGNNDGNNDGNNGNNNGNNDGNNDGNN

# Fumos_AGL9_mira, three overlapping contigs of 21, 15 and 97 reads

# same N-terminus as oases, and still no ER signal – **discarded**

>Fumos_AGL9_mira_c115_208_c97

# Fumos_AGL10_mira sequence uses one of the “internal” Met (ATG) of oases seq

# and this encodes an ER-signal sequence so use this

# also has a stop codon

>Fumos_AGL10_mira_c1_318reads_137aa

MKLSIITFLSIFLSLFVVTIA

EPIHKVRRLSVFPVSIALPESDGPIANDPEKPEKQPEQEAPEQPKESNPQEQPDEDPQEQPKGSDPEEESPNPVEPPSSSPTSDSKGIPVETPQNNSDGTPVETPPTTPGGTQVIN

(k) Sccal _oases (DNA, then protein)

>Sccal_AGL1_bgiros2b_k49_L6948_T3_3_624nt

ggttcacattctaatacgaa

ATGAATATGACTAAAA

ATACAATCATATTCTTTGTTAGGTGTATTTTTTTCATCTCTAGTATTCTTGCTCAAGCTC

CAGGTCCGGCCTTTAGTCCGGCTCCGGCTCCAGCTTCAGCTCTAAATCCAGCTCCAGCTC

CAGCTTCAGCTCTAAATCCAGCTCCAGCTCCAGCTCCAGTTCCAAATCCAAACTTTTCAC

GAGTCCCATTCCCAGCCCCTTCACCAGCCCCAACACCAACTCCGGCTCCATTTCCAGCTA

AAGCCCCCACTCTACCTCCAGCCTCATTTCTAGCCCCTGTTTCAGCCTTTTCTCTAGCCT

CTGATTTAGCCCCAATTTCAACTCCGGACCTTTCTCTAGCCTCTGATCTAGCTTCTGCCTg

CTGCTCCAGCCCTTATTTCAGATACGTCAGCCCCAACTTCAGAACCAGCACCAGTTATAA

CACAGCCTCCGCCACCCAGTGGTTCTAACAAAAATCTAGCGTCTAGTGGTTCAGCACCAA

TTCCGAATTCTACTGGACCTGTATCTACAAAATTTATACCCCCTTCTATATCTGGCGTTC

ATATCAACTATTTAGAATTGATTATTGGACCTTTGGTTGCTCTACTCATTGGTGTTTTAT

TATATTAA

gtttggatacagccaacact

>Sccal_AGL2_bracas_f_k49_L3437_T2of2_516nt

tataacaaattataataatc

ATGTCACG

ACTTGTTTTTCTTACGCTACTCTTGGTATTTATTGGGTTTACATCAGCCC

AGGTTTCGTCAGTTAATACTCCCGGTCCTAAAATTGCTGTTGGTGCCACT

GTCCCCATCACTTGGACATACACCCCACAAGTGAATGCGCTACCAGGAAC

TTTAAGTGTTATCGATAGCGTAACTCAAAGCTCAACTATAATTAGCAGTT

CAATAGTCCTCTCTAATAAAAATTACCAGTGGGTAGTCAACGTTCCTGCG

GGGAGTTATTACCTTGGTCTCAACGATGGGTCTGGTAATAAACTCTCCGG

AGTTTTCCAAGTCTTTGACCCCAATTCTTCATCACCTGCTGCTAATCCTG

GATCACCTGCAGCTCCTGCTCCCTCTGGTGGTTCTGCTTCTAATTCCTCT

TCCCCTTCCACAACTTCCCAAAAATCAATTGCTGCTTCCTTTACTGTACC

AATCAATCTTTTATTTAACCTTATTGTGGTTGCTGCGGTAATGGTATACT

TTGTTTAA

aattgattaacctaggaaca

>Sccal_AGL3_bracas_g_k49_L1_T5777of30356_504nt

aatttgaataaaataaaata

ATGTCGCGACTTATTTTTCTTAC

GATATTCTTGGTTTTTATTGGTTTCACTTCGGCCCAGGTTACCTCGGTTA

AAGCAGCTCCTTCTAGTGTTATAGTTGGTTCCCCCGTTACATGTACTTGG

AACTACACTGGGGCAGCGGCCACAGGCAATTTAAGTCTTGTCGATGAAAC

AACTAAAGCCGCAACTGTAATTAGCTCCGCAATAAACCTCACAAGTCTAA

GTTACTCATATCAGACTACTACCCCGGGCGATTTTTGGTTTAGTTTAAGT

GATGGGACTAGTAATTCGCTCTCTAACGTCTTTCATGTCTTGACTCCCGC

ACAAGCTAGTGCTAGTTCCACCGGAGCTCCATCTCCCTCTGACACATCTT

CCCCCAATGGGGGATCTCCCGGTTCATCAGCCAAAAAAAGTGCTGCTTCG

GCTAAATCAAGCTTCACTGTATCAAATAAACTTCTGATTGGTCTTATTAT

GATTGTAACAGTAATGATGTTCTTTGCCTAA

aattattattttagaaaatt

>Sccal_AGL4_bracas_h_k59_L1738_T2_part_256nt

ACCTTGCTCTCAATGATGGATCTGGTAATAAGGACACTGGAACTTTCACA

GTTTTTAATCCTGCTGGTGCTGCACCCGCACCTGCACCTGCTGCTTCAGC

TGCTCCAGCTGCATCTGGCACCGCCCCGGCTGCAACTAGCGCCGCTCCGG

CTGCACCTCGCCCACCACCATCTGGTGCTCCAGCTCAATCAGCTCCAGCT

CCAGCTCAAGCTCCCGCCGCTTCTCAAGGTCCTGCTCAACCTCAAGCATC

TGCTAA

atctacatcccctagttcagctgcaccgtaa

gtatttaa

atttcaacataaacctt

>Sccal_AGL5_bracas_a_k59_L5940_T1_543nt

cacattttttttaagacgaa

ATGAATATTGCTAGAAATATAATTATCTTTCTCATCGTAT

GCATTCTTATCTCTGGTGTTCTTGCTCAGGGGGGTTCACCATCTAATAGC

CCTGGTGGTCCTCAAGGTCCACCAACTGGTGGTCCTCAAGGTCCTCGACC

TGGTGGTCCTCAATCTGGTGGTCCTCAAGATCAAGGTCCTCCAACTGGTG

GTCCTCAAGATCAAGGTCCTCCAACTGGTGGTCCTCCAACTGGTGGTTCT

CCAACTGGTGGTCCTCAAGATCAAGGTCCTCCAACTGGTGCTCCAACTGG

TGCTCCTCAAGGCCCTTCTGGAACTGCGGGTCCTGCGGGTCCTCAAGGTA

CTCCTGGAACTGCAGGTTCTCCTCAAACTGGTCCTACCCCGACTGGTAGT

CATGCTGGCTCTTCATCTGGGAGTCCACTAGCATCTGGTACTCCTAGAAC

TACCGCACCTGCACCCTCATCTGCCTCAAGCCTTCGCTTTAACTATTTAG

ATTTGATTATTGGCACTCTTATTCCTTTATTCATTGGTGGTTTATTATAT

TAA

gttcggataaatttctttag

>Sccal_AGL6_bracas_a_k49_L1_T2359of38456_k59_L16879_T1_432nt

gaaatacaataatatacgat

ATGA

ATCTCACTAAATCTACAATCATTTTCCTCGTCCTATGCATTCTTCTCGTC

ACTGACGTTCTTGCACAACAACCATCTGCTGCGGGGCCTAGACCTAGCGC

TG

GTGCTGCTCCAAACGGTCCTGGTCCAAGTGGTGCTGCTCCAAACGGTCCT

GGTCCAAGTGGTGCTGCTCCAAACGGTCCTGGTCCAAGCGGTGCTGCTGC

TCCAGCAGGACCTGCTCCAAGTGGTGCTGCTGCTCCAGCAGGACCTGCTC

CAAGTGGTGCTGCACCCAAACAAGGATCACCACCACCATCATCTACAGGA

GGATCCTCCGCTGCTTCTACTTCATCCGCTGCCAAGACTGGTGCTGCATC

AAACATTCGAATTGAATGTTTTGAATTGGTCATTGGTTCTCTCGCTGCTT

TATTCGTTGGTGGTTTATTATATTAA

gttcgaatataattgtttat

>Sccal_AGL7_bracas_b_k59_L4425_T1of2_eck69_425nt

ttttaaaaattaaatacgaa

ATGAATATCACTAAAAGTACGATTATCTTCCTTGTCCTGTGCAT

TCTTCTCCTTTCTGGTGTTCTTGCTCAAACTGATACAGCTAGTGCTGCTG

CTCCTAATGGTACCCCACCACCGGGACCGGGAGGACCTCCAGGACAAGGA

CCACCTAGTGGTGCAGCAGCACCGAGACCAAGTGGTGCAGCAGCACCGGG

AGCAAGTGGTGCAGCACCAGCAGCAGCACCTGGAGCTAGTGGTGCACCAG

CAGCAGCACCTGGAGCTAGTGGTGCACCAGCAGCACCTGGTGCTCCCAA

ATCACCGTCGCCCACAAGTTCTGGAGGAGCAGCACCAGCTAGCTCAACTA

GTTCTAACAGAAATACTGCTTCAAGCATTCGAGTTGAAAATTTTGAATTG

GTCATCGGTTCTCTTATTGCTTTATTCATTGG

>Sccal_AGL8_bracas_i_k59_L4622_T1_trunc_558nt

tattaagcatatatcacata

ATGGCATCACTACGACGCATCCAAATTTTTCTTTTA

TTTATTATTGTATCATTATTCATTGCAAATACTGGAAATGTAAATGCTCAAAATAATACA

GGAGGAGCAGCACCAGCACCAGCAGTGTCGCAACCACCAGTAGCAGCAGATACCCCGCCA

GCAGGGCAAGCTCAAACTAATAATATATTACCTACTGTTATACCAGAACCTTCGACACCA

GCACAAATTACAGAGCCGCCGCCGCCGCCAGTTTCAAATCCCACACCAGCACCAGCACCA

GCACCAGCGCCTACACAAGCACCACCAAAACAACAATCCCAAGCCCCTCAACAAGGTGGT

AAAATTACCGCGAAAGCTCCCGTTTTACCTTCTGGTCCTAGTGCCATCACTATTACATCC

ACGTCATATGTAGATTCTGCAACTCCTCCTCCAAAGAATAATAGTAATTCCGCCAATAGC

AATAGTAGTGATTCCAGTAGTTCTACTAGTAGTATTATTACAGCTGCTATTGTTGTTGGT

ACCGTGGTTGTCGCTGCGGCTATTGGTATATGGATATTTAGA

>Sccal_AGL1_bgiros2b_k49_L6948rc_T3_3_207aa

MNMTKNTIIFFVRCIFFISSILA

QAPGPAFSPAPAPASALNPAPAP

ASALNPAPAPAPVPNPNFSRVPFPAPSPAPTPTPAPFPAKAPTLPPASFLAPVSAFSLAS

DLAPISTPDLSLASDLASASAPALISDTSAPTSEPAPVITQPPPPSGSNKNLASSGSAPI

PNSTGPVSTKFIPPSISGVHINYLELIIGPLVALLIGVLLY

>Sccal_AGL2_bracas_f_k49_L3437_T2of2_171aa

MSRLVFLTLLLVFIGFTSA

QVSSVNTPGPKIAVGATVPITWTYTPQVNALPGTLSV

IDSVTQSSTIISSSIVLSNKNYQWVVNVPAGSYYLGLNDGSGNKLSGVFQVFDPNSSSPA

ANPGSPAAPAPSGGSASNSSSPSTTSQKSIA

ASFTVPINLLFNLIVVAAVMVYFV

>Sccal_AGL3_bracas_g_k49_L1_T5777of30356_167aa

MSRLIFLTIFLVFIGFTSA

QVTSVKAAPSSVIVGSPVTCTWNYTGAAATGNLSLVDETTKA

ATVISSAINLTSLSYSYQTTTPGDFWFSLSDGTSNSLSNVFHVLTPAQASASSTGAPSPS

DTSSPNGGSPGSSAKKS

AASAKSSFTVSNKLLIGLIMIVTVMMFFA

>Sccal_AGL4_bracas_h_k59_L1738_T2_part_94aa

LALNDGSGNKDTGTFTVFNPAGAAPAPAPAASAAPAASGTAPAATSAAPAAPRPPPSGAP

AQSAPAPAQAPAASQGPAQPQASAKSTSPSSAAP-VFKFQHKP

>Sccal_AGL5_bracas_a_k59_L5940_T1_180aa

MNIARNIIIFLIVCILISGVLA

QGGSPSNS

PGGPQGPPTGGPQGPRPGGPQSGGPQDQGPPTGGPQDQGPPTGGPPTGGSPTGGPQDQGP

PTGAPTGAPQGPSGTAGPAGPQGTPGTAGSPQTGPTPTGSHAGSSSGSPLASGTPRTTAP

APS

SASSLRFNYLDLIIGTLIPLFIGGLLY

>Sccal_AGL6_bracas_a_k49_L1_T2359of38456_k59_L16879_T1_141aa

MNLTKSTIIFLVLCILLVTDVLA

QQPSAAGPRPS

AAPNGPGPSGAAPNGPGPSGAAPNGPGPSGAAAPAGPAPSGAAAPAGPAPSGAAPKQGSP

PPSSTGGSSAASTSSAAKTG

AASNIRIECFELVIGSLAALFVGGLLY

# Sccal_AGL7 partial sequence (no stop codon) but likely missing <9 nt or 1-2 aa

>Sccal_AGL7_bracas_b_k59_L4425_T1of2_eck69_141aa

MNITKSTIIFLVLCILLLSGVLA

QTDTASAAAPNGTPPPGPGGPPGQGPPSGAAA

PRPSGAAAPGASGAAPAAAPGASGAPAAAPGASGAPAAPGAPKSPSPTSSGGAAPASSTS

SNRNTA

SSIRVENFELVIGSLIALFI

>Sccal_AGL8_bracas_i_k59_L4622_T1_trunc_186aa

MASLRRIQIFLLFIIVSLFIANTGNVNA

QNNT

GGAAPAPAVSQPPVAADTPPAGQAQTNNILPTVIPEPSTPAQITEPPPPPVSNPTPAPAP

APAPTQAPPKQQSQAPQQGGKITAKAPVLPSGPSAITITSTSYVDSATPPPKNNSNSANS

NSSDSSSSTSSIITAAIVVGTVVVAAAIGIWIFR

(l) Sccal_mira (DNA, then protein)

# Sccal_AGL1_mira CDS same as OASES

>Sccal_AGL1_mira_a1_c3_444reads

# Sccal_AGL2_mira CDS same as OASES

>Sccal_AGL2_mira_a1_c5_216reads

# Sccal_AGL3_mira 3 ambiguities in MIRA sequence – use OASES

>Sccal_AGL3_mira_a1_c9_68reads

# Sccal_AGL4_mira is longer than oases sequence, but still does not have

# an ER or GPI signal. It is 88 nt longer at the 5’ end (all coding sequence),

# and has an additional 90 nt at the 3’ end from where the two sequences diverge

# repeated MIRA to get longer sequence

>Sccal_AGL4_mira_a1_c6rc_110reads_461nt

ACTCAAAACACAACCATAATTAGCAATACAATAAACCTTGCTTCACAATCTTACCAATGG

ACGGTTAATGTTCCTGCGGGTACTTACTACCTTGCTCTCAATGATGGATCTGGTAATAAG

GACACTGGAACTTTCACAGTTTTTAATCCTGCTGGTGCTGCACCCGCACCTGCACCTGCT

GCTTCAGCTGCTCCAGCTGCATCTGGCACCGCCCCGGCTGCAACTAGCGCCGCTCCGGCT

GCACCTCGCCCACCACCATCTGGTGCTCCAGCTCAATCAGCTCCAGCTCCAGCTCAAGCT

CCCGCCGCTTCTCAAGGTCCTGCTCAACCTCAAGCATCTGCTAAATCTACATCCCCTAGT

TCAGCTGCACCAAAATCTAGTTCAGCACCAACAACACCAACCAATGCTGCTGCAAGCTTC

ACTGGATCAAGCATTTCTGGATACAAACTTCTATTTAGCTT

# repeated MIRA with the above seq

# used Sccal_AGL4_mira**R2** sequence to search TSA (NCBI) and got full length sequence

# see sequence following miraR2

>Sccal_AGL4_mira**R2**_a1_c6rc_110reads_572nt

TTGCAGTAGGCGCTACTGTTACAATCACTTGGGTATATACACCACAAACT

AATGCAATCCCAGGAATTTTAAGCTGTATCGATAGCACAACTCAAAACAC

AACCATAATTAGCAATACAATAAACCTTGCTTCACAATCTTACCAATGGA

CGGTTAATGTTCCTGCGGGTACTTACTACCTTGCTCTCAATGATGGATCT

GGTAATAAGGACACTGGAACTTTCACAGTTTTTAATCCTGCTGGTGCTGC

ACCCGCACCTGCACCTGCTGCTTCAGCTGCTCCAGCTGCATCTGGCACCG

CCCCGGCTGCAACTAGCGCCGCTCCGGCTGCACCTCGCCCACCACCATCT

GGTGCTCCAGCTCAATCAGCTCCAGCTCCAGCTCAAGCTCCCGCCGCTTC

TCAAGGTCCTGCTCAACCTCAAGCATCTGCTAAATCTACATCCCCTAGTT

CAGCTGCACCTTCAGCACCAACAACACCAACCAATGCTGCTGCAAGCTTC

ACTGGATCAAGCATTTCTGGATACAAACTTCTATTTAGCTTAATTGTAGT

AGCTGCAGTTATGGTACATTTT

# Sccal_AGL4_TSA Full length Sccal_AGL4 from TSA dataset

>Sccal_AGL4_TSA_GGIE01031823.1_127..819

ATGTCACGATTTAT

TTATCACCTTACGTTATTATTATCGGTATTTTTGATCTTTTTTGAATCTACCTCAGCCCAGGTTACAACA

CTCAATACACCTTCTGGTAGTATTGCAGTAGGCGCTACTGTTACAATCACTTGGGTATATACACCACAAA

CTAATGCAATCCCAGGAATTTTAAGCTGTATCGATAGCACAACTCAAAACACAACCATAATTAGCAATAC

AATAAACCTTGCTTCACAATCTTACCAATGGACGGTTAATGTTCCTGCGGGTACTTACTACCTTGCTCTC

AATGATGGATCTGGTAATAAGGACACTGGAACTTTCACAGTTTTTAATCCTGCTGGTGCTGCACCCGCAC

CTGCACCTGCTGCTTCAGCTGCTCCAGCTGCATCTGGCACCGCCCCGGCTGCAACTAGCGCCGCTCCGGC

TGCACCTCGCCCACCACCATCTGGTGCTCCAGCTCAATCAGCTCCAGCTCCAGCTCAAGCTCCCGCCGCT

TCTCAAGGTCCTGCTCAACCTCAAGCATCTGCTAAATCTACATCCCCTAGTTCAGCTGCACCAAAATCTA

GTTCAGCACCAACAACACCAACCAATGCTGCTGCAAGCTTCACTGGATCAAGCATTTCTGGATACAAACT

TCTATTTAGCTTAATTGTAGTAGCTGCAGTTATGGTACATTTTGCCTAA

# Sccal_AGL5_mira CDS same as OASES

>Sccal_AGL5_mira_a1_c1_604reads

# Sccal_AGL6_mira has a deletion of 99 nt compared to the oases sequence in the

# middle GC rich region. No change to ER/GPI signals

# use Oases sequence – as Mira seems too short

>Sccal_AGL6_mira_a2_c6_137reads_333nt

ATGAATCTCACTAAATCTACAATCATTTTCCTCGTCCTATGCA

TTCTTCTCGTCACTGACGTTCTTGCACAACAACCATCTGCTGCGGGGCCTAGACCTAGCG

CTGCTCCAAACGGTCCTGGTCCAAGTGGTGCTGCTGCTCCAGCAGGACCTGCTCCAAGTG

GTGCTGCACCCAAACAAGGATCACCACCACCATCATCTACAGGAGGATCCTCCGCTGCTT

CTACTTCATCCGCTGCCAAGACTGGTGCTGCATCAAACATTCGAATTGAATGTTTTGAAT

TGGTCATTGGTTCTCTCGCTGCTTTATTCGTTGGTGGTTTATTATATTAA

# Sccal_AGL7_mira CDS same as OASES

>Sccal_AGL7_mira_a2_c1_505reads

# Sccal_AGL8_mira is 76 nt longer (25aa), but is still

# not predicted to be GPI and no stop codon

# Sequence here includes 20 nt of 5’UTR as used to repeat MIRA (miraR2)

>Sccal_AGL8_mira_a2_c2rc_505reads_634nt

tattaagcatatatcacata

ATGGCATCACTACGACGCATCCAAATTTTT

CTTTTATTTATTATTGTATCATTATTCATTGCAAATACTGGAAATGTAAATGCTCAAAAT

AATACAGGAGGAGCAGCACCAGCACCAGCAGTGTCGCAACCACCAGTAGCAGCAGATACC

CCGCCAGCAGGGCAAGCTCAAACTAATAATATATTACCTACTGTTATACCAGAACCTTCG

ACACCAGCACAAATTACAGAGCCGCCGCCGCCGCCAGTTTCAAATCCCACACCAGCACCA

GCACCAGCACCAGCGCCTACACAAGCACCACCAAAACAACAATCCCAAGCCCCTCAACAA

GGTGGTAAAATTACCGCGAAAGCTCCCGTTTTACCTTCTGGTCCTAGTGCCATCACTATT

ACATCCACGTCATATGTAGATTCTGCAACTCCTCCTCCAAAGAATAATAGTAATTCCGCC

AATAGCAATAGTAGTGATTCCAGTAGTTCTACTAGTAGTATTATTACAGCTGCTATTGTT

GTTGGTACCGTGGTTGTCGCTGCGGCTATTGGTATATGGATATTTAGAAAATGGAAACTC

ACGCCATCACGAAATTTCAAAGAAAAGATTCAACCTGTCGATTTTGGTCCACGTTCAGTA

GAAT

# Repeated MIRA with seq above and found 30 nt longer,

# but protein encoded by Sccal_AGL8_miraR2 is still

# not predicted to be GPI and no stop codon

>Sccal_AGL8_mira**R2**_a1_c1_595reads_664nt

ATGGCATCACTACGA

CGCATCCAAATTTTTCTTTTATTTATTATTGTATCATTATTCATTGCAAATACTGGAAAT

GTAAATGCTCAAAATAATACAGGAGGAGCAGCACCAGCACCAGCAGTGTCGCAACCACCA

GTAGCAGCAGATACCCCGCCAGCAGGGCAAGCTCAAACTAATAATATATTACCTACTGTT

ATACCAGAACCTTCGACACCAGCACAAATTACAGAGCCGCCGCCGCCGCCAGTTTCAAAT

CCCACACCAGCACCAGCACCAGCACCAGCGCCTACACAAGCACCACCAAAACAACAATCC

CAAGCCCCTCAACAAGGTGGTAAAATTACCGCGAAAGCTCCCGTTTTACCTTCTGGTCCT

AGTGCCATCACTATTACATCCACGTCATATGTAGATTCTGCAACTCCTCCTCCAAAGAAT

AATAGTAATTCCGCCAATAGCAATAGTAGTGATTCCAGTAGTTCTACTAGTAGTATTATT

ACAGCTGCTATTGTTGTTGGTACCGTGGTTGTCGCTGCGGCTATTGGTATATGGATATTT

AGAAAATGGAAACTCACGCCATCACGAAATTTCAAAGAAAAGATTCAACCTGTCGATTTT

GGTCCACGTTCAGTAGAATCAGATGAACTATTTTTACGCGGATTACAAG

>Sccal_AGL4_mira_a1_c6rc_110reads_153aa

TQNTTIISNTINLASQSYQWTVNVPAGTYYLALNDGSGNKDTGTFTVFNPAGAAPAPAPAASAAPAASGTAPAATSAAPAAPRPPPSGAPAQSAPAPAQAPAASQGPAQPQASAKSTSPSSAAPKSSSAPTTPTNAAASFTGSSISGYKLLFS

# For Sccal_AGL4 did a second round of mirabait, then MIRA=R2, and got

# a GPI-anchored protein

# It is a putative orthologue of Racas_AGL6 (which as ER and GPI-signal sequences)

# Based on needle pairwise alignment, **Sccal_AGL4 is missing approx**

# 32 aa at the N-terminus (the presumed ER signal sequence and 12 aa of the

# mature protein) and a single aa (possibly A) at the C-terminus (later confirmed

# based on a sequence at the transcript shotgun assembly (TSA) database.

# It is assumed that the full length sequence when obtained will include

# an ER-signal sequence and therefore this partial sequence is not eliminated.

# A search of TSA dataset at NCBI obtained a full length Sccal_AGL4

# see next sequence

>Sccal_AGL4_miraR2_a2_c2_190aa

AVGATVTITWVYTPQTNAIPGILSCIDSTTQNTTIISNTINLASQSYQWTVNVPAGTYYLALNDGSGNKDTGTFTVFNPAGAAPAPAPAASAAPAASGTAPAATSAAPAAPRPPPSGAPAQSAPAPAQAPAASQGPAQPQASAKSTSPSSAAPSAPTTPTNA

AASFTGSSISGYKLLFSLIVVAAVMVHF

# Full length Sccal_AGL4 from TSA dataset

>Sccal_AGL4_TSA_GGIE01031823.1_127..819

MSRFIYHLTLLLSVFLIFFESTSA

QVTTLNTPSGSIAVGATVTITWVYTPQTNAIPGILSCIDSTTQNTTIISNTINLASQSYQWTVNVPAGTYYLALNDGSGNKDTGTFTVFNPAGAAPAPAPAASAAPAASGTAPAATSAAPAAPRPPPSGAPAQSAPAPAQAPAASQGPAQPQASAKSTSPSSAAPKSSSAPTTPTNA

AASFTGSSISGYKLLFSLIVVAAVMVHFA

# Sccal_AGL6_mira sequence is shorter by 31 amino acids in the mature protein

# ER and GPI signal sequences are the same

>Sccal_AGL6_mira_a2_c6_137reads_110aa

MNLTKSTIIFLVLCILLVTDVLA

QQPSAAGPRPSAAPNGPGPSGAAAPAGPAPSGAAPKQGSPPPSSTGGSSAASTSSAAKTG

AASNIRIECFELVIGSLAALFVGGLLY

>Sccal_AGL8_mira_a2_c2rc_505reads_211aa

MASLRRIQIFLLFIIVSLFIANTGNVNA

QNNTGGAAPAPAVSQPPVAADTPPAGQAQTNNILPTVIPEPSTPAQITEPPPPPVSNPTPAPAPAPAPTQAPPKQQSQAPQQGGKITAKAPVLPSGPSAITITSTSYVDSATPPPKNNSNSANSNSSDSSSSTSSIITAAIVVGTVVVAAAIGIWIFRKWKLTPSRNFKEKIQPVDFGPRSVE

>Sccal_AGL8_miraR2_a1_c1_595reads_221aa

MASLRRIQIFLLFIIVSLFIANTGNVNA

QNNTGGAAPAPAVSQPPVAADTPPAGQAQTNNILPTVIPEPSTPAQITEPPPPPVSNPTPAPAPAPAPTQAPPKQQSQAPQQGGKITAKAPVLPSGPSAITITSTSYVDSATPPPKNNSNSANSNSSDSSSSTSSIITAAIVVGTVVVAAAIGIWIFRKWKLTPSRNFKEKIQPVDFGPRSVESDELFLRGLQ

(m) Racas_oases (DNA, then protein)

>Racas_AGL1_bGiros2a_qk39_L43205rc_pm20_286nt

CTCTGAAGCACCTATTGAACCAGCCGGACCGGCGCCATCTGGAACAACACCTACTGGACC

CGCCGAACCGCCATCACCTGGACCATCACAATCTAGACTACCAGCTTCAAACTCCAGCGG

TCCGGCAACTTCTGGTGCACAAGGTCCCGTTAGTACTCCTAGTGTCAAATCTTCAACAAC

TGCACCTTCAGTCATCACTGCTGCTGCTTCAGAGGTTCGCGTTAACTGTTTAGAATTGAT

CACAGGATCTTTTATTACTTTGTTCATTTTTGGTTTATTATATTAA

atttttgatagagtactttg

>Racas_AGL2_bk39L38885rc_qk59_L10733_pm20_630nt

ataaaaaataataatacaaa

ATGAATATCACTAAGAA

TACATTCTTCCTCCTCCTCGTATGCATTCTTTTCGTCTCTGTATTTGCTCAACAACAACC

AAAACAACCACCACCGTCTGGAGGCCCTGACAACGCACCAGAACCATCGGGCAATCCTAA

AGGTCCAGGAGGTCCAGGAGAACAAAGTGCCCCAGCAGGAGCACCAGGCCCAGCAGCTAC

CGGACCAGCACAACCCGGTTCAAGCGGAACACCACCAGGACCAAACGCCCCCGGATCAAG

TGGAGCCCCCGGAGCCCCCGCCCCTGGACCATCCAATGCACCAGCAGGTGCACCAGGTGC

TAGTGGAGCCCCAGCCTCTCCAACTGGTGCTTCTAGTCCTAAGGGTAGTGAACCTGCTGC

CGCTCCAAAGAGCTCAGGAGCTTCTCCTGCCGGTGCACCGTAAGTATTTTTAAGAAGTCT

TTGGTTTAATCATTTTTAAATAATCAATGATATTAATTTATTATAATAAATGCCTGCCGG

TGCACCAAAATCAAGCAATGCTGCATCCACTACCGCTCCCGCCTCAGGTGCCGTTTCAAG

CGTTCGTTTCAGCGGTTTTGAATTGATCGTTGGATCTTTCGCTGCTTTATTCATCGGTAC

CTTATTATACTAA

gcttttgatataattttatt

>Racas_AGL3_bGiros2b_qk39_L39398_pm20_429nt

tgtttatattttaatacgaa

ATGAATAT

CACTAAGAGTATAATTATCTTTTTCGTTGTGTGTATGTTTATTGCTTCCA

GCGTTCTTGCCCAAGACACTGTTTCTAATGCAGTAGAACCTTCAACTACA

CCTGGAGGAGAACCATCTCCAGCTCCACCCGGTGCTTCAAGTGACACTCC

TTTAGGTAGCTCCACACCGACAGGATCTGTTACTTCTGCACCGACAGTTG

CCGCTGTTTCTGCGCCGACAGGTTCTGCTACTGGTTTAGCTGCCGCTTCT

CCCGCTTCTCCCGCTTCAGCTACTGAAAGTGGCAAAAGTACTACCACGCC

TTCCACTACAAAACCCCCAACTACAACAAGTGCGAGTGCTGCTTCAAATG

TTCAAGTTGGCAGTATTGAATTAATCAGTGGTTATTTGATCGCTTTATTC

ATTGGTGGTTTATTATTTTAA

ttttggtccgcagctcaaag

>Racas_AGL4_bk39_L2_qk49_L10394_pm20_393nt

tccatatttaataatacgaa

ATGAAT

TTCACTAAAAGTACAATTATTTTATTCTTATGCATTCTTATCGCTTCTGG

CGTTCTTGCGCAAGAAACCGGCGCAAAAGGACCCGCCCCCACAGATGCAA

AAGCTCCGCCGAGTCCTGCAGCTCCTTCTCCTAGTGGTGCAGCTCCTGCC

AGTGGTGCAGCTCCTGCTCCCACAGATGCAAAAGCTCCGGCTCCGAGTGG

TGCAGGTCCTGCTGCCAGTGGTCCTCCTGCTGGTAAAGGCGGAGAACCTA

AAGCTTCAGGAGAAGCTCCCGCATCCACATCCCCTCCCAAGTCGGCTGCT

GCTTCAAACGTTCGCGATAACCATTTTGAATTGATCGTTGGTTCTTTAGT

TGCTTTATTCATTGGTGGTTTAGGTTTATTATATTAA

gcatgaatgtaactttgtta

>Racas_AGL5_bL12863_qk49_L13686_pm20_435nt

atagcatattttaatacaaa

ATGAAAAT

CCCTAAGAGTACAATCATCTTTATTGTATGTATCTTCATCGCCTCCGGCG

TTCTTGCACAGCAACAAACTCCACCAACGGGACAAGCTCCACCATCAGGT

GCATCTCAACCTAAAGGACCAGCTCAGACCGATGCTGCTAAAACTCCTGC

TGCATCGGGTGCTCCCGCTCCTTCTGGTGCTCCAGCTGCTCCTTCTGGTG

CTGCTCCAGCTGCTCCTTCTGGCGCCGCTCCAGCTCCTTCTGGTGGTCCA

GGTCCAGGTCCAGCTCCAAAAGGCACTGATGCTGGCAATGGCAAGGCTCC

GCCTTCTACTCCCACTTCCTCTGCCAAACCTTCAAGTGCTGCTTCTTCTT

TAACTGCTCAACTTAGCAATGTTGAATTATTCAGTGGTTATTTGATTGCT

TTATTCATTGGTGGTTTATTATTTTAA

gctcaattttgactatccgc

>Racas_AGL6_bGiros2e_qk39_L8147_pm20_639nt

taacccggacataagtaact

ATGTCACGATT

CATTTATTTCCTCACTCTATTCCTGGCCTTTATTGGGTCTACTTTAGCCC

AGGTTCAATCAATTAACACGCCTGGCGGTAGTGTTGCAAATGGTGCCACT

ATTACAATCACCTGGATATACACAACACAACCGAACCCACTTCCAGGAAC

TCTAAGTGTTGTCGACAACACGACTAAGAACACTGTTATAATTAGCAGTA

ATGTAACTCTCTCTACTCAATCTTACCGATGGACTGTCAATGTTCCAGCT

GGAACTTATTACCTTGCTCTCAATGACGGGTCTGGTGATAAATATTCTGG

AACTTTCACTGTCTTCCAAGCCGGTGCACCCCCTGCACCCGCTGCTTCTG

GCACCGCAGCCGCTGCACCTCCAGCAGCATCTGGTGCACCTGCTGCCAGT

TCCCCTGCTGCTGCACCTGCCGCAACTTCTGCAGCTGCTCAACCTCAACC

TCCACCTCAACCATCCCCTAAACCACCTGCGAACACTGAAAGTTCAGCTG

CGAAATCATCATCGACACCTACGCCACCAGCAGCCAGTGCTGCAAGTTTT

ACTGGATCAAGCATTTCTGGATACAAACTTCTGTTTAGCCTTATTGTAGT

AGCAGCAGTAATGGTTCATTTTGCTTGA

aatttataatcctagaagaa

>Racas_AGL7_bGiros2e_2_qk39_L98_pm20_660nt

ctaacctgacataagtaacc

ATGTCACGATTCATTTATTTTCTCACTCTATTCTTGGTTTTTAT

TGGGTCTACTTTAGCCGGTGTTGACGTTGTTAACACGCCTACTGGTAATA

TTGCTCGAGGTGCCTCTATTCCGATCACTTGGACCTATACTCCAGGGAAT

GCGACTGCGGGTGTTTTACGAGTTGTCGATAACTCAACTCAGAACTCTAA

AACAATTGATGATAAGCTAAACCTCGCTACCCAATCCTACCAATGGACGG

TCGATGTTGTCCCGGGTGTTTATTATTTTGCTCTCAACGACGAGACCGGT

GATAAATATTCTGGACAGTTCACCGTTGTTCAAGGTAGCGCCCCCCCCTC

TGGCTCTGCAGCTCCAGCTGCTCCAAGTCAAACTCAATCTCGCGCTACAT

CTGTTCCTGCTGCCCCCGCTGCCACTAACACTCCCCCTGCTTCATCTTCT

CAACCTCAACCTCAACCTCAACCTCAGCCTCAATCTGCTAAACAACCCGC

TGCCGCTCAAAGTACACCTGCGGGAAATACGACACCCTCTGCTGGTAAAT

CAACATCGGCACCTTCAGCCAATACTGCCGCAAGTTTCACTGGATCAAGC

TTTTCTGGATACAAACTTCTGTTTAGTCTTATTGTAGTAGCCGCAGCAAT

GGTACATTTTGCCTAA

aaatttataatcctagaact

>Racas_AGL8_bGiros2e_3_qk39_L4637_pm20_573nt

caacctgaaaagaaataatc

ATGTCTCGATTTATTTT

TCTACTCTTAATTTTTATTGGGTTTACTTCAGCCCAGGTTCAAACAATCAACACACCTAG

TGGTAGAGTTGCTAATGGTGCCAACATTCTAATCACTTGGACGTATACCGCACAAGCGAA

TCCGCTTCCAGGCACTTTAAGTGTCATCGACAACACAACTAAAAATATCACTATTATTAG

CAATACAATAGACCTCTCTGCTCAAGCTTACCAATGGATAGTCAATGTTCCCGCGGGCAC

GTATTACCTTGCTCTCAACGATGGTTCTGGTGATAAGTACTCGGGAAATTTCGAAGTCTT

TAATGCCGGTCAACCGGCTCAAGCGAGTGCCCCAGCTCAAATGAGTTCTCCAGCTCAAAA

TAATGCATCCCCTCCCCCTCCACCCGCTCCTGCTCCTACTCCCAAACCTGCTAATTCACC

GACTAGTTCTAGCGCTAGCACCAAAGCGAATACACCACCAGCCCAGATAAATGCTGCTTC

AAGTTTCATTGGGTCATCCTGCAAACTTTTGCTTAATTTTATTACGGTAGCAATAGTCAT

GGTATACTTTGTCTAA

tataaatatacgtgacaatt

>Racas_AGL9_qk39_L3025_AGLpartonly_m20p100_546nt

ttactagcatatatcatata

ATGG

CATTACTACGACACAACCAGATTTCCATTTTATTTATTATTGTATTATTA

TTCATCGCAAATGTTGCAAATGTAAATGCTCAAGCATCAGGAGCGGCATC

AGGAGGACAACCAGCAGCAACACTACTACCACCACCACCACCACCACCAG

CAGTTACACAGCCTCCGCCGCCTCCCGCTAGCGAGCCTGCTAGTAATGTA

GGACCACCAGCACAACCTACAGATTCGCCACAACCGCAACCACAATCAGA

ACCTCCAAGTCAACCCACAGCACCACCACCACAACAACCTTCGTCTCCCA

AGGCGGCACCAAGTTCGCCAGCTCAACAACAAAAATCAGCCAAACAAGTT

ATCACCGCAACTAATAATGGGCCTGTTGCTATCACTATTACATCTACATC

ATTCGTAGATACCCCAACTGGTAAACCAAATGTTAATAGTGCCAATAGCA

ACGATAATTCTGGTGCTACCGGCAGTATTATCACGGCTGCTATCGTTGTT

GGTACTGTGGTTGTTGCTGCTGCTATCGGCATATGGATATTT

AGAAAATG

GAAACTTACGCCATCACGAAACTTCAAAGAAAAGATTCAACCTGTTGATT

TTGGGCCACGTTCAGCAGAATCAGATGAAATATTTTTACGTG

>Racas_AGL1_bGiros2a_qk39_L43205rc_pm20_94aa

SEAPIEPAGPAPSGTTPTGPAEPPSPGPSQSRLPASNSSGPATSGAQGPVSTPSVKSSTT

APSVITAA

ASEVRVNCLELITGSFITLFIFGLLY

# AGL2 would be GPI-anchored if not for the frameshift

>Racas_AGL2_bk39L38885rc_qk59_L10733_pm20_208aa

MNITKNTFFLLLVCILFVSVFA

QQQPKQPPPSGGPDNAPEPSGNPKGPGGPGEQSAPAGAPGPAAT

GPAQPGSSGTPPGPNAPGSSGAPGAPAPGPSNAPAGAPGASGAPASPTGASSPKGSEPAA

APKSSGASPAGAP-VFLRSLWFNHF-IINDINLL—MPAGAPKSSNAASTTAPAS

GAVSSVRFSGFELIVGSFAALFIGTLLY

>Racas_AGL3_bGiros2b_qk39_L39398_pm20_142aa

MNITKSIIIFFVVCMFIASSVLA

QDTVSNA

VEPSTTPGGEPSPAPPGASSDTPLGSSTPTGSVTSAPTVAAVSAPTGSATGLAAASPASP

ASATESGKSTTTPSTTKPPTTTSASAA

SNVQVGSIELISGYLIALFIGGLLF

>Racas_AGL4_bk39_L2_qk49_L10394_pm20_130aa

MNFTKSTIILFLCILIASGVLA

QETGAKGPAPTDAKAPPSPAAPSPSGAAPASGAAPAPTDAKAPAPSGAGP

AASGPPAGKGGEPKASGEAPASTSPPKSA

AASNVRDNHFELIVGSLVALFIGGLGLLY

>Racas_AGL5_bL12863_qk49_L13686_pm20_144aa

MKIPKSTIIFIVCIFIASGVLA

QQQTPPTGQAPPSGASQPKGP

AQTDAAKTPAASGAPAPSGAPAAPSGAAPAAPSGAAPAPSGGPGPGPAPKGTDAGNGKAP

PSTPTSSAKPSS

AASSLTAQLSNVELFSGYLIALFIGGLLF

>Racas_AGL6_bGiros2e_qk39_L8147_pm20_212aa

MSRFIYFLTLFLAFIGSTLA

QVQSINTPGGSVANGATITITWIYTTQPNPLPGTLSV

VDNTTKNTVIISSNVTLSTQSYRWTVNVPAGTYYLALNDGSGDKYSGTFTVFQAGAPPAP

AASGTAAAAPPAASGAPAASSPAAAPAATSAAAQPQPPPQPSPKPPANTESSAAKSSSTP

TPPAAS

AASFTGSSISGYKLLFSLIVVAAVMVHFA

>Racas_AGL7_bGiros2e_2_qk39_L98_pm20_261aa

MSRFIYFLTLFLVFIGSTLA

GVDVV

NTPTGNIARGASIPITWTYTPGNATAGVLRVVDNSTQNSKTIDDKLNLATQSYQWTVDVV

PGVYYFALNDETGDKYSGQFTVVQGSAPPSGSAAPAAPSQTQSRATSVPAAPAATNTPPA

SSSQPQPQPQPQPQSAKQPAAAQSTPAGNTTPSAGKSTSAPSANTA

ASFTGSSFSGYKLLFSLIVVAAAMVHFA

>Racas_AGL8_bGiros2e_3_qk39_L4637_pm20_190aa

MSRFIFLLLIFIGFTSA

QVQTINTPSGRVANGANILITWTYTAQAN

PLPGTLSVIDNTTKNITIISNTIDLSAQAYQWIVNVPAGTYYLALNDGSGDKYSGNFEVF

NAGQPAQASAPAQMSSPAQNNASPPPPPAPAPTPKPANSPTSSSASTKANTPPAQINAA

SSFIGSSCKLLLNFITVAIVMVYFV

>Racas_AGL9_qk39_L3025_AGLpartonly_m20p100_182aa

MALLRHNQISILFIIVLLFIANVANVNA

QASGAASGGQPAATLLPPPPPPPAVTQPPPPP

ASEPASNVGPPAQPTDSPQPQPQSEPPSQPTAPPPQQPSSPKAAPSSPAQQQKSAKQVIT

ATNNGPVAITITSTSFVDTPTGKPNVNSANSNDNSGATGSIITAAIVVGTVVVAAAIGIW

IF

(n) Racas_mira (DNA, then protein)

# Racas_AGL1_mira is 36 nt shorter at N-terminus than the OASES sequence,

# Use the OASES sequence

>Racas_AGL1_mira_a2_c9_16reads

# Racas_AGL2_mira resolved an insertion/frameshift in the oases sequence

# that resulted in a GPI anchored protein

>Racas_AGL2_mira_a2_c7_228reads_543nt

ATGAATATCACTAAGAATACATTCTTCCTCCTCCTCGTATGCATTCTTTTCG

TCTCTGTATTTGCTCAACAACAACCAAAACAACCACCACCGTCTGGAGGCCCTGACAACG

CACCAGAACCATCGGGCAATCCTAAAGGTCCAGGAGGTCCAGGAGAACAAAGTGCCCCAG

CAGGAGCACCAGGCCCAGCAGCTACCGGACCAGCACAACCCGGTTCAAGCGGAACACCAC

CAGGACCAAACGCCCCCGGATCAAGTGGAGCCCCCGGAGCCCCCGCCCCTGGACCATCCA

ATGCACCAGCAGGTGCACCAGGTGCTAGTGGAGCCCCAGCCTCTCCAACTGGTGCTTCTA

GTCCTAAGGGTAGTGAACCTGCTGCCGCTCCAAAGAGCTCAGGAGCTTCTCCTGCCGGTG

CACCAAAATCAAGCAATGCTGCATCCACTACCGCTCCCGCCTCAGGTGCCGTTTCAAGCG

TTCGTTTCAGCGGTTTTGAATTGATCGTTGGATCTTTCGCTGCTTTATTCATCGGTACCT

TATTATACTAA

# Racas_AGL3_mira CDS same as OASES

>Racas_AGL3_mira_a1_c9_36reads

# Racas_AGL4_mira CDS same as OASES

>Racas_AGL4_mira_a1_c3_356reads

# Racas_AGL5_mira, not used – two contigs – use OASES sequence

>Racas_AGL5partial_mira_a2_c4_291reads_95..475_100%

>Racas_AGL5partial_mira_a2_c10_28reads_1..193_100%

# Racas_AGL6_mira CDS same as OASES

>Racas_AGL6_mira_a2_c5_216reads

# Racas_AGL7_mira CDS same as OASES

>Racas_AGL7_mira_a2_c6_251reads

# Racas_AGL8_mira CDS same as OASES

>Racas_AGL8_mira_a1_c1_703reads

# Racas_AGL9_mira CDS same as OASES

# A search of TSA dataset at NCBI obtained a longer Racas_AGL9

# with a stop codon, but doesn’t look real (see below)

# is <40%PGA (see Table S8)

# protein composition analyses (Table S6) for Racas_AGL9 was done on Oases Seq

>Racas_AGL9_mira_a1_c2_520reads

>Racas_AGL9_TSA_GGID01054367.1_525..1187

MALLRHNQISILFIIVLLFIANVANVNA

QASGAASGGQPAATLLPPPPPPPAVTQPPPPPASEPASNVGPPAQPTDSPQPQPQSEPPSQPTAPPPQQPSSPKAAPSSPAQQQKSAKQVITATNNGPVAITITSTSFVDTPTGKPNVNSANSNDNSGATGSIITAAIVVGTVVVAAAIGIWIFRKWKLTPSRNFKEKIQPVDFGPRSAESDEIFLRGLHEP

>Racas_AGL2_mira_a2_c7_228reads

MNITKNTFFLLLVCILFVSVFA

QQQPKQPPPSGGPDNA

PEPSGNPKGPGGPGEQSAPAGAPGPAATGPAQPGSSGTPPGPNAPGSSGAPGAPAPGPSN

APAGAPGASGAPASPTGASSPKGSEPAAAPKSSGASPAGAPKSSNAASTTAPAS

GAVSSVRFSGFELIVGSFAALFIGTLLY

(o) Amlep_oases (DNA, then protein)

# Oases part of Method 1 was unsuccessful for *A. leptoticha*

# **Method 2** was used to search Oases *k*-mers with 3 sequences Amlep_AGL1-AGL3

# identified from “getorf”, PG20%, ER positive sequences

# Amlep_AGL1-AGL3 were used to find additional sequences from the oases assemblies

>Amlep_AGL1_PG20_bAmlepk59Locus468rc_k69_43707_T1_489nt

aacataacaaaa

ATGGTACGAAAACACTTTTGCATTACTTTGTTCTTTGC

CATCACCATTGTAATGATTGCTACGCTAGTGAACGCACAATCTGGCAACG

GGAACGGAAACGGAAATGTTGGTAGTGGACATGGAAACGGTTATGGTAAT

GGTGGTTCATTAACAGGACAAGGAAATAATCAAGATAACGGAAATGCAGG

ATCTACTGGTGGTTCTGCTAATACTATAACGGAAATGCAGGATCTACTGG

TGGTTCTGCTAATACTACTACTGGAAGTTCTTCACCAAGTATTACGCAAA

GCGTTGCCACGCCTTCCTCCACTACAGCAAATGCTGCCGCTTCGCTTTCA

TATGACCTCACCAAGTATTACGCAAAGCGTTGCCACGCCTTCCTCCACTA

CAGCAAATGCTGCCGCTTCGCTTTCATATGACCTTCACTTTGGACGTGAA

GTTATTGGAATGCTCTTTGTCACTTTCGTCACTGCTTTGGTCGCTTTCTA

G

ggaatcactttgctcttggt

>Amlep_AGL2_PG20_bAmlepk59Locus468rc_k49_471_T12of16_357nt

acacagaatacataagaaaa

ATGT

TACGAAAGCACTTTTACATTACATTGTTCCTTGCCGTCACCATTGTAGCT

ATTGCTACGCTAGCGAACGCACAAGGTAGCGGGAACGGAAACGGAAACGG

AAATGTTGGTAGTGGTCACGGAAACGGCTATGGTAATGGTGGTTCATCAA

CAGGACAAGGAAATAATCAAGATAACGGAAATGGATCTAACGCCAGCACT

ACTGCAAGTTCGTCCGCAAGTGGTACGCAGAGCGCTGCCACTCCTTCCAC

TAGTACAAATGCTGCCGCTTCGCTTTCTTATGATCATCACTTTGGACGCG

AAGTTATTGGAATGCTCTTTGTCACTTTCATCACCGCTTTGGTTGCTTTC

TAG

agattttcgtcgtcattgga

>Amlep_AGL3_PG20_bAmlepk59Locus468rc_k49_8923_T2of10_654nt

atatcacttttttttaaaaa

ATGAGTCGACTCTCAGTATTTGTAATTTTTATGATGACT

TTAATCGCGTCCGTTTTATCTGATACCGCTCAAATAAACACACCTTCCAA

CCAAGTACAAGGTAGCATATATACTATCACATGGGCATACAATGGTACAA

ACGATGCAGTTGGAAGTCTCTCACTCAATAACAGAAATAATACTAGCAAT

GTCTTTACAATTGCCAAGCAAATTAAGTTAAGCCAACAATCTTATCAATG

GACTGTTGATCAAGATCCAGGAACTTATTATTTGACATTATCTTATCCTA

CGGGTAATGCTGAATCGGGAGATTTTCAGATTGTAAAAGGTGGTAGTGGT

AGCAATAATAATAACAATAACAATACTAGTAATAGCGGTAATAGTACCAA

TAGTACCAATAGTACCAATAGTACAAATAGTAACAATAGTACCAATAGCA

CCAATAGCACTAATAATAGTGATAATAGCACTATCACCTCTAGTCCTATT

CCAACAAACTCAACCGGATCTTCCGCTACTTCAACAAAACCTCCAAGCAA

CCGAAATACTGGCACACCTGGCGCGTCAAATAATGCAAACAAAATGGTTA

TTGATTCCTCTTTATTCACGACTGGTCTATTTTATTTGGTGCTTTCATCT

TTGCTAATAATCTAG

cataatttattatcaaagtt

>Amlep_AGL4_bAmlepAGL1_k49_L2120_T2of6_435nt

aacttaaaataaattctaac

ATGACA

AAAATCACGATCTTTACAGCTCTCGCTTACGCCATTCTCATCTTCGTTGCTTTGACCACT

GCCAGTCCTATTAAACGAGGTAACGACAATGGAAACGGTAATGGCAACGGAAACATAGGT

AGTGGCAATGGAAACGAAAATGGAAATTTCAACGCGGGTAGTTTAAATGGAAACGGTAAT

GGCAACGGAAACATTGGTAGCGGCAATGGAAACGAAAATGGAAATTTCAACGCGGGTAGT

TTTAATGGAAACTTGAATGGCAACGGAAACATAGGTAGCGCCAATGGAAACGAAAATGGA

AATTTCAACGTGGGTAGTTTAAATGGAAACTTGAATGGTAACGGAAACGTAGGCAGCGCC

AATGGAAACGAAAATGGAAATTTCAACATCGGTAGTGCTAATGGAAACAAAAATGGAAAT

GGCAACTAA

aagatgtatttattttcaca

>Amlep_AGL5_bAmlepAGL1_L4393_T2of2_323nt

gacttaaaataaattttaac

ATGACAAAAATCGCGATCTTTACAGCTCTCGCTTACGCCATTCTCATTTTT

GTTGCTTTGACCACTGCCAGTCCTATTAAACGAGGTGACGTCAATGGAAACGGTAATGGC

AACGGAAACGTAGGCAGTTTTAATGGAAACGGAAATGGCAACGGAAACGACGGTAGTCTT

AATGGAAACGGTAATGGCAACGGAAATGTAGGAAGTTTTAATGGTAACGTTAATGGCAAC

GGAAATGAAGGTAGCTTTAATGGTAACGAAAATGGCAACGGAAACGAAGGTAGTCTTAAT

GGAAACGTTAATGGCAACGGAAACGAAGGTAG

>Amlep_AGL6_bAmlepAGL6qm_k59_L22370_T1of1_624nt

tatccacaaataacgataaa

ATGAAAATCATTTTCAAGTTCATATTATTATCATGTCTCGTATTCT

CGATCATAATTACGAAATCAAATGCTGCGACGGGAACCACAGCCTCTGGATCTACAACTA

CGGCAACAACTTCTTTAGATTCTACCGCTTCTAAAACCAGTACGACGTCTTCTACATCAC

GATCAACTAATTCGCCAACTTCCACATCCACTACCACAAGTAGTAGCGCTAGTAGCAACA

CGAGTAGCAATAGTAATAGTAATAGTAACAATAATAGTAATAATAGTAATAATAGTAATA

ATAGTGGTAATGACAATAAAACTTCAGGAAATTCTCAACCTCAATCATCTCCTACTCCAC

AGACAACGACATCATTCAAGACTGTTGTATTATCCACTGTCGTCGATGGATCACAAAGTA

CCATCACAACGGTAACTCCGATCGCTGCGTCGCCAAACACAGAAACACAAGTCGTTACAG

TTGGTGCAGGTGGTCCAACTACTTCGCCTACTCAAAAGACTGTGGGCGGCTTGATTTCAT

ATGCAGAGAGAAATGATAACTTCACAATTTTGAGAAATGTTGTGGTTGGATTTGTGACGA

TTTTTGGAATGGGTTTCCTAGCTAATTTAAAGTATTAA

tatatgaacaaaattatata

>Amlep_AGL7_bAmlepAGL3_k49_L11677_T1of4_885nt

attaaataaagaatatagag

ATGAAGG

TCACATTTGTCAAAATTACTATTCTATTGTATATTGTTTGTGCGCTATTA

TTCGCGTTTTCTGCAGCCCAGCAAAACAATACTAGTAGCAGCAGCAGCAC

CAGCAGTGGTGGTGGTGGTAGTAGTAGTAGCAATAATAATAACGGAGGAA

CTAATAGCAATGGAAATAACAATAGTAACGCAAACAGCAACAACGGTAGT

AGCAACGGAAATAACAACGGCAGTAATAACAGCAATAACGGCAGTAATAA

CAGCAATAACAGCAATAGCAATAGTAATACTAATAATAGCAGTGATGGCA

GTAATAGCAGTAATAGTAACAGCACCTCAAATAATAACAACAATAATGAC

AATAGCAACAATAACAATGTTAATTCTGATGGAAGTATTACAATTAAAGT

TACTAAACCTAATAATGATGTGTTTTATGTGGGAACCTCTCATAATATTA

CGTGGGATATCCTATCTGCGATTCCACAAAGTCCTCCTTCCGTATCTATT

ATACTTCTACGCGGTGTCGCGAATGGTTCTCGTGTTATTTCCAATATCGC

TTCTGATGTGTCATTGACAGATAAAATCTATTTATGGAATATGCAAGATC

CATCACTTCAGAATGCATCTGATTACTCCGTACGAGTCTATAGTGGGGCA

CAAGTTAACGGATACTCTCCAACGTTTTCCATTATTAACACTCAAAACAC

CACCTCTCTGAGCAGTCCTTCGGGTGGTAATAGTGCCGCGAGTCCTAGCA

ATCCATCGACTTCTGTTTCTTCTAAACCAAATATTTTAAACGTTGGACCG

AAAACACAATCTTACATTTCAACTGCGTTCCTGTTAATCTTCACAGTTAT

TTTTGCTTCTTTCTGTTCATTCCAGTAA

attatcaggataaaattttg

>Amlep_AGL8_bAmlepAGL7_k49_L5858_T1of3_909nt

gttcttcctttcgatgggga

ATGGTATTAATGGCCATTTGCTTGTTCGTTTCTTTTGCTA

TGCAAGCTTCTGCAGAACCTTGGTGGAAGAGACAAAATAATAGTGGCACCAATAGTGGTA

ATAGTGGTAATAGTGGAAATAATGGCAGTAATGGTGGAGCGAGTAATGGCAATAGTAACA

GCAATCAGAACTCTGGTTCATCAAGCAGTAATAATAACAATAACAGTAATAACAGTAATA

ATAATAATAATAATAGCAACAACAATAGTAGTAACAATAACAGTAATAATAATGGTAATA

ATACATCATCATCATCATCATCATCATCATCAAATAATTCCACCAGCAACAATAACAATA

ATAGCTCTGCTACTCCAACCAACTCCACCTCATCTCCTAGCGGAACTTCAAGTGGCTCGA

GTTCGATAATTGTTGATCCATTAGTGGGACCAGCAATAATCCAAATGACAACACCGGCAG

TTACTCAAGGAAAAGCATTATACAAGATCGGAAGTCAAGTTACATTCGGATGGAAATACC

AAAGCACACCGATCATAAAACCTCAAGTTCTTAATCTTTTAACTCAAGCATCAACAAAAA

CTTGGTATACCATTGCTCAAAATATTTCTGCTTCGTCCACCTCATACATATGGGATACTT

CCAATCAAAAAAATCCCCCGCTGGTAATGGCCGATTACACTTTATATATCACTGATGAGC

GTGGTATAAATGCTCCAGCAACTGCCGGTAGATTAGAGCCATTCAATGGTCTAATATTCT

CATTATATTTACCGCAATCATATACCCCATTGGATGCGTATACTTGCGCAACTTGCTATA

GCGATGGCTCATTCCCATTAATTCCTATTGCTATGACATTTTCCGTGACGGTTATAACCG

TGATCTCTTTTAGTTTCTTTATGATCTGA

aaaacattgaaaatcatact

>Amlep_AGL1_PG20_bAmlepk59Locus468rc_k69_43707_T1_162aa

MVRKHFCITLFFAITIVMIATLVNA

QSGNGNGNGNVGSGHGNGYGNGGSLTGQGNN

QDNGNAGSTGGSANTITEMQDLLVVLLILLLEVLHQVLRKALPRLPPLQQMLPLRFHMTS

PSITQSVATPSSTTANA

AASLSYDLHFGREVIGMLFVTFVTALVAF

>Amlep_AGL2_PG20_bAmlepk59Locus468rc_k49_471_T12of16_118aa

MLRKHFYITLFLAVTIVAIATLANA

QGSGNGNGNGNVGSGHGNGYGNGGSSTGQGNNQDN

GNGSNASTTASSSASGTQSAATPSTSTNA

AASLSYDHHFGREVIGMLFVTFITALVAF

>Amlep_AGL3_PG20_bAmlepk59Locus468rc_k49_8923_T2of10_217aa

MSRLSVFVIFMMTLIASVLS

DTAQINTPSNQVQGSIYTITWAYNGTNDAVGSLSLNNRNN

TSNVFTIAKQIKLSQQSYQWTVDQDPGTYYLTLSYPTGNAESGDFQIVKGGSGSNNNNNN

NTSNSGNSTNSTNSTNSTNSNNSTNSTNSTNNSDNSTITSSPIPTNSTGSSATSTKPPSN

RNTGTPGASN

NANKMVIDSSLFTTGLFYLVLSSLLII

>Amlep_AGL4_bAmlepAGL1_k49_L2120_T2of6_144aa

MTKITIFTALAYAILIFVALTTA

SPIKRGNDNGNGNGNGNIGSGNGNENGNFNAGSLNGNGNGNGNIGSGNGNENGNFNAGSFNGNLNGNGNIGSANGNENGNFNVGSLNGNLNGNGNVGSANGNENGNFNIGSANGNKNGNGN

>Amlep_AGL5_bAmlepAGL1_L4393_T2of2_107aa

MTKIAIFTALAYAILIFVALTTA

SPIKRGDVNGNGNGNGNVGSFNGNGNGNGNDGSLNGNGNGNGNVGSFNGNVNGNGNEGSFNGNENGNGNEGSLNGNVNGNGNEG

>Amlep_AGL6_bAmlepAGL6qm_k59_L22370_T1of1_207aa

MKIIFKFILLSCLVFSIIITKSNA

ATGTTASGSTTTATTSLDSTASKTSTTSSTSRSTNSPTSTSTTTSSSASSNTSSNSNSNSNNNSNNSNNSNNSGNDNKTSGNSQPQSSPTPQTTTSFKTVVLSTVVDGSQSTITTVTPIAASPNTETQVVTVGAGGPTTSPTQKTVGGLISYAERNDNFTILRNVVVGFVTIFGMGFLANLKY

>Amlep_AGL7_bAmlepAGL3_k49_L11677_T1of4_294aa

MKVTFVKITILLYIVCALLFAFSAA

QQNNTSSSSSTSSGGGGSSSSNNNNGGTNSNGNNNSNANSNNGSSNGNNNGSNNSNNGSNNSNNSNSNSNTNNSSDGSNSSNSNSTSNNNNNNDNSNNNNVNSDGSITIKVTKPNNDVFYVGTSHNITWDILSAIPQSPPSVSIILLRGVANGSRVISNIASDVSLTDKIYLWNMQDPSLQNASDYSVRVYSGAQVNGYSPTFSIINTQNTTSLSSPSGGNSAASPSNPSTSVSSKPNILNVGPKTQSYISTAFLLIFTVIFASFCSFQ

>Amlep_AGL8_bAmlepAGL7_k49_L5858_T1of3_ 302aa

MVLMAICLFVSFAMQASA

EPWWKRQNNSGTNSGNSGNSGNNGSNGGASNGNSNSNQNSGSSSSNNNNNSNNSNNNNNNSNNNSSNNNSNNNGNNTSSSSSSSSSNNSTSNNNNNSSATPTNSTSSPSGTSSGSSSIIVDPLVGPAIIQMTTPAVTQGKALYKIGSQVTFGWKYQSTPIIKPQVLNLLTQASTKTWYTIAQNISASSTSYIWDTSNQKNPPLVMADYTLYITDERGINAPATAGRLEPFNGLIFSLYLPQSYTPLDAYTCATCYSDGSFPLIPIAMTFSVTVITVISFSFFMI

(p) Amlep_mira (DNA, then protein)

# Amlep_AGL1_mira missing 3nt ATG at start (but still encodes ER signal sequence)

# and 41nt gap

# use Oases seq

>Amlep_AGL1_mira_a2_c11_36reads_369nt

GTACGAAAACACTTTTGCATTACTTTGTTCTTTGCCATCACCATTGTAAT

GATTGCTACGCTAGTGAACGCACAATCTGGCAACGGGAACGGAAACGGAA

ATGTTGGTAGTGGACATGGAAACGGTTATGGTAATGGTGGTTCATTAACA

GGACAAGGAAATAATCAAGATAACGGAAATGCAGGATCTACTGGTGGTTC

TGCTAATACTACTACTGGAAGTTCTTCACCAAGTATTACGCAAAGCGTTG

CCACGCCTTCCTCCACTACAGCAAATGCTGCCGCTTCGCTTTCATATGAC

CTTCACTTTGGACGTGAAGTTATTGGAATGCTCTTTGTCACTTTCGTCAC

TGCTTTGGTCGCTTTCTAG

# Amlep_AGL2_mira is in two contigs

# N-term has big deletion compared to oases – no stop or GPI

# C-term has GPI-signal, but not ATG or ER – other differences

# use oases

>Amlep_AGL2_mira_nterm_a2_c7rc_123reads_402nt

ATGTTACGAAAGCACTTTTACATTACATTGTTCCTT

GCCGTCACCATTGTAGCTATTGCTACGCTAGCGAACGCACAAGGTAGCGGGAACGGAAAC

GGAAACGGAAATGTTGGTAGTGGTCACGGAAACGGCTATGGTAATGGTGGTTCATCAACA

GGACAAGGAAATAATCAAGATAACGGAAATGGATCTAACGGTAATGGTTCTAATGGTGGT

GCCGGTGGTATTGGTACTAATGGTGCTAATGGTGCTAATGGTTCTAATGGTGGTTCGGGT

ATTGGTACTAATGGTGCTAATGGTGCTAATGGTTCTAATGGTGGTTCGGCCAGCACTACT

GCAAGTTCGTCCGCAAGTGGTACGCAGAGCGCTGCCACTCCTTCCACTAGTACAAATGCT

GCCGCT

>Amlep_AGL2_cterm_mira_a2_c8_41reads_310nt

GGTATCAACGCAGAGTACGGGGAACGCACAAGGTAGCGGGAACGGAAACG

GAAACGGAAATGTTGGTAGTGGTCACGGAAACGGCTATGGTAATGGTGGT

TCATCAACAGGACAAGGAAATAATCAAGATAACGGAAATGGATCTAACGC

CAGCACTACTGCAAGTTCGTCCGCAAGTGGTACGCAGAGCGCTGCCACTC

CTTCCACTAGTACAAATGCTGCCGCTTCGCTTTCTTATGATCATCACTTT

GGACGCGAAGTTATTGGAATGCTCTTTGTCACTTTCATCACCGCTTTGGT

TGCTTTCTAG

# Amlep_AGL3_mira CDS same as OASES

>Amlep_AGL3_mira_a2_c1_915reads_654nt

# Amlep_AGL4_mira is 71nt shorter than oases seq (and has 5 SNPs)

# only 12 reads – go with oases

>Amlep_AGL4_mira_a1_c12rc_12reads_176nt

ATGACAAAAATCACGATCTTTACAGCTCTCGCTTACGCCATTCTCATCTTCG

TTGCTTTGACCACTGCCAGTCCTATTAAACGAGGTAACGACAATGGAAACGGTAATGGCA

ACGGAAACATAGGTAGTGGCAATGGAAACGAAAATGAGAATGGCGTAAGCGAGAGCTGTA

AAGA

# Amlep_AGL5 Oases sequence is 46 nt longer, with some mis-matches near the 3’ end

# Use mira sequence

>Amlep_AGL5_mira_a1_c6rc_66reads_279nt

ATGACAAAAATCGCGATCTTTACAGCTCTCGCTTACGCCATTCTCATTTT

TGTTGCTTTGACCACTGCCAGTCCTATTAAACGAGGTGACGTCAATGGAAACGGTAATGG

CAACGGAAACGTAGGCAGTTTTAATGGAAACGGAAATGGCAACGGAAACGACGGTAGTCT

TAATGGAAACGGTAATGGCAACGGAAATGTAGGAAGTTTTAATGGTAACGGAAATGGCAA

CGGAAACGAAAGGAAGTTTTAA

tgaaaacgcgaatgggaatggcaataa

# Amlep_AGL6_mira CDS same as OASES

>Amlep_AGL6_mira_a1_c4_202reads_624nt

# Amlep_AGL7_mira CDS same as OASES

>Amlep_AGL7_mira_a2_c3_333reads_885nt

# Amlep_AGL8_mira CDS same as OASES

>Amlep_AGL8_mira_a1_c3_393reads_909nt

>Amlep_AGL5_mira_a1_c6rc_66reads_83aa

MTKIAIFTALAYAILIFVALTTA

SPIKRGDVNGNGNGNGNVGSFNGNGNGNGNDGSLNGNGNGNGNVGSFNGNGNGNGNERKF

# Amlep_AGL1-AGL3 were used to find Pabra sequences from the Oases assemblies

(q) Pabra_oases (DNA, then protein)

>Pabra_AGL1_bAmlep2_k59_14541_T3of3_556nt

aacttaaaataaattctaac

ATGACAAAAATCACGA

TCTTTACAGCTCTCGCTTACGCCATTCTCATCTTCGTTGCTTTGACCACTGCCAGTCCTA

TTAAACGAGGTAACGACAATGGAAACGGTAATGGCAACGGAAACATAGGTAGTGGCAATG

GAAACGAAAATGGAAATTTCAACGCGGGTAGTTTAAATGGAAACGGTAATGGCAACGGAA

ACATTGGTAGCGGCAATGGAAACGAAAATGGAAATTTCAACGCGGGTAGTTTTAATGGAA

ACTTGAATGGCAACGGAAACATAGGTAGCGCCAATGGAAACGAAAATGGAAATTTCAACG

TGGGTAGTTTAAATGGAAACTTGAATGGTAACGGAAACGTAGGCAGCGCCAATGGAAACG

AAAATGGAAATTTCAACATCGGTAGTGCTAATGGAAACAAAAATGGAAATGGCAACTAAA

AGATGTATTTATTTTCACAAACACCTTATTCTCCGGACATTAATTATCTGAGGTGTTAAT

CGTTGAATAATAGGTTGTAGCTGTATATTAGCTGTCTCTTATACACATCTGACGCTGCCG

>Pabra_AGL2_bAmlep1_k59_Locus_7326_T1of2_481nt

gacccattaaaaaaatcata

ATGTCTGGAAAATTTGTAACATTGATT

TATGCTGCTCTTTTATCAATTGCCTTGACAACCGCAGCTCCAGTACTAGAAAAACGGGGT

AATTATAATGGCAATGGTAATGGAAATGGAAACGTAGGAAGTGGAAATGGAAACCTTAAT

GGCAACTTCAACGACGGATCTTTCAATGGTAACAACAACGGAAATTTCAATTGGGGATCT

GGCAATGGCAACGCGAATGGTAATCTTAACGACGGATCGTTTAATGGTAACAGCAACGGA

AATTACAATGTGGGATATTATAATGGCAACGGGAATGGTAATTTAAACGACGGCTCATTC

AATGGAAATAACAACGGAAATTGGAATTGGGGATCTGCTAATGGCAACGCGAATGGTAAT

GGAAACCATAAACGTGGTGATTACAATGGCAATGGCAATGGAAATGGAAACGTAGGAAGT

GGAAATGGAAACCTTAATGGCAACTTCAACGACG

>Pabra_AGL3_bpabra2_k69_650_T1of2_420nt

gaataacaacgggtctaata

ATGGGAATTGT

AATTCGAATAGTAGTAATAATGGAAATGGCTTTGGAAATGGAGTTACTGG

TGACAATCAGGGGAATAATAATGGTAATGGGAATTGTGGGATTAATAATA

CTTCTAGTGGTAGTAATGGAACTGGAAGTTAATGGAAATGGCTTTGGAAA

TGGAGTTACTGGTGACAATCAGGGGAATAATAATGGTAATGGGAATTGTG

GGATTAATAATACTTCTAGTGGTAGTAATGGAACTGGAAGTAGGAACAAT

AGTACTAATAATTCACCAAATAATTCATCAAATACGAGTACATCGAATAC

AAGTTCACCGAATACGAGCTCGGGCGTTGCATATTGGGAAAAATGTTTTG

GAATATTTGAAGTATTGAGCCTAGTCTTTATCGGGATGGGTATACAATCA

GTTATTTGA

aagttgacataaatgtttat

>Pabra_AGL4_bpabra3_713_T1of1_831nt

aaacaacaagcttaccaaaa

ATGCTTAATAAATCGGTCAAGTTTATCCTTTTTCTAG

TTCTTACTGCGACTGTTTTAAGTATCTTATCAAAACAAATATCTCGGGAT

GTTGTCGCTGCCGAACTGATAAATGAAAATTATATAAGATCCTTCAACGA

AATTGTGAAAGTCAAAAAACGTCAAACGAACGGACAGAATAACGGAGACG

CTTCAAAAAATAACGGGCAGCATAACGGACAAAATAGTACTGGCGTAAAT

AACGGCAATAACAACGGTAATAGTCCTTTTGGTTTTGGAAATGGCAACAA

CAACGGAAATAACAGCACCGGATCAAATAACGGAAACAACAATGGAAACG

GCGACATCGGATCAAATAACGGGAACAACAACGGAAACGGCAGCATCGGA

TCAAATAATGGAAACAATAATGGAAACAACAACAAAGGGTCAGGTAATGG

TAGCAACAACGGAAACAACAATATCGGTTCAAATAATGGAAACGGAAACG

GGAACAATAATAAAGGGAATAACAACGGGTCTAATAATGGGAATTGTAAT

TCGAATAGTAGTAATAATGGAAATGGCTTTGGAAATGGAGTTACTGGTGA

CAATCAGGGGAATAATAATGGTAATGGGAATTGTGGGATTAATAATACTT

CTAGTGGTAGTAATGGAACTGGAAGTAGGAACAATAGTACTAATAATTCA

CCAAATAATTCATCAAATACGAGTACATCGAATACAAGTTCACCGAATAC

GAGCTCGGGCGTTGCATATTGGGAAAAATGTTTTGGAATATTTGAAGTAT

TGAGCCTAGTCTTTATCGGGATGGGTATACAATCAGTTATTTGA

aagttgacataaatg

>pabra_AGL1_bAmlep2_k59_14541_T3of3_144aa

MTKITIFTALAYAILIFVALTTA

SPIKRGNDNGNGNGNGNIGSGNGNENGNFNAGSLNGN

GNGNGNIGSGNGNENGNFNAGSFNGNLNGNGNIGSANGNENGNFNVGSLNGNLNGNGNVG

SANGNENGNFNIGSANGNKNGNGN

>pabra_AGL2_bAmlep1_k59_Locus_7326_T1of2_160aa

MSGKFVTLIYAALLSIALTTA

APVLEKRGNYNGNGNGNGNVGSGNGNLNGNFNDGSFNGNNNGNFNWGS

GNGNANGNLNDGSFNGNSNGNYNVGYYNGNGNGNLNDGSFNGNNNGNWNWGSANGNANGN

GNHKRGDYNGNGNGNGNVGSGNGNLNGNFND

>pabra_AGL3_bpabra2_k69_650_T1of2_139aa

MGIVIRIVVIMEMALEMELLVTIRGIIMVMGIVGLIILLVVVMELEVNG

NGFGNGVTGDNQGNNNGNGNCGINNTSSGSNG

TGSRNNSTNNSPNNSSNTSTSNTSSPNTS

SGVAYWEKCFGIFEVLSLVFIGMGIQSVI

>pabra_AGL4_bpabra3_713_T1of1_276aa

MLNKSVKFILFLVLTATVLS

ILSKQISRDVVAAELINENYIRSFNEIVKVKKRQTNGQNNGDASKNNGQHNGQNST

GVNNGNNNGNSPFGFGNGNNNGNNSTGSNNGNNNGNGDIGSNNGNNNGNGSIGSNNGNNN

GNNNKGSGNGSNNGNNNIGSNNGNGNGNNNKGNNNGSNNGNCNSNSSNNGNGFGNGVTGD

NQGNNNGNGNCGINNTSSGSNGTGSRNNSTNNSPNNSSNTSTSNTSSPNTS

SGVAYWEKCFGIFEVLSLVFIGMGIQSVI

(r) Pabra_mira (DNA, then protein)

# Pabra_AGL1_mira is 224nt shorter that the oases sequence

# still partial sequence, no stop codon

# Use mira sequence

>Pabra_AGL1_mira_a1_c4_44reads_332nt

ATGACAAAAATCACGATCTTTACAGCTCTCGCTTACG

CCATTCTCATCTTCGTTGCTTTGACCACTGCCAGTCCTATTAAACGAGGTAACGACAATG

GAAACGGTAATGGCAACGGAAACATAGGTAGTGGCAATGGAAACGAAAATGGAAATTTCA

ACGCGGGTAGTTTAAATGGAAACGGTAATGGCAACGGAAACATTGGTAGCGGCAATGGAA

ACGAAAATGGAAATTTCAACGCGGGTAGTTTTAATGGAAACTTGAATGGCAACGGAAATA

TAGGTAACGGCAATGGAAACGAAAATGGAAATTTCAACGCGGGTAGTTTTAATGG

# Pabra_AGL2_mira is 481nt longer

# still partial sequence, no stop codon

# No GPI-anchor predicted

>Pabra_AGL2_mira_a1_c2_175reads_890nt

ATGTCTGGAAAATTTGTAACAT

TGATTTATGCTGCTCTTTTATCAATTGCCTTGACAACCGCAGCTCCAGTA

CTAGAAAAACGGGGTAATTATAATGGCAATGGTAATGGAAATGGAAACGT

AGGAAGTGGAAATGGAAACCTTAATGGCAACTTCAACGACGGATCTTTCA

ATGGTAACAACAACGGAAATTACAATTGGGGATCTGGCAATGGCAACGCG

AATGGTAATCTTAACGACGGTTCGTTCAATGGTAATAGCAACGGAAATTA

CAATGTGGGATATTATAATGGCAACGCGAATGGTAATTTAAACGACGGCT

CATTCAATGGAAATAACAACGGAAATTGGAATTGGGGATCTGCTAATGGC

AACGCGAATGGTAATGGAAACCATAAACGTGGTGATTACAATGGCAATGG

CAATGGAAATGGAAACGTAGGAAGTGGAAATGGAAACCTTAATGGCAACT

TCAACGACGGATCGTTCAATGGTAACAACAACGGAAATTTCAATTGGGGA

TCTGGCAATGGCAACGCGAATGGTAATCTTAACGACGGATCGTTCAATGG

TAACAGCAACGGAAATTACAATGTGGGATATTATAATGGCAACGCGAATG

GTAATTTAAACGACGGCTCATTCAATGGAAATAACAACGGAAATTGGAAT

TGGGGATCTGCTAATGGCAACGCGAACGGTAATTTAAACCATAAACGTGG

TAATTACAATGGCAATGGCAATGGAAATGGAAACGTAGGAAGTGGAAATG

GAAACCTTAATGGCAACTTCAACGACGGATCGTTCAATGGTAACAACAAC

GGAAATTTCAATTGGGGATCTGGCAATGGCAATGCGAATGGTAATCTTAA

CGACGGATCGTTCAATGG

# Pabra_AGL3 is a truncated version of Pabra_AGL4, that includes

# a frameshift and an insertion, in the GN rich regions, that results in an ORF

# that starts with Met – compare Frame 1 and Frame 3 below

# sequence in blue matches Pabra_AGL4

# 5'3' Frame 1_same as Oases sequence

# MGIVIRIVVIMEMALEMELLVTIRGIIMVMGIVGLIILLVVVMELEVNGNGFGNGVTGDN

# QGNNNGNGNCGINNTSSGSNGTGSRNNSTNNSPNNSSNTSTSNTSSPNTSSGVAYWEKCF

# GIFEVLSLVFIGMGIQSVI-

# 5'3' Frame 3_has Pabra_AGL4

# GNCNSNSSNNGNGFGNGVTGDNQGNNNGNGNCGINNTSSGSNGTGS-WKWLWKWSYW-QS

# GE--W-WELWD--YF-W--WNWK-EQ-Y--FTK-FIKYEYIEYKFTEYELGRCILGKMFWNI

# -SIEPSLYRDGYTISYL

>Pabra_AGL4_mira_a1_c1_942reads_831nt

ATGCTTAATAAA

TCGGTCAAGTTTATCCTTTTTCTAGTTCTTACTGCGACTGTTTTAAGTAT

CTTATCAAAACAAATATCTCGGGATGTTGTCGCTGCCGAACTGATAAATG

AAAATTATATAAGATCCTTCAACGAAATTGTGAAAGTCAAAAAACGTCAA

ACGAACGGACAGAATAACGGAGACGCTTCAAAAAATAACGGGCAGCATAA

CGGACAAAATAGTACTGGCGTAAATAACGGCAATAACAACGGTAATAGTC

CTTTTGGTTTTGGAAATGGCAACAACAACGGAAATAACAGCACCGGATCA

AATAACGGAAACAACAATGGAAACGGCGACATCGGATCAAATAACGGGAA

CAACAACGGAAACGGCAGCATCGGATCAAATAATGGAAACAATAATGGAA

ACAACAACAAAGGGTCAGGTAATGGTAGCAACAACGGAAACAACAATATC

GGTTCAAATAATGGAAACGGAAACGGGAACAATAATAAAGGGAATAACAA

CGGGTCTAATAATGGGAATTGTAATTCGAATAGTAGTAATAATGGAAATG

GCTTTGGAAATGGAGTTACTGGTGACAATCAGGGGAATAATAATGGTAAT

GGGAATTGTGGGATTAATAATACTTCTAGTGGTAGTAATGGAACTGGAAG

TAGGAACAATAGTACTAATAATTCACCAAATAATTCATCAAATACGAGTA

CATCGAATACAAGTTCACCGAATACGAGCTCGGGCGTTGCATATTGGGAA

AAATGTTTTGGAATATTTGAAGTATTGAGCCTAGTCTTTATCGGGATGGG

TATACAATCAGTTATTTGA

# Use Pabra_AGL1_mira sequence even though shorter than Oases since so repetitive

>Pabra_AGL1_mira_a1_c4_44reads_110aa

MTKITIFTALAYAILIFVALTTA

SPIKRGNDNGNGNGNGNIGSGNGNENGNFNAGSLNGNGNGNGNIGSGNGNENGNFNAGSFNGNLNGNGNIGNGNGNENGNFNAGSFN

# Pabra_AGL2_mira is 136aa longer, still not full length or GPI-anchored

>Pabra_AGL2_mira_a1_c2_175reads_296aa

MSGKFVTLIYAALLSIALTTA

APVLEKRGNYNGNGNGNGNVGSGNGNLNGNFNDGSFNGNNNGNYNWGSGNGNANGNLNDGSFNGNSNGNYNVGYYNGNANGNLNDGSFNGNNNGNWNWGSANGNANGNGNHKRGDYNGNGNGNGNVGSGNGNLNGNFNDGSFNGNNNGNFNWGSGNGNANGNLNDGSFNGNSNGNYNVGYYNGNANGNLNDGSFNGNNNGNWNWGSANGNANGNLNHKRGNYNGNGNGNGNVGSGNGNLNGNFNDGSFNGNNNGNFNWGSGNGNANGNLNDGSFN

# Pabra_AGL4_mira CDS same as OASES

>Pabra_AGL4_mira_a1_c1_942reads_276aa

(s) Gimar NCBI WGS/TSA BLASTn (gDNA, cDNA then protein, see Table S7 for accessions)

>Gimar_AGL1_gDNA

ATGATTCTCACTAAAAATACGATTATTCTCTTCTTCGTGTGTATTTTTAT

CGCCTCTGGCGTTTTTGCTCAAGAgtaagtgtttaaagttagtaactttctcaattttttttctctaaat

atgtttcatactttttctataatcccacagTGCTACTTCCTCCGCCGCCGCTCCCGGTGGCAAGGCTCCT

CCAACTCCTAGTGGTGCTGCTCCTGCTGATGCTACTAGTTCTCCTACTGGTGGTGGTGCTGGTGGTGCTG

CTCCTAGTGGCGCTGCTCCCGCTGGTGGTGCCGGTGGTCCTCCTCCTGCCGGTGCTTCTCCTGCTCCAAG

TGGCGCTGCTCCCGCCGGTGGTGCCGGTCCTGCTCCTTCTCCTGCTCCAAGTGGTGCTGCTAAGGGCGCT

GGgtaagtatttttaagctttctgttaattatatttgcaataacaataattgacaatatctttttttttt

tttttttaaaaaaaaaaaagTGCTTCTGGATCTGCAAAAGCTCCAAGTAGTTCTGGAGCTGCTGCTGCTT

CCCCAGCAGCCACATCATCCCCTGGTGCCGCAAAAGGTGCCGCTTCAAGCGTTCGCGTTGACAGTTTTGA

AATGATCATTGGTTCTTGTGCTGCTTTAATCATTGGTGGTTTATTATATTAA

>Gimar_AGL1_TSA

ATGATTCTCACTAAAAATACGATTATTCTCTTCTTCGTGTGTATTTTTATCGCCTCTGGCG

TTTTTGCTCAAGATGCTACTTCCTCCGCCGCCGCTCCCGGTGGCAAGGCTCCTCCAACTCCTAGTGGTGC

TGCTCCTGCTGATGCTACTAGTTCTCCTACTGGTGGTGGTGCTGGTGGTGCTGCTCCTAGTGGCGCTGCT

CCCGCTGGTGGTGCCGGTGGTCCTCCTCCTGCCGGTGCTTCTCCTGCTCCAAGTGGCGCTGCTCCCGCCG

GTGGTGCCGGTCCTGCTCCTTCTCCTGCTCCAAGTGGTGCTGCTAAGGGCGCTGGTGCTTCTGGATCTGC

AAAAGCTCCAAGTAGTTCTGGAGCTGCTGCTGCTTCCCCAGCAGCCACATCATCCCCTGGTGCCGCAAAA

GGTGCCGCTTCAAGCGTTCGCGTTGACAGTTTTGAAATGATCATTGGTTCTTGTGCTGCTTTAATCATTG

GTGGTTTATTATATTAA

>Gimar_AGL2_gDNA

ATGTCACGACTAATTTA

TTACCTTACACTACTGTTCTTGGTTTTCTTGGTTTTTATTATGTCTGCTACATCCCgtaa

gtcgataaaatttatgctatttagagcgaatttgataaaaagattatataaaagtaatca

ataatcaattataagctaaacagagaagttgtttggataatccagtagcttcttagtttt

tgattataaattattgcttgattaaaaaaaattcagtatagataattctaagttaattcg

aattataataatttaaaccaattttaacatgatgcaatacatacgtacctatattttcag

AGGGGCCTGGTGGTGCTGGTCCTGCTGGTGGTCCTGGTGGTGCTGGTACTGGTGGTCCTG

GTGGTGCTGGTGCTGGTGGTCCTGGTGGTGCTGCTCCTACTGGTGGTCCTGGTGGTCCTG

GTGGTGCTGGTCCTGGTGGTCCTGGTGGTGCTGGTGCTGGTGGTCCTGGTGGTCCTAGTG

GTGCTGCTCCTGCTGGTCCTTCTCCTAGTGTTGCTGTTGGTGCTCCTGCTGGTCCTGGTC

CTGCTGGTGCTCCTGGTGCTCCTACTGCTGCTGGTCCTGCTTCTGCTCCAACTCCGGCGG

GTCCTGTTGGTGGTGCTCCTGCTCCAACTCCGTCTGCTGTTTCTATTATTACTTTTACAG

TTACGGCTCAACCTACACTTGTCAGTGCTAATGCACCTGTCCCTACTGCCACACACCCCA

CCCTACAAACTTCAGAACAATCTTCTTCACTTAATATTGGAATGTATGTTGGTATTGCTA

TAGGTGGTGGTGTTGTAGGTGGTGTTGTAGTTGGTATAATCTTATCTTTTGTTTGGTATC

GTGTATATAAAAAACATTTAGATTTACAATTTGTTCCAACACCTGGCAGCGTTAATATTT

AG

>Gimar_AGL2_TSA

ATGTCACGACTAATTTAT

TACCTTACACTACTGTTCTTGGTTTTCTTGGTTTTTATTATGTCTGCTACATCCCAGGGG

CCTGGTGGTGCTGGTCCTGCTGGTGGTCCTGGTGGTGCTGGTACTGGTGGTCCTGGTGGT

GCTGGTGCTGGTGGTCCTGGTGGTCCTAGTGGTGCTGCTCCTGCTGGTCCTTCTCCTAGT

GTTGCTGTTGGTGCTCCTGCTGGTCCTGGTCCTGCTGGTGCTCCTGGTGCTCCTACTGCT

GCTGGTCCTGCTTCTGCTCCAACTCCGGCGGGTCCTGTTGGTGGTGCTCCTGCTCCAACT

CCGTCTGCTGTTTCTATTATTACTTTTACAGTTACGGCTCAACCTACACTTGTCAGTGCT

AATGCACCTGTCCCTACTGCCACACACCCCACCCTACAAACTTCAGAACAATCTTCTTCA

CTTAATATTGGAATGTATGTTGGTATTGCTATAGGTGGTGGTGTTGTAGGTGGTGTTGTA

GTTGGTATAATCTTATCTTTTGTTTGGTATCGTGTATATAAAAAACATTTAGATTTACAA

TTTGTTCCAACACCTGGCAGCGTTAATATTTAG

>Gimar_AGL3_gDNA

ATGAATCTTTCTAAGAACACAATCATCTTCGTTCTCT

TATGCATCCTTATCGTCTCTAATGTTTTAGCTCAGGGgtaagtaaattttcaaagtcgtt

tttaacatcgaaaattcaattttattatatttttttcattatttttaaaatataatttct

attttttcttgttaaatctaaaagCCCAACCGCTACTGGTGAAGGTGGACCTGGTGGACC

TGCTCCAACTGGTGCTGGTGGACCTGGTGGACCTGGTGCTGGTGGACCTGGTGGACCTGG

TGCTGGTGGACCTGGTGGTGCCGGTCCATCTGGTGCACCTGCTAGCCCAAGTGGTGGACC

TGGTGGACCTGGTGGTGCTGGACCTGCTGGTGGACCTGGTGGTGCTGGTCCATCTGGCGC

ACCTGGTGCACCTGGTGCTGGAGCTACTCCCGCTGCTGGACCTGGAGGTGCTGCACCATC

TGGTGCTGCTGGATCTGGTGCTCCTGCTTCTGGACCTGGTGCAACTCCTGCTGCTGCTGG

ACCTGGCGCATCTCCTGCTGCATCACCAAAATCTCCTGCTGGAGCTTCTGGAGCTGCCCC

AAGTGGTACTGGCGCTGCTAAAGGAGCAAGCCCgtaagtatattttaataaaatttgttt

ttttaataaattttttttttttttaataattattttaattaattcatttattaattatta

tatttcaattacatttttaaagATCTACTTCACCCGCAGCTGGTGCCACTGGTGCTGCCT

CAAATGTTCATGTTAACTGCTTTGAATTGATCACTGGATCCGTTATTGCTTTATTCTTTG

GCACATTATTATATTAA

>Gimar_AGL3_TSA

ATGAATCTTTCTAAGA

ACACAATCATCTTCGTTCTCTTATGCATCCTTATCGTCTCTAATGTTTTAGCTCAGGGCCCAACCGCTAC

TGGTGAAGGTGGACCTGGTGGACCTGCTCCAACTGGTGCTGGTGGACCTGGTGGACCTGGTGCTGGTGGA

CCTGGTGGTGCCGGTCCATCTGGTGCACCTGCTAGCCCAAGTGGTGGACCTGGTGGACCTGGTGGTGCTG

GACCTGCTGGTGGACCTGGTGGTGCTGGTCCATCTGGCGCACCTGGTGCACCTGGTGCTGGAGCTACTCC

CGCTGCTGGACCTGGAGGTGCTGCACCATCTGGTGCTGCTGGATCTGGTGCTCCTGCTTCTGGACCTGGT

GCAACTCCTGCTGCTGCTGGACCTGGCGCATCTCCTGCTGCATCACCAAAATCTCCTGCTGGAGCTTCTG

GAGCTGCCCCAAGTGGTACTGGCGCTGCTAAAGGAGCAAGCCCATCTACTTCACCCGCAGCTGGTGCCAC

TGGTGCTGCCTCAAATGTTCATGTTAACTGCTTTGAATTGATCACTGGATCCGTTATTGCTTTATTCTTT

GGCACATTATTATATTAA

>Gimar_AGL4_gDNA

ATGAATTTCACTAAGAATACAATCCTTTTCCTTGTCTTATGCATCCTTATCGTCTCTAGTGTACTTGCTC

AAAGgtatgttagttttcaaagccatttagcatcgaaaattcaatatttttttcactatttttaaaatat

atttgcactctcctgttaactctagCCCCAGTGCACCTGCTGGTGGAGCTGCCCCAACGGAATCTGGTGC

CGCTGAAACTGGTGCCCCAAGCCCTAGTGGAGCAGCAAAAGGTCCAGCACCTTCTGGAGCCCCCCCAGCT

GGCGCTGCACCATCAGGAGCTGGCGCTGCTGCACCATCAGGAACTGGTGCCGCTGCTGCACCTGCCGGAA

GTGCCGCCCCAAGTGGAGCAGCAAAAGGTCCAGCACCTTCTGGAGCCCCATCTGGTGGTTCACCAAGTGG

TTCCGGTAAAGCACCAAGCTCTTCTGGgtaagtatttttataaacctttataattattttaattaatttt

tatttattaattattatatgacaattgcctttttaaagAACTTCACCTGCCACAGCAGCATCTAGCAGTC

CTGCCAAATCTGGTGCTTCAAGCATTCGTGTTGAATTGTTCGCTGGATCCGTTATAGCTTTATTCTGTGG

TGCATTGTTATATTAA

>Gimar_AGL4_TSA

ATGAATTTCACTAAGAATACAATCCTTTTCCTTGTCTTATGCATCCTTATCGTCTCTA

GTGTACTTGCTCAAAGCCCCAGTGCACCTGCTGGTGGAGCTGCCCCAACGGAATCTGGTGCCGCTGAAAC

TGGTGCCCCAAGCCCTAGTGGAGCAGCAAAAGGTCCAGCACCTTCTGGAGCCCCCCCAGCTGGCGCTGCA

CCATCAGGAGCTGGCGCTGCTGCACCATCAGGAACTGGTGCCGCTGCTGCACCTGCCGGAAGTGCCGCCC

CAATCTGGTGGTTCACCAAGTGGTTCCGGTAAAGCACCAAGCTCTTCTGGAACTTCACCTGCCACAGCAG

CATCTAGCAGTCCTGCCAAATCTGGTGCTTCAAGCATTCGTGTTGAATTGTTCGCTGGATCCGTTATAGC

TTTATTCTGTGGTGCATTGTTATATTAA

>Gimar_AGL1

MILTKNTIILFFVCIFIASGVFA

QDATSSAAAPGGKAPPTPSGAAPADATSSPTGGGAGG

AAPSGAAPAGGAGGPPPAGASPAPSGAAPAGGAGPAPSPAPSGAAKGAGASGSAKAPSSS

GAAAASPAATSSPGAAKGAASSVRVDSFEMIIGSCAALIIGGLLY

>Gimar_AGL2

MSRLIYYLTLLFLVFLVFIMSATS

QGPGGAGPAGGPGGAGTGGPGGAGAGGPGGAAPTGG

PGGPGGAGPGGPGGAGAGGPGGPSGAAPAGPSPSVAVGAPAGPGPAGAPGAPTAAGPASA

PTPAGPVGGAPAPTPSAVSIITFTVTAQPTLVSANAPVPTATHPTLQTSEQSSSLNIGMY

VGIAIGGGVVGGVVVGIILSFVWYRVYKKHLDLQFVPTPGSVNI

>Gimar_AGL3

MNLSKNTIIFVLLCILIVSNVLA

QGPTATGEGGPGGPAPTGAGGPGGPGAGGPGGPGAGG

PGGAGPSGAPASPSGGPGGPGGAGPAGGPGGAGPSGAPGAPGAGATPAAGPGGAAPSGAA

GSGAPASGPGATPAAAGPGASPAASPKSPAGASGAAPSGTGAAKGASPSTSPAAGATG

AASNVHVNCFELITGSVIALFFGTLLY

>Gimar_AGL4

MNFTKNTILFLVLCILIVSSVLA

QSPSAPAGGAAPTESGAAETGAPSPSGAAKGPAPSGA

PPAGAAPSGAGAAAPSGTGAAAAPAGSAAPSGAAKGPAPSGAPSGGSPSGSGKAPSSSGT

SPATAASSSPAKS

GASSIRVELFAGSVIALFCGALLY

(t) Giros NCBI WGS BLASTn (gDNA then protein, see Table S7 for accessions)

>Giros_AGL1_gDNA

ATGATTATCACTAAAAATACGATTATTCTCTTCTTCGTGTGTATTCTTATCGCCTCTGGC

GTTTTTGCCCAAGATGAgtaagtgtttaaagttaataactttgaagctctcaattttttt

tctctaaatatgtttcatacttttttgtaatcccacagGACTACTTCCCCCGCCGCCGGT

GCTGATGCTACCGGTGCTGGTGGTGCTGGTGCTGCTCCTACCGGCGGTGCTGGTGGCGCT

GCTCCCAAAGGTGGTGCTGCTCCTACCGGCGATGCTGGTGGCGCTGCTCCCAAAGGTGGT

GCTGCTCCTACTTCTAGTGGTGCTGCTCCCGCTGCTAGTGGTGGTGCTGCTCCCGCTGCT

AGTGGTGGTGCTGCTCCCGCCGGTGCTGGTGGTGCTGCTCCAAGTGCTTCTCCTAGTGGT

GGTGCTGCTAAGGGCGCTGGgtaagtatttttaagctttctgttaattatatttgcaata

attgactattttttttttttttttaaaaaaaaaaaa

>Giros_AGL2_gDNA

ATGTCACGACTAATTTATTGCCTTACACTACTGTTCTTGGTTTTCTTGGTTTTTATTAGG

TCTGCTACATCCCgtaagtcaacaaaatttatgctatttaaagcgaatttgataaaaaga

ttatatagaataatcattattataagctaaacagggatgttgtttggatattacagcaac

atattaatttttgattataaattattgctgattaaaaaaatttcgtatagataattctaa

gtaaaaacagttaattcaattaaaatatattaaagcccattttaacatgatgcaatacgt

acgtacctatattttcagAGAATTCTTCTAAGGGTGCTGCTACTCCAGCAGCTGGTCCAG

CAGGTGCTGCTAGTCCTGTTCCAACTCCGGCGAGTGCTGCTGCTCCAGCAGGTGCTGGTG

CTGCTCCAGCAGGTGCTGCTGCACCAGCAGGTTCTGCTGCTCCAGCAGGTGCTGCTGCTC

CAGCAGGTGCTGCTGGTCCTGTTTCTGGTGCTCCTGCTCCAACTCCGCCTGCTGTTTCTA

GTACTTATGCAGCTACGGTTCAACCTCCACTTGTCAGTGCAAGTGCGTCTGTCCCTACCA

CCATACACCCCTCTCTACAAACTTCAGAACAATCATCTTCATTTGATATTGGAATGTATG

TTGGTATTGCTATAGGTGGTGGTGTAGGTGGTGTTGTAGTTGGTACAATCTTATCTCTTG

TTGGATATCGTGTATATAAAAAACATCTAGATTTAAAATATGTTCCAACACCTGGCAGTG

ATAATATTTAG

>Giros_AGL3_gDNA

ATGAATCTTACTAAGAACACAATCATCTTCGTTCTCTTATGCATCCTTATCGTCTCTAATGTTTTAGCTC

AAGGgtgagtaaattttcaaagtcgtttaatatcgaaaattcaatattattattttttttcattattttt

aaaatataatttttatttttcttgttaaatctaaaagCCCGACACCAACTGGTGAAGCTGGTGCTGCTCC

AACTGGAGCTGGTGCTGGTGGACCTGGTGGAGCTGGTGCTGGTGGACCTGGTGGTGCTGGTGCTGGTGGA

CCTGGTGGTGCTGGTGCTGGTGCTTCTGGTGCTCCTGGTGCTGCTCCAACTGGTGGAGCTGGTGCTGGTG

GACCTGGTGGTGCTGGTGCTGGTGGACCTGGTGGTGCTGGTGCTGGTGGTGCTAGTCCTTCTGGTGCTCC

TGGTGCATCCCCTGCTGCTGCTGGACCGGGTGGAGCTGCTGCCCCATCTGGAACTGGTGCCCCTGGAGCT

TCCCCTGCTGCCGCTGGACCTGGCGCTGCTCCCTCTGGATCATCTCCAAAAGGTGCTGGAGCTGCCCCAA

GTGGTGGAGCTGCCCCAAGTGGTACTGGCGCTGCTAAAGGAGGAGCAAGCCCgtaagtattattttttaa

taaattttttgaataatttattttaatataaattcagtttattaattattatatttcaattgctttttaa

agATCTTCTACAGCCGCAGCTGGTGCTCCCGCTAGTGCTGCTTCAAACGTTTATGTTAACTGCTTTGAAT

TGATCACTGGAACCGTTATTGCTTTATTCTTTGGCACATTATTATATTAA

>Giros_AGL4_gDNA

ATGAATTTCACTAAGAATACAATCCTTTTCCTTGTCTTATGCATCCTTATCGTCTCTAGTGTTCTTGCTC

AAACgtaagttaattttcaaagtcatttaacatcgaaaattcaatattttttcattatttttaaaatatg

tttgcactatcttgttaactctaaaagCCCCAGTGCACCTGCTGGACCTGAAGGAGCTGCACCATCGGAA

TCTGCTGCCCCAGGTGGAAATCCAAAAGCCCCAGCCGGCGCTGCTGCACCAACAGAATCTGCTACCCCAA

GTGGAAATCCAAAAGCCCCAGCCGGCGCTGCTGCCCCATCAGGAACTGCTGCTGCTGCTGGACCTGCCGG

AAGTGCCGCCCCTAGTGGAGCCGCCGGAAAAGCTCCTTCACCTTCTGGAGCCCCAGCTGGTGGTGCAGGT

GGTGCCGGTGGTGCCGGTAAATCAGCAAGTCCTTCTGGgtaagtatttttaataaaacttttgataatta

ttttaattaatttatttattaattattatataacaattgtctttttaaagAACTTCACAATCCGCATCAC

CATCAAGCAGTCCTGCCAAATCTGGTGCTTCAAGCATTCATGTTGAATTGTTCGCTGGATCCGTTATTGC

CTTATTCTGTGGTGCATACTTATATTAA

>Giros_AGL1_partial

MIITKNTIILFFVCILIASGVFA

QDETTSPAAGADATGAGGAGAAPTGGAGGAAPKGGAA

PTGDAGGAAPKGGAAPTSSGAAPAASGGAAPAASGGAAPAGAGGAAPSASPSGGAAKGA

>Giros_AGL2

MSRLIYCLTLLFLVFLVFIRSATS

QNSSKGAATPAAGPAGAASPVPTPASAAAPAGAGAA

PAGAAAPAGSAAPAGAAAPAGAAGPVSGAPAPTPPAVSSTYAATVQPPLVSASASVPTTI

HPSLQTSEQSSSFDIGMYVGIAIGGGVGGVVVGTILSLVGYRVYKKHLDLKYVPTPGSDN

I

>Giros_AGL3

MNLTKNTIIFVLLCILIVSNVLA

QGPTPTGEAGAAPTGAGAGGPGGAGAGGPGGAGAGGP

GGAGAGASGAPGAAPTGGAGAGGPGGAGAGGPGGAGAGGASPSGAPGASPAAAGPGGAAA

PSGTGAPGASPAAAGPGAAPSGSSPKGAGAAPSGGAAPSGTGAAKGGASPSSTAAAGAPA

S

AASNVYVNCFELITGTVIALFFGTLLY

>Giros_AGL4

MNFTKNTILFLVLCILIVSSVLA

QTPSAPAGPEGAAPSESAAPGGNPKAPAGAAAPTESA

TPSGNPKAPAGAAAPSGTAAAAGPAGSAAPSGAAGKAPSPSGAPAGGAGGAGGAGKSASP

SGTSQSASPSSSPAKS

GASSIHVELFAGSVIALFCGAYLY

(u) Rhcer, NCBI WGS BLASTn (gDNA then protein)

>Rhcer_AGL1_QZLG01000098.1:55950 to 56672

ATGAAATACAACACACGTATCTCCCTTCTTCT

CGCTGTTCTCTTTATCTTGGTAACTTATGTTGCTGCACAgtaagttttcctacaaatttt

tattcttttttgttaaatcatttgaatttttctaaacgttttgcttctcttaatatttga

taatagAGCTCCCCCAGATGCTGGAGCAGGAGGTGCCACACCACCACCAGATGCTGGAGC

AGGAGGTGCCACACCACCCCCAGATGCTGGAGCAGGAGGTGCCACACCCCCAGATGCCGG

AAAAGCCCCAGCTGGTGGTGCCCCAGGTGACGCAGGAAAAGCCCCAGCTGGTGGTGCCCC

AGCCCCAGATGCCGGAAAAGCCCCAGCTGGTGGTGCCCCAGCCCCAGATGCAGGAAAAGC

CCCAGCAGCAGGTGCCCCAGATGCCGGAAAAGCCCCAGCAGCAGGTGCCCCAGATGCAGG

AAAAGCCCCAGCAGGAGGTGCCACCCCACCAGCAGGAGGTGCCGCCCCACCAGCAGGAGG

TGCAACCCCACCAGCAGGAGGTGCCGCCCCACCAGCAGGAGGTCCCGCCGGAAAAACACC

AGCAGGAGGTGCCACCCCACCAACAGCTCCATCAGCCGGTGCCCCAGGTGCCTCAGCTTC

ACCTAAAGCAGCAGCAGCAAGCAGTGGAAATACTCTTAAATCCGGAATTTCATTCGCTGC

CATTGCCGTTCTTGGTGCTCTCCTCGCTTAA

>Rhcer_AGL2_ QZLG01000660.1:23922 to 24438

ATGGCAAAATTCACCAAATTAACTTTCGCATGTCTCTTCGTTCTCGTCCTTCTTGTTTCC

TTCACTGTATCTGgttagtaaattattacaatttcatttttcttttttctggttataaaa

aaaattcatttatttaatttatttattttttttaaaatcaacagAAAAATTAGCTGTTCG

TCAAGCAGGAGGAGCAGCCCCACCAGCAGCAGGAGGAGCACCACCAGCAGGAGGAGCAAC

CCCACCAGCAGGAGGTGCAGGAGGAGCAGCCCCACCAGCAGGAGGTGATGCAGGTGCAGG

AGGAGCAACCCCACCAGCTGGAGGAGCTGGAGGAGCAACCCCACCAGCTGGAGGAGCAAC

ACCACCAGGAGGTGCAGCATCATCACCAGCAGTGCCATCATCAACTGGAGCTGCAACAGC

ACCATCAACATCTGCTACTCCAAGTGGTGCCTTCAAAGTTGAAAGTAGTTTAATCAGTGT

TGCCGCCATTGCTGCTATTGTCGGTTTTTTCTTGTAA

>Rhcer_AGL3_ QZLG01000098.1:53657 to 54422

ATGAAATATAATACACGTATCTCACTTCTTCTCGCTGTTCTCTTCGTTTTGGTAGCTTAC

GTTGCCGCACA

gtaagttttcctacaaaatatgaatttttgtaaattctcaaaaaaaaaatt

tttttaaatggtttattattaataaaaaaattttctccttttcttttattaataacagAG

GTCCAGGAGGAGGACCACCACCACCTGGAGGAGCACCAGCAGGAGGACCACCACCACCAG

GAGGAGCACCAGGAGGAGGACCACCACCACCAGGAGGAGCACCACCAGCTGGAGGAGCAC

CAGGAGGAGGAGCACCACCAGCTGGAGGAGCACCAGGAGGAGGAGCACCACCAGCAGGAG

GAGCAAAAGGAGGAGCAGCACCAGCTGGACCAAAGGGTAAAGTTACTCCACCAGCAGGCG

GAGCAGGAGGAGCCCCAGCAGGAGGAGCACCAGCAGGCGGAGCACCACCAGCAGGAGGAG

CACCACCACCAGCAGGAGGAGCACCACCACCAGGAGGAGCACCACCAGCAGGAGGAGCAC

CACCACCAGGAGGAGCACCACCACCAGAAGGAGCAACACCATCAGCAGGAGCTCCAGGAG

GAGGAGCACCAACTCCAGGTCCCGGTACAGGAACCACACCACCAGGAGGAGCAGGAGGAG

CACCTTCAACTTCTCCCAAAACAGCAGCAGCAAGCAGTGGAAATACTCTTAAATCCGGAA

TTTCATTCGCTGCTGTTGCCATGTTTGGTACTTTATTCGTTTAA

>Rhcer_AGL1

MKYNTRISLLLAVLFILVTYVAA

QAPPDAGAGGATPPPDAGAGGATPPPDAGAGGATPPD

AGKAPAGGAPGDAGKAPAGGAPAPDAGKAPAGGAPAPDAGKAPAAGAPDAGKAPAAGAPD

AGKAPAGGATPPAGGAAPPAGGATPPAGGAAPPAGGPAGKTPAGGATPPTAPSAGAPGAS

ASPKAAAAS

SGNTLKSGISFAAIAVLGALLA

>Rhcer_AGL2

MAKFTKLTFACLFVLVLLVSFTVS

EKLAVRQAGGAAPPAAGGAPPAGGATPPAGGAGGAA

PPAGGDAGAGGATPPAGGAGGATPPAGGATPPGGAASSPAVPSSTGAATAPSTSATPS

GAFKVESSLISVAAIAAIVGFFL

>Rhcer_AGL3

MKYNTRISLLLAVLFVLVAYVAA

QGPGGGPPPPGGAPAGGPPPPGGAPGGGPPPPGGAPP

AGGAPGGGAPPAGGAPGGGAPPAGGAKGGAAPAGPKGKVTPPAGGAGGAPAGGAPAGGAP

PAGGAPPPAGGAPPPGGAPPAGGAPPPGGAPPPEGATPSAGAPGGGAPTPGPGTGTTPPG

GAGGAPSTSPKTAAAS

SGNTLKSGISFAAVAMFGTLFV
